# Identification of new genes associated to senescent and tumorigenic phenotypes in mesenchymal stem cells

**DOI:** 10.1038/s41598-017-16224-5

**Published:** 2017-12-19

**Authors:** Joana Cristina Medeiros Tavares Marques, Déborah Afonso Cornélio, Vivian Nogueira Silbiger, André Ducati Luchessi, Sandro de Souza, Silvia Regina Batistuzzo de Medeiros

**Affiliations:** 1 Faculdade de Ciências da Saúde do Trairi (FACISA), Universidade Federal do Rio Grande do Norte (UFRN), Rua Traíri, S/N, Centro, Santa Cruz, Rio Grande do Norte (RN) 59200-000 Brazil; 2 Laboratório de Biologia Molecular e Genômica, Centro de Biociências, UFRN, Campus Universitário, Avenida Senador Salgado Filho, 3000, Lagoa nova, Natal, RN 59078-900 Brazil; 3 Departamento de Análises Clínicas e Toxicológicas, Centro de Ciências da Saúde, CCS/UFRN, Av General Cordeiro de Farias S/N, Petropolis, Natal, 59010-115 RN Brazil; 4 Instituto do Cérebro, Instituto de Metrópole Digital, UFRN, Av. Nascimento de Castro, 2155, UFRN, 59056-450 RN Brazil

## Abstract

Although human mesenchymal stem cells (hMSCs) are a powerful tool for cell therapy, prolonged culture times result in replicative senescence or acquisition of tumorigenic features. To identify a molecular signature for senescence, we compared the transcriptome of senescent and young hMSCs with normal karyotype (hMSCs/n) and with a constitutional inversion of chromosome 3 (hMSC/inv). Senescent and young cells from both lineages showed differentially expressed genes (DEGs), with higher levels in senescent hMSCs/inv. Among the 30 DEGs in senescent hMSC/inv, 11 are new candidates for biomarkers of cellular senescence. The functional categories most represented in senescent hMSCs were related to cellular development, cell growth/proliferation, cell death, cell signaling/interaction, and cell movement. Mapping of DEGs onto biological networks revealed matrix metalloproteinase-1, thrombospondin 1, and epidermal growth factor acting as topological bottlenecks. In the comparison between senescent hMSCs/n and senescent hMSCs/inv, other functional annotations such as segregation of chromosomes, mitotic spindle formation, and mitosis and proliferation of tumor lines were most represented. We found that many genes categorized into functional annotations related to tumors in both comparisons, with relation to tumors being highest in senescent hMSCs/inv. The data presented here improves our understanding of the molecular mechanisms underlying the onset of cellular senescence as well as tumorigenesis.

## Introduction

Human mesenchymal stem cells (hMSCs) are used in cellular therapy because they are easy to obtain and expand *in vitro*, owing to their functional plasticity. In addition, they secrete bioactive molecules which play roles in immunomodulation, chemotaxis, and neuroprotection, as well as have trophic effects and other functions in tissue repair^1–3^.

In the organism, stem cell senescence might cause a functional decline, contributing to the development of metabolic and degenerative diseases, as well as cancer and other age-related diseases^4–7^.

During cell culture, cells may undergo molecular changes resulting in the acquisition of a senescent phenotype and favoring tumorigenic processes. Senescent hMSCs are able to secrete many factors which increase the inflammatory response and proliferation and migration of cancer cells^8^. Prolonged cell culture time may also contribute to the incidence of chromosomal instability without leading to spontaneous malignant transformation^9–11^, but may be intimately associated with the progression of senescence^11–14^.

The replicative senescence acquired with extended *in vitro* cultivation is analogous to *in vivo* aging^15^. The senescence process occurs from the beginning of the culture and progresses with each passage of the culture. Although phenotypic and molecular characteristics of senescent cells have already been described^16–18^, cell culture time and different sources of cells can result in molecular differences in the senescence process that may aid understanding of the relation of the senescence phenotype to age-related diseases and tumorigenesis. Therefore, molecular analysis by expression profiling of hMSCs cultivated for long periods can identify new markers of senescence and the tumorigenic phenotype; this would be useful in monitoring cultured hMSCs to detect cells with phenotypes that may decrease efficiency of cell therapy and promote undesirable clinical effects.

Transcriptome studies of hMSCs have focused on differential expression patterns among cells obtained from different sources^19–26^, different stages of the differentiation process^27–30^, and different cultivation times^31–35^. Differentially expressed genes have already been identified in bone marrow stem cells (hBMSC) at the 20^th^ passage compared to the 1st passage, adipose tissue stem cells (ASCs) at the 30th passage compared to the 1st passage^31^, hBM-MSC at the 15th passage compared to the 7th passage^32^, umbilical cord mesenchymal stem cells (UC-MSC) at the 15th passage compared to the 3rd passage^33^, hBMSCs at 33 population doubling levels (PDL) compared to 3 PDL^34^, and in BMSC at the 15th passage compared to 10th passage^35^. However, none of these studies evaluated the gene expression profile of senescent hMSCs derived from umbilical cords at the 18th passage compared to the 3rd passage, nor the constitutional chromosomal alterations, as we report here. We propose a model of senescence in which differentially expression genes (DEGs) are new candidates for markers of senescence in hMSCs; we also discuss others DEGs potentially related to the tumorigenic potential of senescent mesenchymal stem cells.

## Materials and Methods

### Human mesenchymal stem cell source

Human mesenchymal stem cells (hMSCs) were extracted from the umbilical cord veins of three donors and were collected in the Maternidade Escola Januário Cicco (Januário Cicco Maternity Hospital) of the Federal University of Rio Grande do Norte (UFRN). Collection was approved by the Committee for Ethics in Research of the UFRN under protocol no. FR132464, and informed consent was obtained from all participants. All experiments were performed in accordance with relevant guidelines and regulations.

The hMSC karyotypes were as follows: donor 1, normal karyotype (46,XY); donor 3, normal karyotype (46,XX) – cells from both lineages were named hMSCs/n; donor 2, karyotype with constitutional chromosome inversion (46,XY,inv(3)(p13p25))^36^ – named hMSCs/inv. The hMSCs/inv and hMSCs/n were isolated, expanded, and phenotyping was performed by flow cytometry as described by Duarte *et al*.^36^.

### hMSC culture

The cells were plated in T25 flasks and cultured in alpha-MEM (Gibco, Franklin Lakes, NJ, USA), supplemented with 10% Fetal Bovine Serum (FBS) (Gibco) and 1% antibiotic solution (penicillin and streptomycin; Sigma-Aldrich, St. Louis, MO or Gibco), and maintained at 37 °C in a humidified incubator with 5% CO_2_. The viable cells (identified by trypan blue staining; Gibco) were passaged when they reached ∼60–70% confluence using 1 mL of 0.02% trypsin/0.25% EDTA (Invitrogen, CA, USA) and seeded at a concentration of 4000 cells/cm^2^ on a culture plate.

The hMSCs/n (from donor 1) and hMSCs/inv (from donor 2) cells were expanded six times on individual plates until the 24^th^ passage, after which it was not possible to start a new subculture. The cells derived from donor 3 were expanded three times on individual plates until the 9^th^ passage. There were 9 total plates from young cultures (passage 9): 6 replicates of plates from donor 1 and 3 replicates of plates from donor 3. There were 6 total plates from the senescent culture (passage 18) from donor 1. RNA extracted from each plate was used in microarray analysis.

The medium was changed 2–3 times per week and cells in the 9^th^ (young cells) and 18^th^ passages (senescent cells) were used for assays. The cells were monitored daily with a CKX41 inverted microscope (Olympus, Tokyo, Japan).

### Immune phenotype

Both the hMSC/n and hMSC/inv were phenotypically characterized by flow. Fluorescein isothiocyanate (FITC)- or phycoerythrin (PE)-conjugated monoclonal antibodies specific to CD90, CD105 (Bioscience, San Diego), CD73, CD14, CD34, CD38, CD45, and HLA-DR (Becton Dickinson’s, CA, USA) were used. Appropriate, isotype-matched, non-reactive fluorochrome-conjugated antibodies were employed as controls (IgG1-FITC and PE-Cy5; Becton Dickinson’s). Analysis of cell populations was performed using a Fluorescence-activated cell analyzer (FACScan, CA, USA), and data were calculated using the Cell Quest software (Becton Dickinson’s, CA, USA). The results were displayed as percentages of cells labeled for each monoclonal antibody. This protocol was performed as described by Duarte *et al*.^36^.

### Characterization of the senescent hMSCs

Senescent cells were viewed with a CKX41 inverted microscope (Olympus), and characteristics such as increased cell size, morphology changes, increased presence of vacuolated cells, decreased proliferative capacity, and β-galactosidase activity (at pH 6.0) were observed (Chemicon, USA; conducted following the manufacturer’s instructions at 60% confluence in triplicate). In passage 18, ≥80% of the cells were reactive for senescence-associated β-galactosidase.

### Microarray experiment and data analysis

Total RNA was extracted from 1 × 10^6^ cells (~90% confluence) using the RNeasy Mini Kit (Qiagen Nordic, West Sussex, UK) and following the manufacturer’s protocols. Gene expression profiles were determined using the GeneChip^®^ Human Genome U133 Plus 2.0 array (Affymetrix, Santa Clara, CA), which contains probe sets for over 47,000 transcripts. Profiling was performed in the National Synchrotron Light Laboratory in Campinas, São Paulo, Brazil, according to the manufacturer’s recommendations. Using GC-RMA (Robust Multiarray Average), Affymetrix.cel files were uploaded in the Partek^®^ Genomics Suite^®^ version 6.5 (Partek, MO, USA) and normalized. Statistical significance was determined using a one-way analysis of variance (ANOVA) and concomitant post hoc tests implemented in Partek^®^ Genomic Suite (*P* < 0.001) with a Bonferroni correction and a |log_2_(fold-change)| ≤3.0 and ≥3.0.

The comparisons made between gene expression profiles were as follows:

- Young hMSCs/n vs. senescent hMSC/n: 9 microarray expression profiles of young cells (young hMSCs/n) at passage 9 (6 from donor 1 and 3 from donor 3) were compared to 6 microarray expression profiles of senescent cells (senescent hMSC/n) at passage 18 (from donor 1).
- Young hMSCs/inv vs. senescent hMSCs/inv: 6 microarray expression profiles of young cells (young hMSC/inv) at passage 9 (from donor 2) were compared to 6 microarray expression profiles of senescent cells (senescent hMSC/inv) at passage 18 (from donor 2).
- Young hMSCs/n vs. young hMSCs/inv
- Senescent hMSCs/n vs. senescent hMSCs/inv

Functional category searches were applied to all of the differentially expressed genes (DEGs) with the Ingenuity Pathway Analysis Application (IPA) (Ingenuity Systems, Redwood City, CA, USA), using the default software parameters for human species.

Microarray data are described in accordance with GEO and MIAME guidelines (www.ncbi.nlm.nih.gov/geo/info/MIAME.html) and were deposited in the GEO database (http://www.ncbi.nlm.nih.gov/projects/geo/) as series GSE56530.

### Evaluation of differential expression by qRT-PCR

To validate differential gene expression from the microarray analysis, 11 genes were selected for qRT-PCR analysis based on biological relevance as suggested by the Ingenuity Pathway Analysis (IPA). The same RNA extracted for microarray analysis was used for PCR analysis in triplicate assays of all young and senescent cells: young hMSCs/n, senescent hMSCs/n, young hMSCs/inv, and senescent hMSCs/inv. Furthermore, the PCR experiments were performed in duplicate.

The cDNA was prepared with 1 µg of total RNA using the High-Capacity cDNA Reverse Transcription Kit (Applied Biosystems, Foster City, CA) with a Veriti™ 96-Well Fast Thermal Cycler (Applied Biosystems). cDNA (200 ng) was used for Inventoried TaqMan® Gene Expression Assays (Applied Biosystems) according to the manufacturer’s protocols. Gene expression analysis was performed with the relative quantification method.

Quantification and normalization of expression levels of the target genes and the reference gene (*YWHAZ*) were calculated using the comparative threshold cycle (*C*_T_) method. The data were analyzed using Sequence Detection Software v. 2.0.1 (Applied Biosystems). *YWHAZ* expression displayed a low coefficient of variation across all tested samples according to the geNorm software. Its M value was 0.142, and Wang *et al*.^23^ identified this gene as the best endogenous gene for analyses of hMSCs derived from the umbilical cord.

All assay probes were synthesized by the inventoried probes service with carboxyfluorescein (FAM) labelling (Applied Biosystems): LAMC2 (Hs01043711_m1), ANKRD1 (Hs00173317_m1), KYNU (Hs00187560_m1), MMP1 (Hs00899658_m1), MAB21L1 (Hs00366575_s1), SFRP1 (Hs00610060_m1), NDN (Hs00267349_s1), ADORAB2 (Hs00386497_m1), CCL7 (Hs00171147_m1), G0s2 (Hs00274783_s1), ALDH1A1 (Hs00946916_m1), YWHAZ (Hs03044281_g1). Primers were used at an annealing temperature of 60 °C in all qRT-PCR assays.

The significance of the results was determined by the t-test at a significance level of *P* < 0.05.

### Systems biology analysis

Interaction networks were constructed using STRING version 9.0 (Search Tool for the Retrieval of Interacting Genes/Proteins)^37^. For each list of DEGs, a network with the following parameters was built: organism, *Homo sapiens*; average confidence; other, ‘default.’ All prediction methods were active, including ‘neighborhood,’ ‘gene fusion,’ ‘co-occurrence,’ ‘co-expression,’ ‘experiments,’ ‘databases,’ and ‘textmining.’ Subsequently, the summarized.txt file generated by STRING was used and exported to Cytoscape 2.8.2^38^. For the functional categorization of genes belonging to networks, the BiNGO 2.44 plugin^39^ was used with a hypergeometric distribution and correction for multiple tests using the False Discovery Rate (FDR) algorithm with a significance level of *P* < 0.05. *Homo sapiens* was the selected organism. The most enriched categories were those that presented the lowest *P*-values. DEGs were sorted according to the number of genes; categories with a biological representation of ≤5% of molecules were excluded.

The search for bottlenecks was performed with the CestiScaPe 1.21 plugin to identify the betweenness and node degree values of the nodes present in the networks. These values were exported to the GraphPad software to build a graph of betweenness (X-axis) versus node degree (Y-axis) using the column statistic test.

## Results and Discussion

### Characterization of young and senescent hMSCs

**Figure 1:**
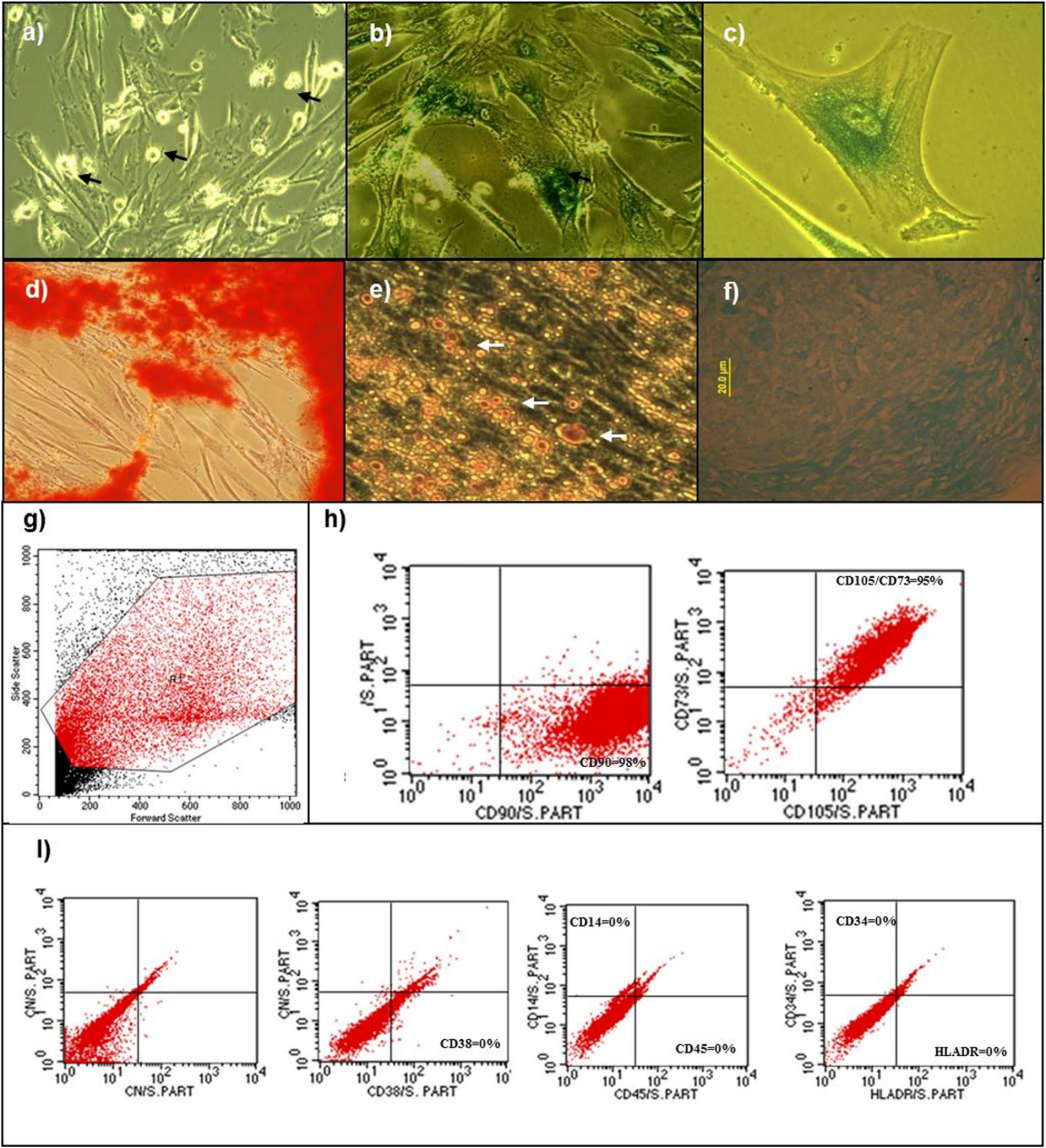
Phenotypic characterization of hMSCs. (**a**) Cells at 9th passage (young cells) with fibroblastoid morphology. (**a**) The arrows indicate dividing cells (20×). (**b**) Cells at 18th passage (senescent cells) form polygonal shapes and ≥80% of the cells are stained green by the β-galactosidase assay (20×). (**c**) Senescent cells display vacuolated and granular cytoplasm (40×). (**d**) Osteogenic differentiation. Stained matrix with alizarin red (20×). Fatty vacuoles stained with oil red O (40×). (**f**) Chondrogenic differentiation. Stained matrix with alcian (20×). Flow cytometry characterization of hMSCs: (**g**) Dot-plot as a graphic representation of the colored cells by FACS analysis. The highlighted red region (R1) indicates the gated region of the cell population analyzed. (**h**) Cells positive for the mesenchymal surface markers CD90 and CD105/73. (**i**) Cells negative for the hematopoietic markers CD38, CD14/CD45, and CD34/HLDR.

Young hMSCs (passage 9) did not show characteristics of senescence, exhibited a high population of dividing cells, showed morphology typical of hMSCs, and did not have any β-galactosidase activity (Fig. 1a). Senescent cells (passage 18) had β-galactosidase activity, exhibited a large and granular cytoplasm, and underwent only a few cellular divisions (Fig. 1b and c). The senescent cells characteristics and the cultivation time required to culture the senescent cells were similar to those described for hMSCs obtained from different sources^8,31,40,41^.

After passage 18, activity of the subcultures gradually decreased, and it was impossible to obtain enough cells for RNA extraction. The cells were expanded until the 24th passage, remaining viable for two months, at which point it was impossible to continue subculturing. No spontaneous hMSC transformation was observed for either hMSCs/inv or hMSCs/n. These results were consistent with results from several studies of hMSCs^42–45^.

Young cells from both hMSCs/inv and hMSCs/n were able to differentiate into osteoblasts (Fig. 1d), adipocytes (Fig. 1e), and chondrocytes (Fig. 1f). Senescent cells were able to differentiate into osteogenic and adipogenic lineages. Induction of chondrogenic differentiation was not performed because of the reduced number of cells available for the assays. These results are consistent with those of Nekanti  *et al*.^41^  and Huang *et al*.^42^, who analyzed hMSCs derived from bone marrow (BM) at passage 30 and from Wharton’s jelly (WJSC) at passages 15–20, respectively.

Both young and senescent hMSCs/inv and hMSCs/n presented cellular markers specific to hMSCs (CD73, CD90, and CD105) (Fig. 1h). These markers were also reported to be found in hMSCs obtained from WJSCs at passages 15–20^41^ and from umbilical cord-derived hMSCs at passage 15^44^.

### Changes in the expression profile of young and senescent hMSCs

This is the first study to evaluate DEGs of hMSCs obtained from the umbilical cord vein undergoing senescence at the 18^th^ passage with and without constitutional chromosomal alterations.

**Figure 2:**
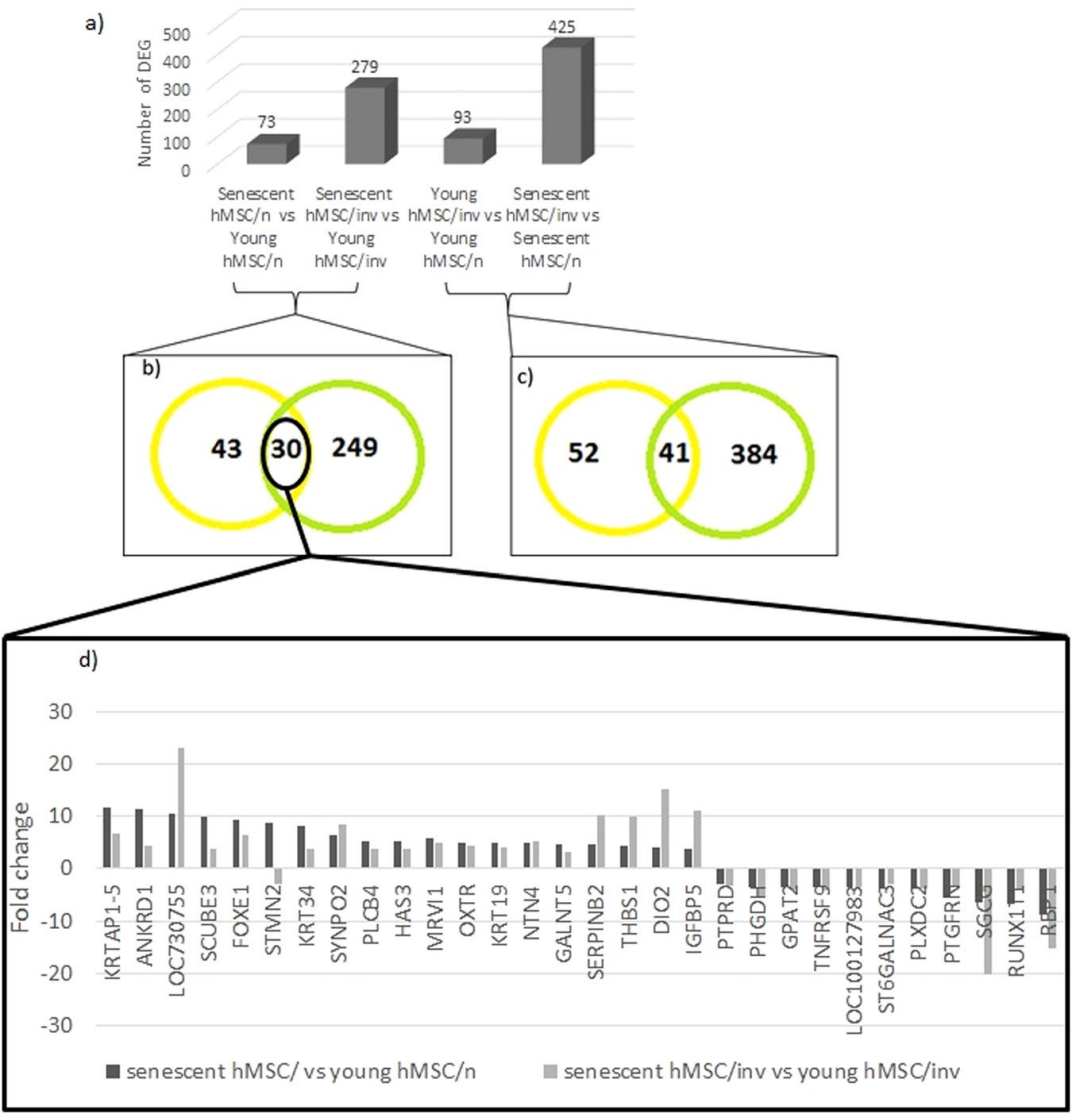
Differentially expressed genes (DEG) in all comparisons. (**a**) The 73 DEGs in senescent compared with young hMSCs/n, 279 DEGs in senescent compared with young hMSCs/inv, 93 DEGs in young hMSCs/inv compared with young hMSCs/n, 425 DEGs in senescent hMSCs/inv compared with senescent hMSCs/n. (**b**,**c**) Venn diagram showing the common DEG between comparisons. (**b**) There were 30 common DEGs between senescent and young types of both hMSCs/n and hMSCs/inv, with 11 genes upregulated in both senescent cells being new candidates for senescent markers. (**d**) The most common DEGs were upregulated in both cell types, but some genes such as *LOC730755*, *THBS1*, *SERPINB2*, *DIO2*, and *IGFBP5* showed higher expression in senescent hMSCs/inv.

We identified 73 DEGs in senescent compared to young hMSCs/n (Fig. 2a, see Supplemental file 1 for full table) and 279 DEGs in the senescent compared to young hMSCs/inv (Fig. 2a, see Supplemental file 2 for full table); the number of DEGs was greater between the hMSCs/inv groups (Fig. 2a).

There were 30 DEGs found in both comparisons (senescent vs. young hMSCs/n and senescent vs. young hMSCs/inv) (Fig. 2b). Among them, 18 were upregulated in both types of senescent hMSCs (Fig. 2d, see Supplemental file 3). These data demonstrate a molecular signature of senescence common to both hMSC/n and hMSC/inv. Of the 18 upregulated genes, 11 are novel candidate markers of senescence (*DIO2, FOXE1, GALNT5, HAS3, KRT19, KRT34, KRTAP1-5, LOC730755, MRVI1, PLCB4*, and *SCUBE3*). For most, the change in expression was similar in hMSCs/inv and hMSCs/n; however, some (*LOC730755, THBS1, SERPINB2, DIO2*, and *IGFBP5*) showed higher expression in the senescent hMSCs/inv (Fig. 1d). Among the 18 upregulated, 6 genes (*ANKRD1*, *NTN4*, *OXTR*, *SERPINB2*, *SYNPO2* and *THBS1*) have already been related to the senescence in *in vitro* Bone Marrow^32^. *ANKRD1* has already been related to the senescence *in vivo* of hMSC from bone marrow of older donors^46^, and *IGFBP5* was upregulated in senescent cells^47,48^. In the list of 279 differentially expressed genes in the senescent hMSC/inv compared young cells, 22 (*AGTR1*, *ANK2*, *ANKRD* *1*, *BACE2*, *DAB2*, *HGF*, *JAM2*, *LTBP2*, *MOCOS*, *NMI*, *NRP1*, *NTN4*, *OXTR*, *PLXDC2*, *SERPINB2*, *SERPINE1*, *SNCAIP*,* SPOCD1*,* ST6GAL1*, *SYNPO2*, *THBS1*, *TM4SF1*) have also been identified as related to *in vitro* senescence^32^, and 10 genes (*RGS4*, *ANKRD1*, *NRXN3*, *DDIT4*, *C1R*, *PDE4DIP*, *GAS1*, *CXCL12*,* FST*, *C1S*) also has been related to aging *in vivo* ^46^, and *IGFP5* and *SERPINE1* was associated to senescence cell from bone marrow and adipose tissue^47^.

There were 93 DEGs in young hMSCs/inv compared to young hMSCs/n (Fig. 2a, see Supplemental file 4) and 425 DEGs in senescent hMSCs/inv compared to senescent hMSCs/n (Fig. 2a, see Supplemental file 5 for full table). Among them, 41 genes were also differentially expressed when comparing young hMSCs/inv and young hMSCs/n (Fig. 2c). These results suggest that hMSCs/n and hMSCs/inv exhibit intrinsic molecular differences unrelated to culture stage. Such differences were exacerbated with cultivation time, as greater gene expression changes were observed among the senescent hMSCs/inv.

The gene expression differences detected by microarray analyses were validated by qRT-PCR using 11 representative genes exhibiting statistically significant changes in expression (*P* < 0.05) (see Supplemental file 6). DEGs compared by microarray analysis and confirmed significant by qRT-PCR were as follows: *ANKRD1* and *MMP1* in senescent hMSC/n vs. young hMSC/n; *SFRP1*, *ANKRD1*, *G0S2*, and *NDN* in senescent hMSC/inv vs. young hMSC/inv; *ADORA2B*, *SFRP1*, *KYN* *U*, *G0S2,* *ALDH1A1*, and *MAB21L1* in young hMSC/inv vs. young hMSC/inv; and *ADORA2B*, *CCL7*, *SFRP1*, *KYNU*, *ANKRD1*, *MMP1*, *LAMC2*, *G0S2*, *MAB21L1*, and* NDN* in senescent hMSC/inv vs. senescent hMSC/n.

### Functional classification of differentially expressed genes (DEGs)

**Figure 3:**
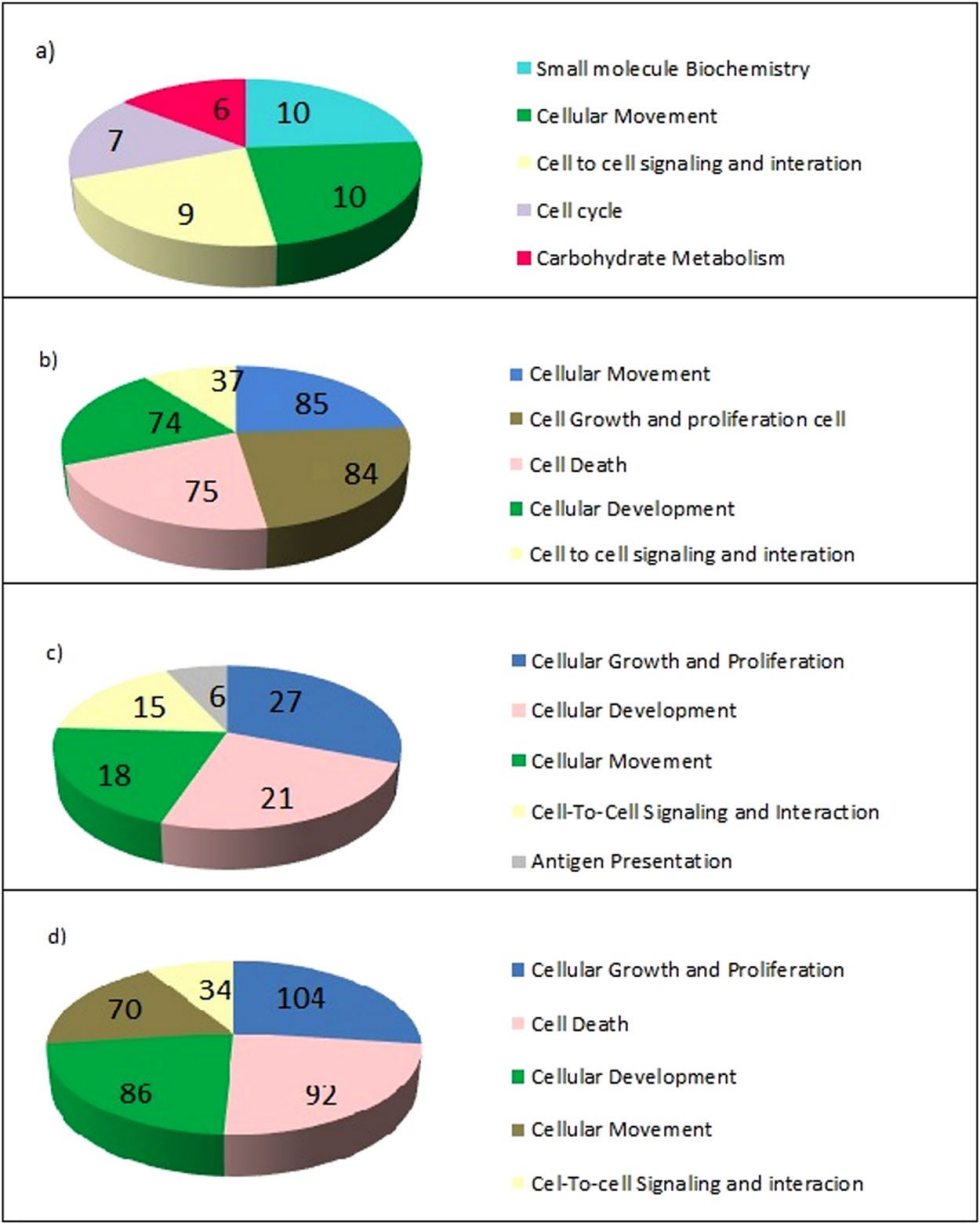
Functional classification of the DEGs in all comparisons. The five categories with the lowest *P*-values indicating the greatest gene enrichment (**a**) Functional classification of the 73 DEGs in senescent hMSCs/n compared with young hMSCs/n. (**b**) Functional classification of the 279 DEGs in senescent hMSCs/inv compared with young hMSCs/inv. (**c**) Functional classification of the 93 DEGs in young hMSCs/inv compared with young hMSCs/n. (**d**) Functional classification of the 425 DEGs in senescent hMSCs/inv compared with senescent hMSCs/n.

The 30 DEGs found in senescent vs. young cells were classified into five functional categories (Fig. 3, see Supplemental files 7–10), cell death, cellular signaling and interaction, embryonic development, cell proliferation and growth, and cellular movement (see Supplemental files 11 and 12). Similarly, metabolism, cellular adhesion, apoptosis, and proliferation were the most represented classes of genes in senescent hMSCs at 33 PDL (population doubling level) obtained from BM^34^. Proliferation, cell adhesion, and development were also among the most represented processes in hMSCs derived from BM at the 15th passage^31^.

For young and senescent hMSCs/inv and hMSCs/n, the categories cell proliferation and growth and cellular movement were the most represented (Fig. 3c and d, see Supplemental files 13 and 14). The functional annotations most represented within these categories were associated with tumors, especially when comparing between senescent cells. Functional annotations of cell proliferation, migration, and adhesion of tumor lineages were also among the most represented (see Supplemental files 15 and 16).

Prediction of function activation analysis performed using IPA showed that the functions of mitosis delay and thymidine incorporation increased in senescent hMSCs/inv compared to senescent hMSCs/n. Corroborating this prediction, DEGs of senescent hMSCs/inv were classified into the cell cycle, mitotic spindle assembly checkpoint, cell cycle stop, endoreduplication, chromosome segregation, and cytokinesis functions. The cellular organization, association, replication, recombination, and DNA repair functional categories were also represented (see Supplemental files 15 and 16).

In this study, the enrichment of DEGs between senescent hMSCs/inv and senescent hMSCs/n in functional categories was associated with tumorigenesis (Supplemental files 16). The molecular relationship between cellular senescence and cancer had already been observed in other studies^49–51^. These genes have been placed in the categories of cell cycle regulation, metabolic processes, and response to DNA damage and apoptosis, among others, suggesting that cancer evolution is strongly associated with aging^6,52^.

### Interaction network of DEGs between senescent and young hMSCs

**Figure 4:**
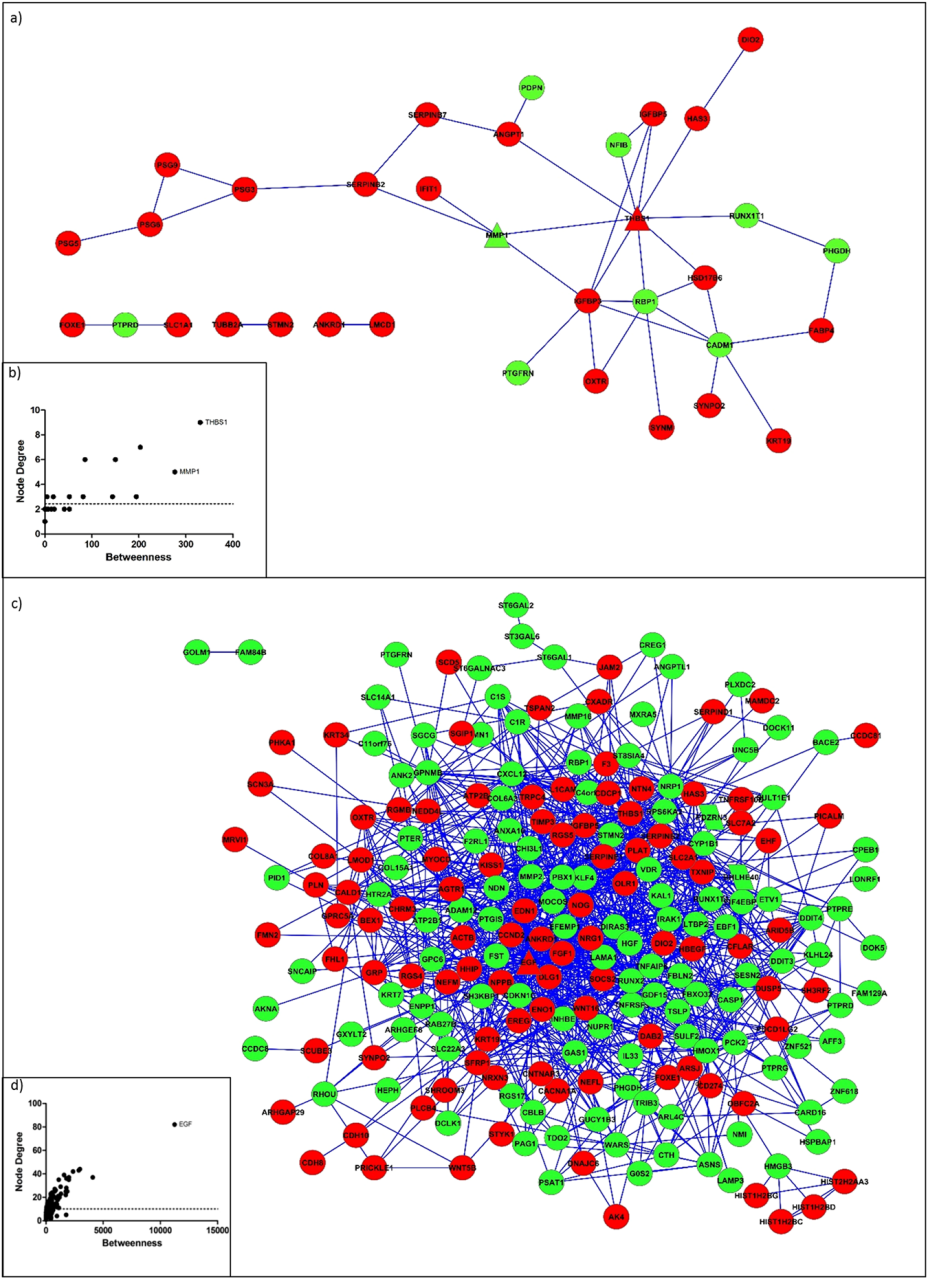
Networks of the DEGs in senescent cells. (**a**) Network of the DEGs in senescent hMSCs/n compared with young hMSCs/n. (**b**) *THSB1* and *MMP1* are the triangle nodes identified as bottlenecks. (**c**) Network of the DEGs in senescent hMSCs/inv compared with young hMSCs/inv. (**d**) *EG*F is the triangle node identified as a bottleneck (black arrow). The red and green colors represent upregulated and downregulated genes in senescent cells, respectively.

Using systems biology analysis, we identified novel interaction networks formed by DEGs from the comparisons between senescent and young hMSCs/n (73 DEGs) and between senescent and young hMSCs/inv (279 DEGs). The genes *THBS1* and *MMP1* were identified as bottlenecks in the former comparison (Fig. 4a and b), and *EGF* was identified as a bottleneck in the latter comparison (Fig. 4c and d).

*THBS1* was overexpressed (FC = 7.33 and *P* = 2.24E^−08^) in senescent hMSCs/n compared to young hMSCs/n and senescent hMSCs/inv compared to senescent hMSCs/n. *MMP1* was overexpressed (FC = 96.63 and *P* = 2.45E^−25^) in senescent hMSCs/inv compared to senescent hMSCs/n. Overexpression of *MMP1* ^18^ and *THBS1* ^31,32,50,53^ were also observed in the oldest mesenchymal stem cells obtained from other sources. Two studies reported elevated *MMP1* expression in all 19 observed cases of giant-cell tumor of the bone (GCTB)^54^, and *MMP1* is associated with increased tumor invasiveness and migration^55^. According to protein-protein interaction predictions, THBS1 interacts with IGFBP5 (score 0.62) (Fig. 4b). THBS1 can bind to the IGFBP5 protein in the extracellular matrix, increasing IGF1-mediated proliferation in smooth muscle cells^56^. *IGFBP5*, in this study, also exhibited higher expression in senescent hMSCs/n and senescent hMSCs/inv than in young cells. It is likely that *MMP1, THBS1*, and *IGFBP5* have important roles in signaling pathways activated during cell cycle control and cell migration in senescent hMSCs.

*EGF* showed higher expression (FC = 6.96 and P = 4.39E^−28^) in senescent hMSCs/inv than in the young hMSCs/inv. The signaling pathway induced by *EGF* appeared to be activated in senescent hMSCs/inv. Of 341 genes transcriptionally activated in response to EGF in HeLa cells^57^, 30 were present among DEGs in senescent hMSCs/inv (compared to young hMSCs/inv): *ARID5B, ASNS, BHLHE40, CDCP1, CTH, CYP1B1, DCLK1, DDIT3, DDIT4, DUSP5, EDN1, EREG, F3, FST, GDF15, GEM, GPRC5A, HBEGF, KLF4, KRT34, NRP1, PICALM, PSAT1, PTPRE, SERPINE1, SESN2, SOCS2, THBS1, TRIB3*, and *WARS* (Fig. 2a, see Supplemental file 4). Some of these genes appear in the interaction network in which *EGF* was identified as a bottleneck (Fig. 4d). The release of EGF by hMSCs has been associated with the development of breast cancer^58^. The increased expression of *EGF* and its receptor were also observed in GCTB and were associated with tumor malignancy^59^. The upregulated expression of several genes associated with TATA-binding protein Associated Factors (TAFs), including *EGF*, have been reported in hMSCs derived from BM but not in hMSCs derived from WJSCs^60^. Therefore, those 30 DEGs in senescent hMSC/inv compared to young hMSC/inv are possibly new candidates that undergo regulation in response to EGF in hMSCs obtained from the umbilical cord.

**Figure 5:**
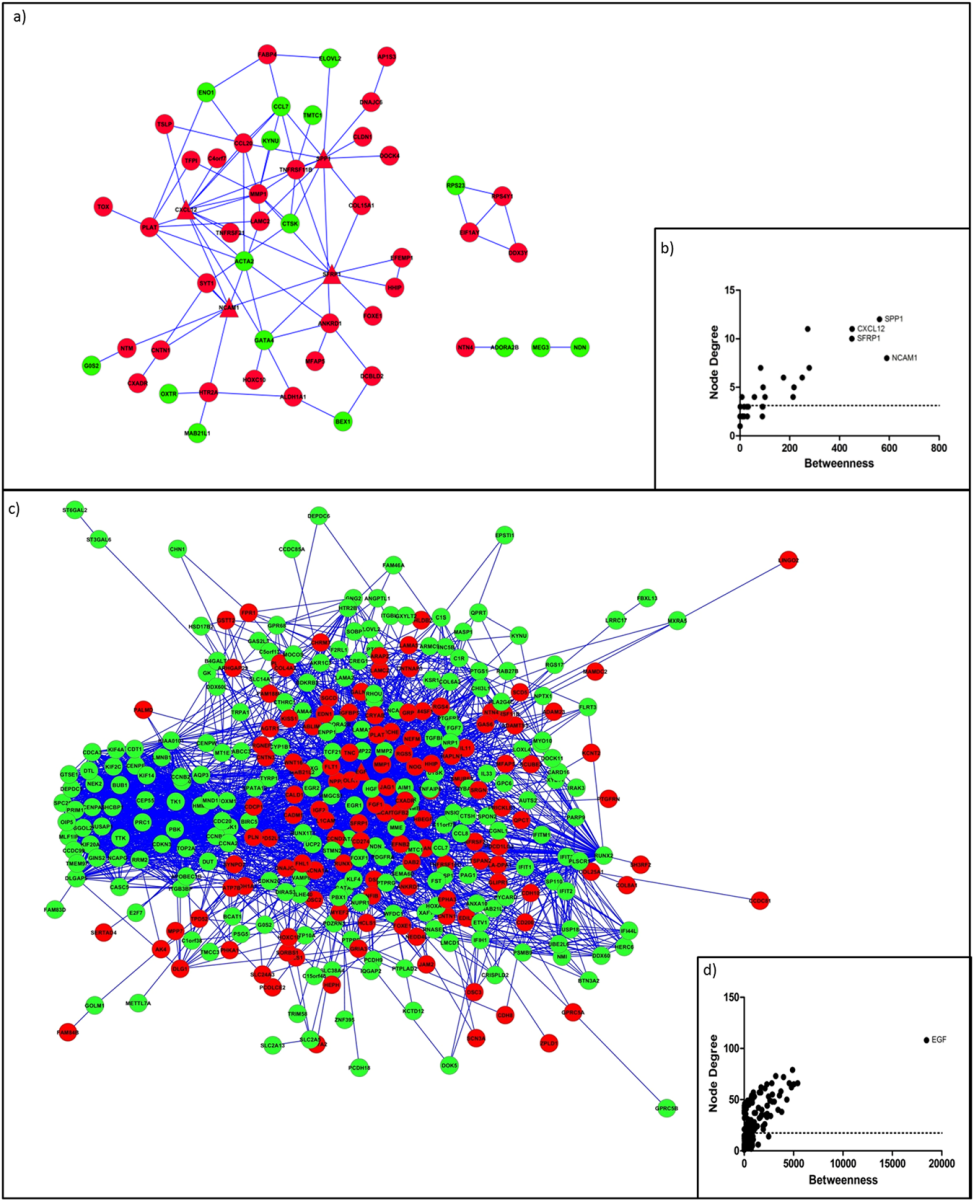
Networks of the DEGs in hMSCs/inv. (**a**) Network of the DEGs in young hMSCs/inv compared with young hMSCs/n. (**b**) *SPP1*, *CXCL12*, *SFRP1*, and *NCAM1* are the triangle nodes identified as bottlenecks. (**c**) Network of the DEGs in senescent hMSCs/inv compared with senescent hMSCs/n. (**d**) *EGF* is the triangle node identified as a bottleneck. The red and green colors represent upregulated and downregulated genes in hMSC/inv, respectively.

The genes *CXCL12, NCAM1*, *SPP1*, and *SFRP1* were bottlenecks in the network of the DEGs in young hMSCs/inv compared to young hMSCs/n (Fig. 5a and b). The gene *EGF* was also identified as a bottleneck in senescent hMSCs/inv compared with hMSCs/n (Fig. 5c and d). The expression of *CXCL12, NCAM1*, and *SPP1* was higher in young hMSCs/inv than in young hMSCs/n.

The functions of cellular movement and adhesion were the most enriched in another study using hMSCs derived from BM stimulated with^61^. It was previously observed that CXL12 can increase the migration of tumor cells^62–65^ and promote the attraction of human stem cells to tumor sites, which contributes to tumor development^66^, including to GCTB development^67,68^.

*NCAM1* expression was previously detected in MSCs^69,70^, corroborating the expression observed in the present study. *NCAM1* is associated with the maintenance of stem cell properties, as well as hMSC migration via MAPK/ERK signaling^69^. However, elevated *NCAM* expression has also been implicated in tumorigenesis in stem cells^70^ and in tumor cells^71–73^.

hMSCs isolated from sites of metastasis and treated with SPP1 showed elevated expression of *CCL5*, cancer associated fibroblast (CAF) markers, *CXCL12*, and metalloproteinases (*MMP2* and *MMP9*)^74^. Furthermore, some studies have suggested a correlation between increased SSP1 expression and malignancy in several tumor types^75–77^. Borrello *et al*.^78^ revealed that cells from papillary thyroid carcinoma display high expression of genes present in the interaction network identified in this study (Fig. 5b). Notably, *SPP1*, *CXCL12*, and *NCAM* were identified as bottlenecks, and *MMP1* was shown to be an important hub in the network.

Increased expression of *SPP1*, *ALP*, and *Runx2* mediated by the activation of ERK1/2 via FGF-2/FGFR-2 signaling has been observed in GCTB stromal cells treated with FGF2^79^. *FGF2* was not differentially expressed in hMSCs/inv, but *FGF1* was upregulated in senescent hMSCs/inv compared to young hMSCs/inv and senescent hMSCs/n. *FGF1* is known to encode a universal ligand of fibroblast growth factor receptors (FGFR)^80^; this may explain the increased *SPP1* expression in young hMSCs/inv. These associations between the expression profile of the hMSCs/inv and the appearance of the CAF phenotype in stromal cells of GCTB support the hypothesis that certain tumors originate from hMSCs with a fibroblastoid phenotype. Numerous metalloproteinases are expressed in stromal cells of GCTB, including *MMP1* and *MMP2* ^54^. *MMP1* was highly expressed in young (FC = 8.79 and *P* = 7.99E^−11^) and senescent (FC = 96.6 and *P* = 2.45E^−25^) hMSCs/inv compared to hMSCs/n. In contrast, *MMP2* and *MMP16* showed reduced expression in the senescent hMSCs/inv (FC = −4.02 and *P* = 1.70E^−13^). Si *et al*.^54^ observed elevated *MMP1* expression in all 19 studied cases of GCTB, as well as reduced induction of *MMP2*.

Overexpression of *SFRP1* was found in the senescent compared to young hMSCs/inv and compared to hMSCs/n, and was validated by qRT-PCR (supplemental file 6). Interestingly, this gene was also upregulated in MSCs obtained from murine BM, inducing their migration to the veins^81^. Transcriptome analysis of GCTB tumor cells revealed DEGs with an enrichment of genes associated with osteogenesis and osteoclastogenesis such as *SFRP4*, a modulator of the WNT pathway^82^.

**Figure 6:**
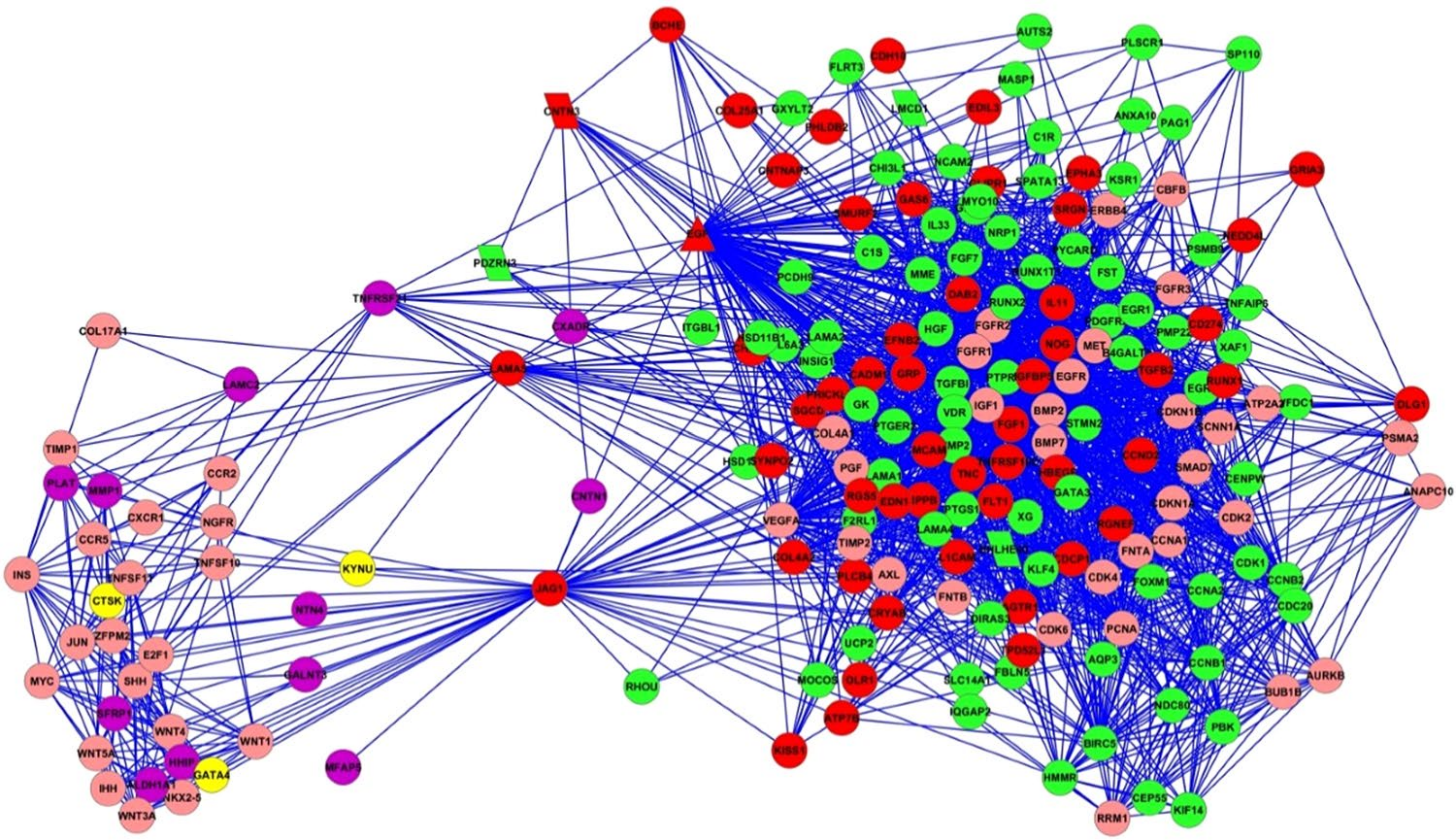
Networks of the common DEGs between young hMSCs/inv vs. hMSCs/n and senescent hMSCs/inv vs. hMSCs/n. The red and green colors represent upregulated and downregulated genes in senescent hMSCs/inv compared with senescent hMSCs/n, respectively. The purple and yellow colors represent upregulated and downregulated genes in young hMSCs/inv compared with hMSCs/n, respectively. The pink color represents genes from the human genome databases that are connected with genes that did not show expression changes. *TNFRSF21*, *LAMA5*, and *JAG1* are the triangle nodes identified as hubs connected with the bottleneck *EGF*. *PDZRN3* and *CNTN3* are parallelogram nodes localized in the region of the chromosomal inversion of hMSCs/inv.

The network connecting DEGs in senescent hMSCs/inv vs. senescent hMSCs/n and young hMSCs/inv vs. young hMSCs/n (Fig. 6) displayed *LAMA5* and *JAG1* interacting with *EGF* and its connected genes. These genes were overexpressed in senescent hMSCs/inv compared to senescent hMSC/n, and EGF was also differentially expressed between young hMSCs/inv and young hMSCs/n. From the 341 genes transcriptionally responsive to EGF induction^57^, 20 genes were present in this network.

*LAMA5* and *JAG1* are located on chromosome 20 (20q13.2–q13.3 and 20p12.1–p11.23, respectively). Senescent hMSCs/inv gained genetic material in the long arm of chromosome 20 (karyotype: 46,XY,inv(3)c, add(20)(q13.3)) and telomeric associations involving 20q13 (Cornélio *et al*. in preparation). Importantly, these alterations are known to be typical of GCTB^82^. GCTB is a benign neoplasm of mesenchymal origin and is locally aggressive, but may also metastasize (1–2% of cases)^52^. Chromosome 20 is also commonly involved in chromosomal alterations of embryonic stem cells^10,83^. Considering their association with cancer and their location on chromosome 20, higher expression levels of *LAMA5* and *JAG1* in senescent hMSCs/inv suggests that these are more prone to genetic instability, favoring tumorigenesis.

Senescent cells exhibit a senescence-associated secretory phenotype (SASP), a specific secretome including various metalloproteases, extracellular matrix components, adhesion and ligands or receptors molecules, and proinflammatory and chemotactic growth factors, which may contribute to tumor growth [8, 84, 85,86, 87,88, 89, 90] ^8,84–90^. These molecular functions have also been identified for secreted factors of senescent hMSCs derived from other sources^8^. In this study, the DEGs in senescent cells were identified as bottlenecks and hubs, connected into networks enriched in the same functional categories as the SASP constituents related to tumorigenesis such as, *MMP1*, *THBS1*, *KRT19*, *SFRP1*, *FGF1*, *NACM1*, *LAMA5*, *IL11*, *EGF*, *IGFBP3*, *IGFBP4*, *IGFBP5*, *TIMP-3*, and *CXCL12*, as described above. Therefore, these genes may form part of a SASP profile, specifically for senescent hMSCs obtained from the umbilical cord. The imbalance in the expression of these genes may contribute to tumorigenesis, especially for cancers of mesenchymal origin.

## Conclusion

**Figure 7:**
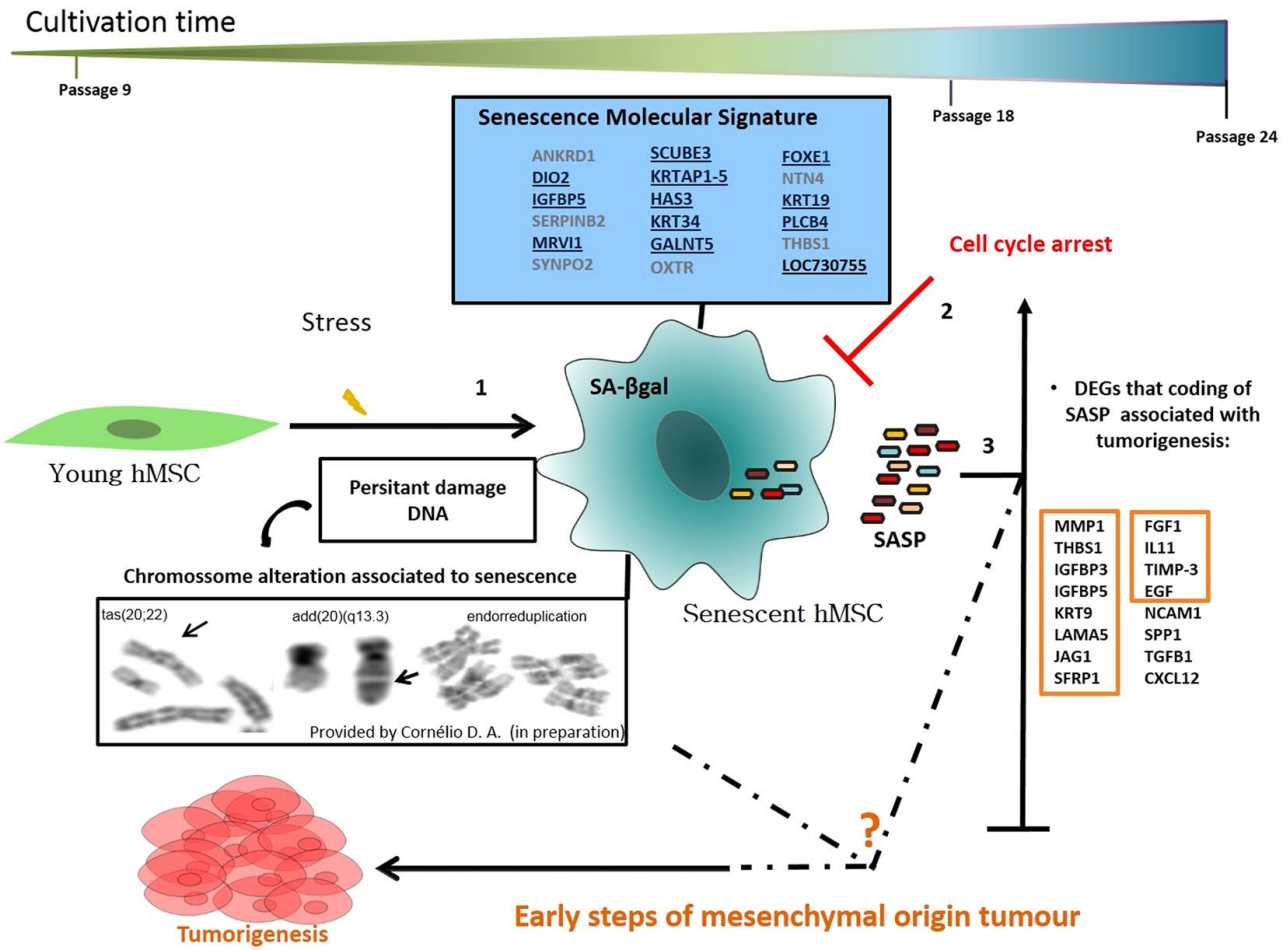
Molecular model of senescence of hMSC and its relation to tumorigenesis processes based on differential gene expression. In the 18th passage there was a predominance of cells exhibiting the phenotypic characteristics of senescence. These cells showed significant changes in gene expression. Of the 18 genes found (box of molecular signature, above left), 11 are new candidates for senescence markers in hMSCs derived from human umbilical cord vein (underlined genes). Throughout the cultivation of these cells, chromosome changes associated with senescence (as endoreduplication) and giant cell tumor of bone (GCTB) (as ‘tas’ and amplification of 20q) were also found predominantly in the cells with constitutional karyotype alterations (hMSC/inv) (provided by Cornélio *et al*. in preparation). Molecules known to be involved in the secretory-associated senescence phenotype (SASP) associated with tumorigenesis were identified mainly in senescent hMSCs/inv (table in the right corner). Four of these molecules are specifically associated with giant cell tumor of bone (GCTB): MMP1, CXCL12, LAMA5, and TGFB1 (underlined in the table in the right corner). The senescent hMSCs/n and hMSCs/inv survived until the 24th passage without any spontaneous transformation. Thus, we hypothesize that cells with this expression profile in hostile microenvironments of tissue injury, inflammation, or stress may generate an imbalance favoring re-entry into the cell cycle and starting tumor formation of mesenchymal origin, such as in GCTB.

This work was the first to undertake a large-scale investigation of differentially expressed genes in hMSCs from the umbilical cord vein and to compare normal and altered constitutional karyotypes between passage 9 and passage 18. The cells could be maintained in culture until approximately the 24th passage without transformation. Expression differences observed between senescent and young hMSCs were greater than senescent cells with altered karyotype (hMSC/inv). We identified 11 new upregulated genes that could potentially be used as senescence markers. The analysis of DEGs comprising specific functional categories in each comparison identified genes involved in cell proliferation, cell differentiation, tumorigenesis, and similar functional categories as the SASP constituents. These findings suggesting new candidate molecules as possible associations between senescence and tumorigenesis in hMSCs (Fig. 7). The new differentially expressed genes involved with tumorigenesis in senescent cells may be useful in monitoring senescence of cultured hMSCs; cells with high expression levels of these genes can be excluded from hMSC therapy. Additional *in vitro* investigations are being carried out in our laboratory with qRT-PCR. Additionally, *in vivo* studies must be performed to investigate the functional relevance of the genes identified in this work to the processes of cellular senescence and tumorigenesis in hMSCs.

## Electronic supplementary material

supplemental file 1
