# Supplementary material for: Identification of new genes associated to senescent and tumorigenic phenotypes in mesenchymal stem cells
Source: Sci Rep. 2017 Dec 19;7:17837. doi: 10.1038/s41598-017-16224-5 (PMC5736717; doi:10.1038/s41598-017-16224-5)
Supplement: Supplementary file 1 — supplemental file 1 [file 41598_2017_16224_MOESM1_ESM.doc]

**Identification of new genes associated to senescent and tumorigenic phenotypes in mesenchymal stem cells.**

Running head: mesenchymal stem cells and senescence

Joana Cristina Medeiros Tavares^1^, Déborah Afonso Cornélio^2^, Vivian Nogueira Silbiger^3^, André Ducati Luchessi^3^, Sandro de Souza^4^, Silvia Regina Batistuzzo de Medeiros^2^

1 Faculdade de Ciências da Saúde do Trairi Faculdade de Ciências da Saúde do Trairi (FACISA), Universidade Federal do Rio Grande do Norte (UFRN), Rua Traíri, S/N, Centro, Santa Cruz, RN, 59200-000, Brazil. E-mail: joanacmt@gmail.com

2 Laboratório de Biologia Molecular e Genômica, Centro de Biociências, UFRN, Avenida Senador Salgado Filho, 3000, Campus Universitário, Lagoa Nova, Natal, RN, 59078-900, Brazil. E-mails: deborahzafonso@gmail.com; sbatistu@cb.ufrn.br

3 Departamento de Análises Clínicas e Toxicológicas, Curso de Farmácia - CCS/UFRN. E-mails: viviansilbiger@hotmail.com; andre.luchessi@outlook.com

4 Brain Institute, UFRN and Bioinformatics Digital Metropolis Institute, UFRN. E-mail: sandro@neuro.ufrn.br

Corresponding author: Silvia Regina Batistuzzo de Medeiros. E-mail: sbatistu@cb.ufrn.br

Tel. # 55.84.3211-9209. Fax # 55.84.3215-3346.

Supplemental File 1. Table of the differentially expressed genes in the senescent hMSC/n compared to young hMSC/n

|  | **Senescent hMSC/n vs Young hMSC/n** |  |  |
| --- | --- | --- | --- |
| **Gene Symbol** | **Gene Title** | **p-value BF*** | **Fold change** |
|  | **Upregulated** |  |  |
| KRTAP1-5 | keratin associated protein 1-5 | 1,50E-08 | 11,57 |
| ANKRD1 | ankyrin repeat domain 1 (cardiac muscle) | 1,71E-05 | 11,22 |
| WFDC1 | WAP four-disulfide core domain 1 | 1,46E-04 | 10,99 |
| PSG5 | pregnancy specific beta-1-glycoprotein 5 | 3,83E-08 | 10,55 |
| LOC730755 | keratin associated protein 2-4-like | 1,73E-10 | 10,35 |
| SCUBE3 | signal peptide, CUB domain, EGF-like 3 | 3,44E-08 | 9,92 |
| FOXE1 | forkhead box E1 (thyroid transcription factor 2) | 4,47E-13 | 9,19 |
| STMN2 | stathmin-like 2 | 7,91E-03 | 8,77 |
| KRT34 | keratin 34 | 5,46E-07 | 8,04 |
| THBS1 | thrombospondin 1 | 2,24E-08 | 7,33 |
| SYNPO2 | synaptopodin 2 | 7,32E-10 | 6,35 |
| MRVI1 | murine retrovirus integration site 1 homolog | 5,08E-09 | 5,88 |
| KCNN2 | potassium intermediate/small conductance calcium-activated channel, subfamily N, | 1,32E-03 | 5,75 |
| SYNPO2L | synaptopodin 2-like | 4,80E-05 | 5,70 |
| HSD17B6 | hydroxysteroid (17-beta) dehydrogenase 6 homolog (mouse) | 3,17E-04 | 5,61 |
| PDGFRL | platelet-derived growth factor receptor-like | 3,41E-11 | 5,59 |
| PLCB4 | phospholipase C, beta 4 | 3,50E-09 | 5,30 |
| HAS3 | hyaluronan synthase 3 | 2,40E-07 | 5,30 |
| SERPINB7 | serpin peptidase inhibitor, clade B (ovalbumin), member 7 | 2,96E-08 | 5,15 |
| OXTR | oxytocin receptor | 3,50E-06 | 5,00 |
| KRT19 | keratin 19 | 8,60E-06 | 4,91 |
| NTN4 | netrin 4 | 1,24E-04 | 4,81 |
| LMCD1 | LIM and cysteine-rich domains 1 | 3,72E-07 | 4,67 |
| GALNT5 | UDP-N-acetyl-alpha-D-galactosamine:polypeptide N-acetylgalactosaminyltransferase | 9,84E-08 | 4,65 |
| SERPINB2 | serpin peptidase inhibitor, clade B (ovalbumin), member 2 | 6,63E-03 | 4,59 |
| KDELR3 | KDEL (Lys-Asp-Glu-Leu) endoplasmic reticulum protein retention receptor 3 | 4,54E-11 | 4,58 |
| IFIT1 | interferon-induced protein with tetratricopeptide repeats 1 | 2,83E-03 | 4,29 |
| SLC1A1 | solute carrier family 1 (neuronal/epithelial high affinity glutamate transporter | 1,37E-08 | 4,28 |
| KIAA1324L | KIAA1324-like | 4,10E-06 | 4,12 |
| PSG6 | pregnancy specific beta-1-glycoprotein 6 | 3,39E-04 | 4,04 |
| DIO2 | deiodinase, iodothyronine, type II | 1,77E-05 | 4,02 |
| PSG9 | pregnancy specific beta-1-glycoprotein 9 | 1,70E-03 | 3,73 |
| ITGBL1 | Integrin, beta-like 1 (with EGF-like repeat domains) | 6,41E-04 | 3,71 |
| IGFBP5 | insulin-like growth factor binding protein 5 | 1,74E-06 | 3,68 |
| TMEM106A | transmembrane protein 106A | 3,57E-06 | 3,62 |
| SYNM | synemin, intermediate filament protein | 1,03E-07 | 3,43 |
| CLIC3 | chloride intracellular channel 3 | 1,15E-03 | 3,33 |
| TUBB2A | tubulin, beta 2A | 1,58E-04 | 3,33 |
| XYLT1 | xylosyltransferase I | 1,21E-03 | 3,32 |
| ANGPT1 | angiopoietin 1 | 3,09E-06 | 3,28 |
| GALNT3 | UDP-N-acetyl-alpha-D-galactosamine:polypeptide N-acetylgalactosaminyltransferase | 3,52E-03 | 3,25 |
| LIMCH1 | LIM and calponin homology domains 1 | 1,06E-05 | 3,24 |
| ENDOD1 | endonuclease domain containing 1 | 7,38E-10 | 3,14 |
| PSG3 | pregnancy specific beta-1-glycoprotein 3 | 2,20E-04 | 3,13 |
| IGFBP3 | insulin-like growth factor binding protein 3 | 2,19E-05 | 3,11 |
| ARRDC4 | arrestin domain containing 4 | 1,06E-04 | 3,09 |
| FABP4 | fatty acid binding protein 4, adipocyte | 7,72E-07 | 3,03 |
|  | **Downregulated** |  |  |
| SLC25A37 | solute carrier family 25, member 37 | 7,71E-03 | -3,03 |
| RBP1 | retinol binding protein 1, cellular | 2,00E-12 | -8,74 |
| PDPN | podoplanin | 2,30E-10 | -8,35 |
| RUNX1T1 | runt-related transcription factor 1; translocated to, 1 (cyclin D-related) | 2,41E-14 | -6,85 |
| SGCG | sarcoglycan, gamma (35kDa dystrophin-associated glycoprotein) | 5,52E-07 | -6,45 |
| PTGFRN | prostaglandin F2 receptor negative regulator | 3,55E-08 | -5,57 |
| PNMA2 | paraneoplastic antigen MA2 | 1,10E-05 | -4,53 |
| MMP1 | matrix metallopeptidase 1 (interstitial collagenase) | 1,96E-03 | -4,34 |
| CADM1 | cell adhesion molecule 1 | 1,09E-07 | -4,30 |
| HOXD10 | homeobox D10 | 1,88E-04 | -4,29 |
| C10orf58 | chromosome 10 open reading frame 58 | 6,05E-05 | -4,08 |
| KCNT2 | potassium channel, subfamily T, member 2 | 2,01E-03 | -3,99 |
| PLXDC2 | plexin domain containing 2 | 9,29E-05 | -3,97 |
| ST6GALNAC3 | ST6 (alpha-N-acetyl-neuraminyl-2,3-beta-galactosyl-1,3)-N-acetylgalactosaminide | 6,86E-05 | -3,91 |
| CPNE8 | copine VIII | 2,24E-06 | -3,89 |
| LOC644242 | hypothetical LOC644242 | 2,60E-03 | -3,55 |
| LOC100127983 | hypothetical protein LOC100127983 | 2,63E-04 | -3,50 |
| LOC100509635 | hypothetical LOC100509635 | 2,15E-07 | -3,48 |
| TNFRSF9 | tumor necrosis factor receptor superfamily, member 9 | 4,73E-03 | -3,46 |
| GPAT2 | glycerol-3-phosphate acyltransferase 2, mitochondrial | 2,07E-05 | -3,44 |
| SLC43A3 | solute carrier family 43, member 3 | 1,25E-03 | -3,41 |
| PHGDH | phosphoglycerate dehydrogenase | 6,31E-03 | -3,40 |
| COL6A2 | collagen, type VI, alpha 2 | 7,41E-08 | -3,37 |
| NFIB | nuclear factor I/B | 1,41E-06 | -3,14 |
| FRAS1 | Fraser syndrome 1 | 3,93E-07 | -3,06 |
| PTPRD | protein tyrosine phosphatase, receptor type, D | 5,87E-06 | -3,05 |

Supplemental File 2. Table of the differentially expressed genes in senescent hMSC/inv compared to young hMSC/inv.

|  | **Senescent hMSC/inv vs young hMSC/inv** | |  |
| --- | --- | --- | --- |
| **Gene Symbol** | **Gene Title** | **p-value BF*** | **Fold change** |
|  | **Upregulated** |  |  |
| NEFM | neurofilament, medium polypeptide | 7,66E-41 | 23,02 |
| LOC730755 | keratin associated protein 2-4-like | 2,21E-35 | 22,94 |
| MAMDC2 | MAM domain containing 2 | 2,98E-32 | 17,11 |
| DIO2 | deiodinase, iodothyronine, type II | 9,43E-31 | 15,25 |
| KISS1 | KiSS-1 metastasis-suppressor | 0 | 11,48 |
| IGFBP5 | insulin-like growth factor binding protein 5 | 6,41E-37 | 11,27 |
| SERPINB2 | serpin peptidase inhibitor, clade B (ovalbumin), member 2 | 3,07E-20 | 10,07 |
| THBS1 | thrombospondin 1 | 4,76E-34 | 9,88 |
| EHF | ets homologous factor | 7,63E-13 | 9,66 |
| F3 | coagulation factor III (thromboplastin, tissue factor) | 0 | 9,53 |
| SCN3A | sodium channel, voltage-gated, type III, alpha subunit | 4,71E-18 | 8,83 |
| L1CAM | L1 cell adhesion molecule | 1,53E-40 | 8,75 |
| EDN1 | endothelin 1 | 8,04E-39 | 8,46 |
| OLR1 | oxidized low density lipoprotein (lectin-like) receptor 1 | 3,63E-33 | 8,23 |
| MYOCD | myocardin | 2,13E-35 | 7,74 |
| RGS5 | regulator of G-protein signaling 5 | 5,86E-36 | 7,70 |
| MRVI1 | murine retrovirus integration site 1 homolog | 1,55E-28 | 7,70 |
| SYNPO2 | synaptopodin 2 | 1,71E-28 | 7,70 |
| ZPLD1 | zona pellucida-like domain containing 1 | 1,59E-30 | 7,42 |
| PLN | phospholamban | 1,27E-22 | 7,35 |
| NRK | Nik related kinase | 0 | 7,20 |
| AK4 | adenylate kinase 4 | 5,90E-39 | 7,00 |
| EGF | epidermal growth factor | 4,39E-28 | 6,96 |
| NPPB | natriuretic peptide B | 1,90E-25 | 6,82 |
| CHRM3 | cholinergic receptor, muscarinic 3 | 4,70E-34 | 6,75 |
| KRTAP1-5 | keratin associated protein 1-5 | 6,81E-21 | 6,68 |
| DNAJC6 | DnaJ (Hsp40) homolog, subfamily C, member 6 | 2,64E-34 | 6,65 |
| BEX1 | brain expressed, X-linked 1 | 5,90E-39 | 6,58 |
| FOXE1 | forkhead box E1 (thyroid transcription factor 2) | 9,41E-28 | 6,28 |
| SCD5 | stearoyl-CoA desaturase 5 | 3,67E-38 | 6,08 |
| NRG1 | neuregulin 1 | 4,32E-17 | 5,70 |
| GPRC5A | G protein-coupled receptor, family C, group 5, member A | 4,81E-38 | 5,32 |
| NTN4 | netrin 4 | 4,13E-19 | 5,29 |
| SGIP1 | SH3-domain GRB2-like (endophilin) interacting protein 1 | 1,21E-17 | 5,15 |
| CCDC81 | coiled-coil domain containing 81 | 6,26E-22 | 4,94 |
| KCTD4 | potassium channel tetramerisation domain containing 4 | 3,12E-29 | 4,90 |
| RGS4 | regulator of G-protein signaling 4 | 4,11E-22 | 4,86 |
| C15orf54 | chromosome 15 open reading frame 54 | 6,76E-35 | 4,86 |
| PLAT | plasminogen activator, tissue | 1,30E-27 | 4,85 |
| EREG | epiregulin | 7,44E-19 | 4,84 |
| FHL1 | four and a half LIM domains 1 | 8,06E-18 | 4,84 |
| GRP | gastrin-releasing peptide | 1,10E-19 | 4,76 |
| TMEM171 | transmembrane protein 171 | 2,10E-21 | 4,74 |
| LMOD1 | leiomodin 1 (smooth muscle) | 6,26E-22 | 4,69 |
| TM4SF1 | transmembrane 4 L six family member 1 | 4,55E-25 | 4,69 |
| DLG1 | Discs, large homolog 1 (Drosophila) | 2,83E-13 | 4,68 |
| SH3RF2 | SH3 domain containing ring finger 2 | 1,00E-27 | 4,62 |
| HIST1H2BC | histone cluster 1, H2bc | 2,07E-22 | 4,62 |
| NOG | noggin | 1,29E-09 | 4,59 |
| CDH8 | cadherin 8, type 2 | 1,93E-26 | 4,53 |
| RGMB | RGM domain family, member B | 6,04E-21 | 4,50 |
| SERPIND1 | serpin peptidase inhibitor, clade D (heparin cofactor), member 1 | 2,01E-09 | 4,50 |
| ANKRD1 | ankyrin repeat domain 1 (cardiac muscle) | 6,78E-10 | 4,46 |
| HBEGF | heparin-binding EGF-like growth factor | 2,36E-25 | 4,45 |
| TRPC4 | transient receptor potential cation channel, subfamily C, member 4 | 5,17E-21 | 4,41 |
| HHIP | hedgehog interacting protein | 5,11E-27 | 4,38 |
| NEFL | neurofilament, light polypeptide | 1,21E-18 | 4,37 |
| PRICKLE1 | prickle homolog 1 (Drosophila) | 9,30E-28 | 4,32 |
| CXADR | coxsackie virus and adenovirus receptor | 1,68E-29 | 4,30 |
| OXTR | oxytocin receptor | 6,67E-13 | 4,29 |
| TXNIP | thioredoxin interacting protein | 5,99E-16 | 4,27 |
| SERPINE1 | serpin peptidase inhibitor, clade E (nexin, plasminogen activator inhibitor type | 2,36E-10 | 4,25 |
| KCTD20 | potassium channel tetramerisation domain containing 20 | 4,77E-37 | 4,21 |
| ATP2B4 | ATPase, Ca++ transporting, plasma membrane 4 | 4,40E-35 | 4,17 |
| SFRP1 | secreted frizzled-related protein 1 | 1,44E-25 | 4,16 |
| EPPK1 | epiplakin 1 | 3,23E-23 | 4,11 |
| WNT16 | wingless-type MMTV integration site family, member 16 | 5,04E-23 | 4,10 |
| KRT19 | keratin 19 | 3,92E-24 | 4,04 |
| CACNA1A | calcium channel, voltage-dependent, P/Q type, alpha 1A subunit | 8,06E-33 | 3,94 |
| PLCB4 | phospholipase C, beta 4 | 7,57E-18 | 3,91 |
| CNTNAP3 | contactin associated protein-like 3 | 1,75E-26 | 3,83 |
| SCUBE3 | signal peptide, CUB domain, EGF-like 3 | 1,11E-11 | 3,82 |
| AGTR1 | angiotensin II receptor, type 1 | 1,26E-24 | 3,80 |
| CD274 | CD274 molecule | 7,42E-14 | 3,77 |
| CALD1 | caldesmon 1 | 6,60E-15 | 3,75 |
| HAS3 | hyaluronan synthase 3 | 3,05E-12 | 3,74 |
| TIMP3 | TIMP metallopeptidase inhibitor 3 | 5,81E-30 | 3,68 |
| KRT34 | keratin 34 | 7,17E-10 | 3,66 |
| TSPAN2 | tetraspanin 2 | 1,77E-14 | 3,65 |
| ARHGAP29 | Rho GTPase activating protein 29 | 9,67E-25 | 3,57 |
| PDCD1LG2 | programmed cell death 1 ligand 2 | 1,12E-22 | 3,54 |
| SLC7A2 | solute carrier family 7 (cationic amino acid transporter, y+ system), member 2 | 4,76E-13 | 3,51 |
| OBFC2A | oligonucleotide/oligosaccharide-binding fold containing 2A | 8,50E-20 | 3,51 |
| SHROOM3 | shroom family member 3 | 2,98E-25 | 3,49 |
| LOC401093 | hypothetical LOC401093 | 1,36E-18 | 3,49 |
| NRXN3 | neurexin 3 | 1,91E-21 | 3,48 |
| ARSJ | arylsulfatase family, member J | 6,49E-31 | 3,48 |
| ATP8A1 | ATPase, aminophospholipid transporter (APLT), class I, type 8A, member 1 | 2,65E-28 | 3,40 |
| CCND2 | cyclin D2 | 1,61E-22 | 3,38 |
| DAB2 | disabled homolog 2, mitogen-responsive phosphoprotein (Drosophila) | 9,94E-11 | 3,37 |
| SUSD2 | sushi domain containing 2 | 3,50E-22 | 3,36 |
| HIST1H2BG | histone cluster 1, H2bg | 4,29E-18 | 3,30 |
| CDH10 | cadherin 10, type 2 (T2-cadherin) | 9,30E-23 | 3,26 |
| HIST1H2BD | histone cluster 1, H2bd | 9,50E-23 | 3,25 |
| CFLAR | CASP8 and FADD-like apoptosis regulator | 8,59E-33 | 3,24 |
| SPOCD1 | SPOC domain containing 1 | 7,28E-24 | 3,23 |
| DUSP5 | dual specificity phosphatase 5 | 4,16E-16 | 3,22 |
| FGF1 | fibroblast growth factor 1 (acidic) | 5,90E-19 | 3,20 |
| ARID5B | AT rich interactive domain 5B (MRF1-like) | 1,98E-14 | 3,20 |
| WNT5B | wingless-type MMTV integration site family, member 5B | 2,63E-28 | 3,18 |
| ACTB | actin, beta | 1,99E-07 | 3,17 |
| GALNT5 | UDP-N-acetyl-alpha-D-galactosamine:polypeptide N-acetylgalactosaminyltransferase | 2,10E-14 | 3,16 |
| SLC16A3 | solute carrier family 16, member 3 (monocarboxylic acid transporter 4) | 1,10E-12 | 3,16 |
| SOCS2 | suppressor of cytokine signaling 2 | 6,40E-16 | 3,16 |
| HIST2H2AA3 /// HIST2H2AA4 | histone cluster 2, H2aa3 /// histone cluster 2, H2aa4 | 8,80E-19 | 3,16 |
| JAM2 | junctional adhesion molecule 2 | 2,12E-17 | 3,15 |
| COL8A1 | collagen, type VIII, alpha 1 | 2,69E-10 | 3,15 |
| STYK1 | serine/threonine/tyrosine kinase 1 | 2,50E-13 | 3,15 |
| JPH2 | junctophilin 2 | 3,12E-18 | 3,15 |
| TNFRSF10D | tumor necrosis factor receptor superfamily, member 10d, decoy with truncated dea | 2,38E-04 | 3,15 |
| SLC2A1 | solute carrier family 2 (facilitated glucose transporter), member 1 | 2,22E-28 | 3,13 |
| ENO1 | enolase 1, (alpha) | 6,83E-07 | 3,13 |
| CDCP1 | CUB domain containing protein 1 | 1,34E-12 | 3,12 |
| NEAT1 | nuclear paraspeckle assembly transcript 1 (non-protein coding) | 9,25E-13 | 3,10 |
| PHKA1 | phosphorylase kinase, alpha 1 (muscle) | 5,29E-21 | 3,09 |
| FMN2 | formin 2 | 6,37E-21 | 3,04 |
| PICALM | phosphatidylinositol binding clathrin assembly protein | 2,43E-10 | 3,03 |
| NEDD4L | neural precursor cell expressed, developmentally down-regulated 4-like | 3,29E-27 | 3,01 |
| GPR155 | G protein-coupled receptor 155 | 4,13E-14 | 3,01 |
|  | **Downregulated** |  |  |
| SGCG | sarcoglycan, gamma (35kDa dystrophin-associated glycoprotein) | 8,66E-39 | -20,14 |
| PTGIS | prostaglandin I2 (prostacyclin) synthase | 3,06E-40 | 19,49 |
| DOK5 | docking protein 5 | 0 | -17,62 |
| RBP1 | retinol binding protein 1, cellular | 7,47E-38 | -15,17 |
| TNFAIP6 | tumor necrosis factor, alpha-induced protein 6 | 2,50E-30 | -14,13 |
| SLC22A3 | solute carrier family 22 (extraneuronal monoamine transporter), member 3 | 7,68E-26 | -13,02 |
| E2F7 | E2F transcription factor 7 | 2,06E-28 | -12,42 |
| KLF4 | Kruppel-like factor 4 (gut) | 3,27E-20 | -12,35 |
| LAMP3 | lysosomal-associated membrane protein 3 | 1,13E-12 | -12,28 |
| NUPR1 | nuclear protein, transcriptional regulator, 1 | 1,33E-20 | -11,54 |
| LOC100132891 | hypothetical LOC100132891 | 7,31E-34 | -11,26 |
| HGF | hepatocyte growth factor (hepapoietin A; scatter factor) | 2,76E-33 | -10,56 |
| EFEMP1 | EGF-containing fibulin-like extracellular matrix protein 1 | 1,05E-21 | -10,15 |
| RUNX1T1 | runt-related transcription factor 1; translocated to, 1 (cyclin D-related) | 3,10E-27 | -9,20 |
| GXYLT2 | glucoside xylosyltransferase 2 | 2,28E-32 | -8,79 |
| RUNX2 | runt-related transcription factor 2 | 1,29E-33 | -8,39 |
| P4HA3 | prolyl 4-hydroxylase, alpha polypeptide III | 3,75E-35 | -8,18 |
| LOC401097 | hypothetical protein LOC401097 | 1,70E-21 | -7,84 |
| SLITRK4 | SLIT and NTRK-like family, member 4 | 2,26E-24 | -7,53 |
| FAM129A | family with sequence similarity 129, member A | 2,23E-15 | -7,46 |
| HTR2A | 5-hydroxytryptamine (serotonin) receptor 2A | 2,38E-35 | -7,10 |
| SCRG1 | stimulator of chondrogenesis 1 | 1,19E-18 | -7,08 |
| DDIT4 | DNA-damage-inducible transcript 4 | 8,96E-05 | -6,93 |
| TDO2 | tryptophan 2,3-dioxygenase | 1,73E-26 | -6,93 |
| FBXO32 | F-box protein 32 | 6,82E-17 | -6,75 |
| PTPRG | protein tyrosine phosphatase, receptor type, G | 1,69E-33 | -6,54 |
| GPC6 | glypican 6 | 2,96E-30 | -6,39 |
| ASNS | asparagine synthetase (glutamine-hydrolyzing) | 2,99E-08 | -6,38 |
| G0S2 | G0/G1switch 2 | 3,45E-19 | -6,37 |
| DCLK1 | doublecortin-like kinase 1 | 3,47E-22 | -6,30 |
| SULT1E1 | sulfotransferase family 1E, estrogen-preferring, member 1 | 1,55E-33 | -5,81 |
| C1R | complement component 1, r subcomponent | 1,28E-24 | -5,71 |
| INHBE | inhibin, beta E | 1,19E-06 | -5,64 |
| LOC100505633 | hypothetical LOC100505633 | 2,08E-22 | -5,52 |
| BEX2 | brain expressed X-linked 2 | 4,59E-10 | -5,48 |
| HMOX1 | heme oxygenase (decycling) 1 | 7,45E-16 | -5,37 |
| MN1 | meningioma (disrupted in balanced translocation) 1 | 2,44E-31 | -5,33 |
| C4orf7 | chromosome 4 open reading frame 7 | 4,66E-34 | -5,31 |
| PHGDH | phosphoglycerate dehydrogenase | 1,52E-17 | -5,30 |
| NDN | necdin homolog (mouse) | 1,76E-39 | -5,27 |
| CTH | cystathionase (cystathionine gamma-lyase) | 4,95E-10 | -5,11 |
| HSPBAP1 | HSPB (heat shock 27kDa) associated protein 1 | 4,68E-17 | -5,06 |
| AFF3 | AF4/FMR2 family, member 3 | 1,38E-22 | -5,00 |
| ABCA8 | ATP-binding cassette, sub-family A (ABC1), member 8 | 2,04E-29 | -4,94 |
| MOCOS | molybdenum cofactor sulfurase | 2,04E-16 | -4,91 |
| GDF15 | growth differentiation factor 15 | 4,78E-06 | -4,84 |
| VDR | vitamin D (1,25- dihydroxyvitamin D3) receptor | 2,03E-23 | -4,84 |
| CARD16 | caspase recruitment domain family, member 16 | 2,04E-29 | -4,76 |
| EBF1 | early B-cell factor 1 | 4,70E-27 | -4,76 |
| GALNT12 | UDP-N-acetyl-alpha-D-galactosamine:polypeptide N-acetylgalactosaminyltransferase | 2,29E-26 | -4,73 |
| SLC14A1 | solute carrier family 14 (urea transporter), member 1 (Kidd blood group) | 3,24E-26 | -4,65 |
| GAS2L3 | Growth arrest-specific 2 like 3 | 1,04E-23 | -4,61 |
| ANGPTL1 | angiopoietin-like 1 | 2,51E-12 | -4,50 |
| ST3GAL6 | ST3 beta-galactoside alpha-2,3-sialyltransferase 6 | 1,41E-19 | -4,42 |
| TRIB3 | tribbles homolog 3 (Drosophila) | 1,48E-05 | -4,40 |
| UNC5B | unc-5 homolog B (C. elegans) | 6,97E-20 | -4,34 |
| ALDH1L2 | aldehyde dehydrogenase 1 family, member L2 | 3,90E-16 | -4,32 |
| ANK2 | ankyrin 2, neuronal | 2,34E-09 | -4,30 |
| DNAJC12 | DnaJ (Hsp40) homolog, subfamily C, member 12 | 4,80E-26 | -4,30 |
| GPAT2 | glycerol-3-phosphate acyltransferase 2, mitochondrial | 3,23E-25 | -4,23 |
| LOC100127983 | hypothetical protein LOC100127983 | 3,22E-21 | -4,22 |
| TSLP | thymic stromal lymphopoietin | 7,76E-16 | -4,21 |
| GEM | GTP binding protein overexpressed in skeletal muscle | 2,47E-22 | -4,20 |
| DIRAS3 | DIRAS family, GTP-binding RAS-like 3 | 5,56E-13 | -4,19 |
| MXRA5 | matrix-remodelling associated 5 | 1,99E-11 | -4,12 |
| ST6GAL2 | ST6 beta-galactosamide alpha-2,6-sialyltranferase 2 | 6,78E-22 | -4,09 |
| FRMD5 | FERM domain containing 5 | 4,27E-19 | -4,04 |
| BACE2 | beta-site APP-cleaving enzyme 2 | 9,51E-32 | -4,04 |
| TNFRSF9 | tumor necrosis factor receptor superfamily, member 9 | 1,57E-18 | -4,03 |
| COL15A1 | collagen, type XV, alpha 1 | 3,91E-28 | -4,02 |
| KRT7 | keratin 7 | 1,92E-26 | -4,01 |
| CREG1 | cellular repressor of E1A-stimulated genes 1 | 5,75E-24 | -4,01 |
| CCDC8 | coiled-coil domain containing 8 | 1,35E-32 | -3,99 |
| PCK2 | phosphoenolpyruvate carboxykinase 2 (mitochondrial) | 6,98E-07 | -3,98 |
| LOC100507248 | hypothetical LOC100507248 | 4,18E-20 | -3,93 |
| SLC12A8 | solute carrier family 12 (potassium/chloride transporters), member 8 | 2,64E-19 | -3,91 |
| PDZRN3 | PDZ domain containing ring finger 3 | 1,08E-14 | -3,89 |
| SULF2 | sulfatase 2 | 2,03E-25 | -3,88 |
| KAL1 | Kallmann syndrome 1 sequence | 2,16E-17 | -3,87 |
| PDE4DIP | phosphodiesterase 4D interacting protein | 2,89E-28 | -3,85 |
| PTER | phosphotriesterase related | 3,86E-20 | -3,84 |
| ENPP1 | ectonucleotide pyrophosphatase/phosphodiesterase 1 | 3,62E-26 | -3,84 |
| RAB27B | RAB27B, member RAS oncogene family | 3,70E-15 | -3,82 |
| ARL4C | ADP-ribosylation factor-like 4C | 4,25E-11 | -3,80 |
| BHLHE40 | basic helix-loop-helix family, member e40 | 6,78E-15 | -3,77 |
| CPEB1 | cytoplasmic polyadenylation element binding protein 1 | 2,75E-14 | -3,77 |
| PLXDC2 | plexin domain containing 2 | 7,51E-25 | -3,73 |
| CHI3L1 | chitinase 3-like 1 (cartilage glycoprotein-39) | 7,97E-03 | -3,70 |
| EPDR1 | ependymin related protein 1 (zebrafish) | 5,95E-27 | -3,69 |
| CASP1 | caspase 1, apoptosis-related cysteine peptidase (interleukin 1, beta, convertase | 2,31E-23 | -3,67 |
| ETV1 | ets variant 1 | 4,73E-27 | -3,66 |
| HEPH | hephaestin | 6,99E-29 | -3,65 |
| CYP1B1 | cytochrome P450, family 1, subfamily B, polypeptide 1 | 3,63E-10 | -3,62 |
| FAM84B | family with sequence similarity 84, member B | 2,40E-10 | -3,60 |
| F2RL1 | coagulation factor II (thrombin) receptor-like 1 | 2,55E-09 | -3,59 |
| GOLM1 | golgi membrane protein 1 | 0 | -3,59 |
| CRISPLD1 | cysteine-rich secretory protein LCCL domain containing 1 | 5,46E-21 | -3,58 |
| SNCAIP | synuclein, alpha interacting protein | 9,30E-26 | -3,57 |
| DOCK11 | dedicator of cytokinesis 11 | 9,18E-33 | -3,56 |
| PTGFRN | prostaglandin F2 receptor negative regulator | 5,28E-19 | -3,55 |
| GAS1 | growth arrest-specific 1 | 1,39E-14 | -3,51 |
| IL33 | interleukin 33 | 1,18E-05 | -3,49 |
| ST6GAL1 | ST6 beta-galactosamide alpha-2,6-sialyltranferase 1 | 9,24E-14 | -3,49 |
| PSAT1 | phosphoserine aminotransferase 1 | 4,48E-04 | -3,48 |
| OR51E2 | olfactory receptor, family 51, subfamily E, member 2 | 3,02E-27 | -3,47 |
| CXCL12 | chemokine (C-X-C motif) ligand 12 | 4,69E-14 | -3,47 |
| FST | follistatin | 9,70E-29 | -3,47 |
| LOC100127983 | hypothetical protein LOC100127983 | 3,32E-23 | -3,45 |
| LAMA1 | laminin, alpha 1 | 7,99E-14 | -3,43 |
| C13orf33 | chromosome 13 open reading frame 33 | 4,92E-19 | -3,41 |
| C1S | complement component 1, s subcomponent | 6,45E-15 | -3,39 |
| AKNA | AT-hook transcription factor | 1,21E-21 | -3,36 |
| PID1 | phosphotyrosine interaction domain containing 1 | 6,66E-29 | -3,35 |
| WARS | tryptophanyl-tRNA synthetase | 4,09E-15 | -3,33 |
| MMP2 | matrix metallopeptidase 2 (gelatinase A, 72kDa gelatinase, 72kDa type IV collage | 7,65E-21 | -3,33 |
| ZNF521 | zinc finger protein 521 | 3,87E-21 | -3,33 |
| RPS6KA2 | ribosomal protein S6 kinase, 90kDa, polypeptide 2 | 3,73E-26 | -3,33 |
| C11orf75 | chromosome 11 open reading frame 75 | 5,39E-23 | -3,30 |
| PBX1 | pre-B-cell leukemia homeobox 1 | 7,21E-20 | -3,29 |
| SH3KBP1 | SH3-domain kinase binding protein 1 | 1,49E-35 | -3,29 |
| PTPRE | protein tyrosine phosphatase, receptor type, E | 1,19E-16 | -3,29 |
| ZNF711 | zinc finger protein 711 | 6,39E-22 | -3,28 |
| USP54 | ubiquitin specific peptidase 54 | 1,03E-21 | -3,28 |
| ANXA10 | annexin A10 | 8,41E-19 | -3,27 |
| NRP1 | neuropilin 1 | 4,35E-30 | -3,27 |
| COL6A3 | collagen, type VI, alpha 3 | 1,79E-26 | -3,26 |
| NMI | N-myc (and STAT) interactor | 1,05E-25 | -3,24 |
| CDKN1C | cyclin-dependent kinase inhibitor 1C (p57, Kip2) | 2,78E-16 | -3,22 |
| MMP16 | matrix metallopeptidase 16 (membrane-inserted) | 1,65E-24 | -3,21 |
| ARHGEF6 | Rac/Cdc42 guanine nucleotide exchange factor (GEF) 6 | 3,12E-22 | -3,21 |
| C3orf55 | chromosome 3 open reading frame 55 | 5,93E-25 | -3,21 |
| KLHL24 | kelch-like 24 (Drosophila) | 8,40E-07 | -3,20 |
| LONRF1 | LON peptidase N-terminal domain and ring finger 1 | 4,75E-15 | -3,19 |
| C9orf91 | chromosome 9 open reading frame 91 | 2,21E-10 | -3,19 |
| MCTP1 | multiple C2 domains, transmembrane 1 | 8,00E-08 | -3,18 |
| ATP2B1 | ATPase, Ca++ transporting, plasma membrane 1 | 2,92E-18 | -3,17 |
| GPNMB | glycoprotein (transmembrane) nmb | 5,88E-07 | -3,17 |
| SESN2 | sestrin 2 | 1,12E-04 | -3,15 |
| ADAM12 | ADAM metallopeptidase domain 12 | 1,22E-16 | -3,15 |
| RHOU | ras homolog gene family, member U | 2,01E-21 | -3,13 |
| PTPRD | protein tyrosine phosphatase, receptor type, D | 3,28E-22 | -3,12 |
| IRAK1 | interleukin-1 receptor-associated kinase 1 | 1,32E-32 | -3,12 |
| ST8SIA4 | ST8 alpha-N-acetyl-neuraminide alpha-2,8-sialyltransferase 4 | 5,11E-15 | -3,10 |
| RGS17 | regulator of G-protein signaling 17 | 2,35E-16 | -3,09 |
| PAG1 | phosphoprotein associated with glycosphingolipid microdomains 1 | 7,32E-15 | -3,06 |
| FBLN2 | fibulin 2 | 8,84E-28 | -3,06 |
| GUCY1B3 | guanylate cyclase 1, soluble, beta 3 | 7,15E-20 | -3,05 |
| DDIT3 | DNA-damage-inducible transcript 3 | 4,42E-05 | -3,05 |
| C3orf14 | chromosome 3 open reading frame 14 | 2,74E-28 | -3,04 |
| MFAP3L | microfibrillar-associated protein 3-like | 1,58E-18 | -3,04 |
| FAM38B | family with sequence similarity 38, member B | 5,84E-07 | -3,04 |
| ST6GALNAC3 | ST6 (alpha-N-acetyl-neuraminyl-2,3-beta-galactosyl-1,3)-N-acetylgalactosaminide | 2,83E-11 | -3,04 |
| LTBP2 | latent transforming growth factor beta binding protein 2 | 9,66E-24 | -3,03 |
| ZNF618 | zinc finger protein 618 | 2,12E-23 | -3,02 |
| EIF4EBP1 | eukaryotic translation initiation factor 4E binding protein 1 | 4,51E-13 | -3,02 |
| HMGB3 | high-mobility group box 3 | 1,49E-28 | -3,01 |
| CBLB | Cas-Br-M (murine) ecotropic retroviral transforming sequence b | 7,55E-15 | -3,01 |
| STMN2 | stathmin-like 2 | 3,26E-07 | -3,00 |
| PABPC4L | poly(A) binding protein, cytoplasmic 4-like | 9,22E-14 | -3,00 |
| LRRC49 | leucine rich repeat containing 49 | 2,20E-20 | -3,00 |
|  |  |  |  |

| **Gene Symbol**  Supplental File 3. Table with the 30 commom differentially expressed genes in senescent compared to young hMSCs | **Gene Title** | **p-value BF*** | **Fold change** | **p-value BF*** | **Fold change** |
| --- | --- | --- | --- | --- | --- |
| **Upregulated** | | | | | |
| LOC730755 | keratin associated protein 2-4-like | 1,73E-10 | 10,3481 | 2,21E-35 | 22,9422 |
| DIO2 | deiodinase, iodothyronine, type II | 1,77E-05 | 4,01565 | 9,43E-31 | 15,2498 |
| IGFBP5 | insulin-like growth factor binding protein 5 | 1,74E-06 | 3,68274 | 6,73E-36 | 10,9562 |
| SERPINB2 | serpin peptidase inhibitor, clade B (ovalbumin), member 2 | 6,63E-03 | 4,58767 | 3,07E-20 | 10,0713 |
| SYNPO2 | synaptopodin 2 | 7,32E-10 | 6,32557 | 1,71E-28 | 8,45205 |
| KRTAP1-5 | keratin associated protein 1-5 | 1,50E-08 | 11,5727 | 6,81E-21 | 6,68396 |
| THBS1 | thrombospondin 1 | 2,24E-08 | 4,21229 | 1,43E-24 | 9,87851 |
| FOXE1 | forkhead box E1 (thyroid transcription factor 2) | 4,47E-13 | 9,1907 | 9,41E-28 | 6,2831 |
| NTN4 | netrin 4 | 1,24E-04 | 4,80584 | 4,13E-19 | 5,29025 |
| MRVI1 | murine retrovirus integration site 1 homolog | 5,08E-09 | 5,88388 | 4,91E-21 | 5,06818 |
| ANKRD1 | ankyrin repeat domain 1 (cardiac muscle) | 1,71E-05 | 11,2205 | 6,78E-10 | 4,46141 |
| OXTR | oxytocin receptor | 3,50E-06 | 5,00036 | 6,67E-13 | 4,29455 |
| KRT19 | keratin 19 | 8,60E-06 | 4,91016 | 3,92E-24 | 4,04376 |
| PLCB4 | phospholipase C, beta 4 | 3,50E-09 | 5,30488 | 7,57E-18 | 3,90501 |
| SCUBE3 | signal peptide, CUB domain, EGF-like 3 | 3,44E-08 | 9,91539 | 1,11E-11 | 3,82379 |
| HAS3 | hyaluronan synthase 3 | 2,40E-07 | 5,30118 | 3,05E-12 | 3,74271 |
| KRT34 | keratin 34 | 5,46E-07 | 8,03955 | 7,17E-10 | 3,66283 |
| GALNT5 | UDP-N-acetyl-alpha-D-galactosamine:polypeptide N-acetylgalactosaminyltransferase | 9,84E-08 | 4,64772 | 2,10E-14 | 3,16061 |
| STMN2 | stathmin-like 2 | 7,91E-03 | 8,77335 | 3,26E-07 | -3,0024 |
| **Downregulated** | | | | | |
| SGCG | sarcoglycan, gamma (35kDa dystrophin-associated glycoprotein) | 5,52E-07 | -6,44601 | 8,66E-39 | -20,1389 |
| RBP1 | retinol binding protein 1, cellular | 2,00E-12 | -8,74215 | 7,47E-38 | -15,1729 |
| PHGDH | phosphoglycerate dehydrogenase | 0,00630747 | -3,40163 | 1,52E-17 | -5,29944 |
| GPAT2 | glycerol-3-phosphate acyltransferase 2, mitochondrial | 2,07E-05 | -3,44409 | 3,23E-25 | -4,23125 |
| TNFRSF9 | tumor necrosis factor receptor superfamily, member 9 | 0,00473343 | -3,45978 | 1,57E-18 | -4,0285 |
| RUNX1T1 | runt-related transcription factor 1; translocated to, 1 (cyclin D-related) | 2,41E-14 | -6,8492 | 2,58E-21 | -3,81081 |
| PLXDC2 | plexin domain containing 2 | 9,29E-05 | -3,96652 | 7,51E-25 | -3,72513 |
| PTGFRN | prostaglandin F2 receptor negative regulator | 3,55E-08 | -5,57376 | 5,28E-19 | -3,54801 |
| LOC100127983 | hypothetical protein LOC100127983 | 0,000263312 | -3,5043 | 3,32E-23 | -3,45239 |
| PTPRD | protein tyrosine phosphatase, receptor type, D | 5,87E-06 | -3,04996 | 3,28E-22 | -3,12189 |
| ST6GALNAC3 | ST6 (alpha-N-acetyl-neuraminyl-2,3-beta-galactosyl-1,3)-N-acetylgalactosaminide | 6,86E-05 | -3,9113 | 2,83E-11 | -3,04102 |

| Supplemental File 4. Table of differentially expressed genes in youg hMSC/inv compared to young hMSC/n. | **Young hMSC/inv vs Young hMSC/n** |  |  |
| --- | --- | --- | --- |
|  | **Upregulated** |  |  |
| **Gene Symbol** | **Gene Title** | **p-value BF*** | **Fold Change** |
| ALDH1A1 | aldehyde dehydrogenase 1 family, member A1 | 2,30E-21 | 26,1744 |
| SFRP1 | secreted frizzled-related protein 1 | 2,00E-29 | 19,1773 |
| LAMC2 | laminin, gamma 2 | 5,89E-32 | 14,4033 |
| HLA-DPA1 | major histocompatibility complex, class II, DP alpha 1 | 9,12E-27 | 10,2371 |
| MMP1 | matrix metallopeptidase 1 (interstitial collagenase) | 7,99E-11 | 8,79661 |
| ANKRD1 | ankyrin repeat domain 1 (cardiac muscle) | 1,07E-08 | 8,77528 |
| C4orf7 | chromosome 4 open reading frame 7 | 1,72E-25 | 7,86496 |
| EFEMP1 | EGF-containing fibulin-like extracellular matrix protein 1 | 2,12E-14 | 7,53702 |
| MFAP5 | microfibrillar associated protein 5 | 4,21E-17 | 7,38419 |
| NTN4 | netrin 4 | 5,43E-13 | 7,1124 |
| FOXE1 | forkhead box E1 (thyroid transcription factor 2) | 3,14E-18 | 7,04331 |
| CCL20 | chemokine (C-C motif) ligand 20 | 2,06E-08 | 7,0294 |
| HTR2A | 5-hydroxytryptamine (serotonin) receptor 2A | 3,74E-21 | 6,86942 |
| DUSP4 | dual specificity phosphatase 4 | 2,46E-14 | 6,69553 |
| SERTAD4 | SERTA domain containing 4 | 1,32E-19 | 6,07955 |
| HHIP | hedgehog interacting protein | 1,63E-18 | 5,93659 |
| GALNT3 | UDP-N-acetyl-alpha-D-galactosamine:polypeptide N-acetylgalactosaminyltransferase | 1,51E-12 | 5,80196 |
| PCOLCE2 | procollagen C-endopeptidase enhancer 2 | 2,39E-20 | 5,46642 |
| RPS4Y1 | ribosomal protein S4, Y-linked 1 | 1,31E-31 | 5,461 |
| LOC730755 | keratin associated protein 2-4-like | 1,41E-11 | 5,25427 |
| AP1S3 | adaptor-related protein complex 1, sigma 3 subunit | 4,72E-19 | 4,85569 |
| HOXC10 | homeobox C10 | 1,81E-15 | 4,75863 |
| CNTN1 | Contactin 1 | 1,94E-14 | 4,73638 |
| CXCL12 | chemokine (C-X-C motif) ligand 12 | 1,01E-09 | 4,50266 |
| DDX3Y | DEAD (Asp-Glu-Ala-Asp) box polypeptide 3, Y-linked | 2,22E-25 | 4,41384 |
| FABP4 | fatty acid binding protein 4, adipocyte | 9,37E-16 | 4,3772 |
| SCUBE3 | signal peptide, CUB domain, EGF-like 3 | 4,89E-07 | 4,3245 |
| C1orf133 | chromosome 1 open reading frame 133 | 7,49E-20 | 4,30805 |
| NLGN4Y | neuroligin 4, Y-linked | 2,20E-17 | 4,25777 |
| TNFRSF21 | tumor necrosis factor receptor superfamily, member 21 | 6,51E-16 | 4,18942 |
| NCAM1 | neural cell adhesion molecule 1 | 2,35E-19 | 4,13071 |
| EIF1AY | eukaryotic translation initiation factor 1A, Y-linked | 3,11E-26 | 4,05634 |
| CNIH3 | cornichon homolog 3 (Drosophila) | 1,78E-07 | 4,04554 |
| LOC644246 | hypothetical LOC644246 | 7,50E-15 | 4,02313 |
| SLITRK4 | SLIT and NTRK-like family, member 4 | 3,30E-07 | 4,01924 |
| WDR69 | WD repeat domain 69 | 5,58E-11 | 4,00103 |
| TFPI | tissue factor pathway inhibitor (lipoprotein-associated coagulation inhibitor) | 3,23E-12 | 3,89511 |
| C21orf7 | chromosome 21 open reading frame 7 | 6,41E-13 | 3,87198 |
| SLC4A4 | solute carrier family 4, sodium bicarbonate cotransporter, member 4 | 1,95E-17 | 3,80072 |
| CXADR | coxsackie virus and adenovirus receptor | 8,19E-10 | 3,75833 |
| TNFRSF11B | tumor necrosis factor receptor superfamily, member 11b | 6,38E-12 | 3,65423 |
| LOC728613 | programmed cell death 6 pseudogene | 9,82E-15 | 3,65123 |
| ADAMTSL1 | ADAMTS-like 1 | 6,84E-13 | 3,61217 |
| COL15A1 | collagen, type XV, alpha 1 | 7,31E-14 | 3,61213 |
| PLAT | plasminogen activator, tissue | 2,18E-18 | 3,59714 |
| ST7L | suppression of tumorigenicity 7 like | 1,10E-15 | 3,47925 |
| COL4A6 | collagen, type IV, alpha 6 | 3,63E-13 | 3,41602 |
| SULT1E1 | sulfotransferase family 1E, estrogen-preferring, member 1 | 2,48E-15 | 3,40958 |
| ZNF880 | zinc finger protein 880 | 7,70E-15 | 3,38601 |
| MAB21L2 | mab-21-like 2 (C. elegans) | 4,73E-16 | 3,36096 |
| SYT1 | synaptotagmin I | 6,21E-08 | 3,32606 |
| SPP1 | secreted phosphoprotein 1 | 2,68E-03 | 3,28384 |
| CLDN1 | claudin 1 | 9,25E-04 | 3,27211 |
| TM4SF1 | transmembrane 4 L six family member 1 | 1,14E-10 | 3,23899 |
| IGF2 /// INS-IGF2 | insulin-like growth factor 2 (somatomedin A) /// INS-IGF2 readthrough transcript | 3,82E-10 | 3,20307 |
| MALL | mal, T-cell differentiation protein-like | 2,99E-10 | 3,20126 |
| CFH /// CFHR1 | complement factor H /// complement factor H-related 1 | 8,96E-12 | 3,1965 |
| TOX | thymocyte selection-associated high mobility group box | 1,68E-08 | 3,16503 |
| DNAJC6 | DnaJ (Hsp40) homolog, subfamily C, member 6 | 6,67E-12 | 3,16467 |
| TSLP | thymic stromal lymphopoietin | 6,33E-06 | 3,14111 |
| A2LD1 | AIG2-like domain 1 | 7,00E-18 | 3,13836 |
| PHLDA1 | pleckstrin homology-like domain, family A, member 1 | 3,65E-12 | 3,13348 |
| COX7A1 | cytochrome c oxidase subunit VIIa polypeptide 1 (muscle) | 5,89E-18 | 3,08291 |
| HOTAIR | hox transcript antisense RNA (non-protein coding) | 1,75E-14 | 3,08021 |
| KCNQ5 | potassium voltage-gated channel, KQT-like subfamily, member 5 | 7,42E-08 | 3,06021 |
| DOCK4 | dedicator of cytokinesis 4 | 1,46E-10 | 3,01686 |
| NTM | neurotrimin | 2,17E-08 | 3,01287 |
| DCBLD2 | Discoidin, CUB and LCCL domain containing 2 | 2,61E-12 | 3,00745 |
|  | **Downregulated** |  |  |
| XIST | X (inactive)-specific transcript (non-protein coding) | 1,02E-34 | -7,04289 |
| NDN | necdin homolog (mouse) | 4,83E-28 | -6,29509 |
| BEX1 | brain expressed, X-linked 1 | 2,45E-24 | -6,10636 |
| MAB21L1 | mab-21-like 1 (C. elegans) | 4,64E-17 | -5,26257 |
| ELOVL2 | elongation of very long chain fatty acids (FEN1/Elo2, SUR4/Elo3, yeast)-like 2 | 2,55E-10 | -4,88942 |
| KYNU | kynureninase (L-kynurenine hydrolase) | 1,07E-14 | -4,88697 |
| MRVI1 | murine retrovirus integration site 1 homolog | 1,50E-14 | -4,34172 |
| ADORA2B | adenosine A2b receptor | 2,43E-24 | -4,2307 |
| RPS23 | ribosomal protein S23 | 5,52E-15 | -4,17474 |
| CCL7 | chemokine (C-C motif) ligand 7 | 1,36E-08 | -4,1251 |
| BEX4 | brain expressed, X-linked 4 | 2,58E-14 | -3,98833 |
| ACTA2 | Actin, alpha 2, smooth muscle, aorta | 3,89E-07 | -3,84535 |
| C5orf13 | chromosome 5 open reading frame 13 | 2,87E-19 | -3,78505 |
| OXTR | oxytocin receptor | 1,54E-08 | -3,71503 |
| LOC399491 | GPS, PLAT and transmembrane domain-containing protein | 2,09E-12 | -3,59603 |
| ENO1 | enolase 1, (alpha) | 2,79E-03 | -3,58285 |
| MEG3 | maternally expressed 3 (non-protein coding) | 4,26E-06 | -3,49976 |
| PPP4R4 | protein phosphatase 4, regulatory subunit 4 | 2,03E-12 | -3,4238 |
| GATA4 | GATA binding protein 4 | 2,81E-07 | -3,32781 |
| SYNPO2 | synaptopodin 2 | 4,02E-06 | -3,26304 |
| TMTC1 | transmembrane and tetratricopeptide repeat containing 1 | 2,89E-06 | -3,22966 |
| GABRB1 | gamma-aminobutyric acid (GABA) A receptor, beta 1 | 2,08E-07 | -3,2186 |
| G0S2 | G0/G1switch 2 | 2,76E-03 | -3,16497 |
| CTSK | cathepsin K | 1,03E-08 | -3,16065 |
| MORF4L2 | Mortality factor 4 like 2 | 2,08E-05 | -3,01646 |

| Supplemental File 5. Table of differentially expressed genes in senescent hMSC/inv compared to senescent hMSC/n. | **Senescent hMSC/inv vs Senescent hMSC/n** | |  |
| --- | --- | --- | --- |
| **Gene Symbol** | **Gene Title** | **p-value BF*** | **Fold change** |
|  | **Upregulated** |  |  |
| MMP1 | matrix metallopeptidase 1 (interstitial collagenase) | 2,45E-25 | 96,6301 |
| ALDH1A1 | aldehyde dehydrogenase 1 family, member A1 | 7,30E-26 | 56,2836 |
| SFRP1 | secreted frizzled-related protein 1 | 2,62E-34 | 39,0669 |
| LOC730755 | keratin associated protein 2-4-like | 1,05E-23 | 22,0128 |
| L1CAM | L1 cell adhesion molecule | 8,32E-31 | 21,3267 |
| NPPB | natriuretic peptide B | 1,37E-22 | 20,6459 |
| SCN3A | sodium channel, voltage-gated, type III, alpha subunit | 1,04E-25 | 20,1669 |
| CNTN3 | contactin 3 (plasmacytoma associated) | 3,41E-32 | 19,5117 |
| LAMC2 | laminin, gamma 2 | 1,75E-33 | 17,3405 |
| MAMDC2 | MAM domain containing 2 | 2,29E-21 | 16,9176 |
| HAPLN1 | hyaluronan and proteoglycan link protein 1 | 6,00E-16 | 16,7884 |
| C5orf46 | chromosome 5 open reading frame 46 | 9,86E-25 | 16,3464 |
| HHIP | hedgehog interacting protein | 9,92E-30 | 16,3091 |
| HOTAIR | hox transcript antisense RNA (non-protein coding) | 1,25E-33 | 16,1935 |
| HOXC10 | homeobox C10 | 1,85E-27 | 16,1698 |
| AK4 | adenylate kinase 4 | 5,66E-30 | 16,0519 |
| LOC728613 | programmed cell death 6 pseudogene | 1,79E-29 | 13,8879 |
| NEFM | neurofilament, medium polypeptide | 4,81E-25 | 13,7263 |
| ANKRD1 | ankyrin repeat domain 1 (cardiac muscle) | 1,32E-11 | 13,4826 |
| KISS1 | KiSS-1 metastasis-suppressor | 5,22E-25 | 13,2696 |
| PLN | phospholamban | 1,56E-15 | 13,0404 |
| C10orf58 | chromosome 10 open reading frame 58 | 2,02E-16 | 12,5807 |
| SLC7A2 | solute carrier family 7 (cationic amino acid transporter, y+ system), member 2 | 3,33E-19 | 12,1714 |
| DNAJC6 | DnaJ (Hsp40) homolog, subfamily C, member 6 | 3,03E-27 | 11,93 |
| CXADR | coxsackie virus and adenovirus receptor | 1,68E-21 | 11,8676 |
| NRK | Nik related kinase | 4,21E-32 | 11,6697 |
| LOC400043 | hypothetical LOC400043 | 2,92E-16 | 11,5853 |
| NTN4 | netrin 4 | 4,41E-17 | 11,4886 |
| CADM1 | cell adhesion molecule 1 | 5,44E-24 | 10,9342 |
| CD274 | CD274 molecule | 1,17E-16 | 10,4025 |
| KCNT2 | potassium channel, subfamily T, member 2 | 6,05E-21 | 9,65584 |
| EGF | epidermal growth factor | 6,99E-22 | 9,31693 |
| RGMB | RGM domain family, member B | 1,39E-17 | 9,01317 |
| FAR2 | Fatty acyl CoA reductase 2 | 4,89E-27 | 8,60828 |
| TPD52L1 | tumor protein D52-like 1 | 1,11E-20 | 8,45075 |
| COL4A2 | collagen, type IV, alpha 2 | 5,28E-14 | 8,33256 |
| OLR1 | oxidized low density lipoprotein (lectin-like) receptor 1 | 1,51E-20 | 7,72385 |
| GALNT3 | UDP-N-acetyl-alpha-D-galactosamine:polypeptide N-acetylgalactosaminyltransferase | 2,83E-15 | 7,7065 |
| TNFRSF21 | tumor necrosis factor receptor superfamily, member 21 | 6,49E-23 | 7,57155 |
| GRP | gastrin-releasing peptide | 4,93E-14 | 7,50514 |
| ABLIM1 | actin binding LIM protein 1 | 5,80E-24 | 7,33942 |
| CRYAB | crystallin, alpha B | 4,61E-14 | 6,88616 |
| RGS4 | regulator of G-protein signaling 4 | 2,50E-14 | 6,68146 |
| RGS5 | regulator of G-protein signaling 5 | 6,41E-22 | 6,66255 |
| SLC24A3 | solute carrier family 24 (sodium/potassium/calcium exchanger), member 3 | 1,76E-23 | 6,57076 |
| BCHE | butyrylcholinesterase | 3,28E-19 | 6,51377 |
| LOC654433 | hypothetical LOC654433 | 5,01E-25 | 6,42081 |
| HBEGF | heparin-binding EGF-like growth factor | 1,14E-22 | 6,39469 |
| SUSD2 | sushi domain containing 2 | 7,69E-21 | 6,33222 |
| CDH8 | cadherin 8, type 2 | 1,09E-25 | 6,32761 |
| TPD52 | tumor protein D52 | 4,19E-18 | 6,29187 |
| EDN1 | endothelin 1 | 5,62E-22 | 6,19263 |
| ARID5B | AT rich interactive domain 5B (MRF1-like) | 7,85E-18 | 6,02746 |
| PRICKLE1 | prickle homolog 1 (Drosophila) | 4,83E-25 | 5,97265 |
| NOG | noggin | 8,91E-10 | 5,69388 |
| TM4SF1 | transmembrane 4 L six family member 1 | 2,46E-14 | 5,63546 |
| ARHGAP29 | Rho GTPase activating protein 29 | 2,81E-19 | 5,57186 |
| PHLDB2 | pleckstrin homology-like domain, family B, member 2 | 1,22E-08 | 5,46833 |
| CHRM3 | cholinergic receptor, muscarinic 3 | 2,18E-16 | 5,45051 |
| COL25A1 | collagen, type XXV, alpha 1 | 3,84E-13 | 5,44825 |
| IGFBP5 | insulin-like growth factor binding protein 5 | 9,05E-13 | 5,3603 |
| FOXE1 | forkhead box E1 (thyroid transcription factor 2) | 3,92E-15 | 5,27988 |
| GSTT2 | glutathione S-transferase theta 2 | 1,13E-20 | 5,25695 |
| PTGFRN | prostaglandin F2 receptor negative regulator | 1,37E-13 | 5,18247 |
| TNFRSF11B | tumor necrosis factor receptor superfamily, member 11b | 3,43E-16 | 5,14099 |
| MFAP5 | microfibrillar associated protein 5 | 2,90E-13 | 5,10198 |
| TRBC1 /// TRBC2 | T cell receptor beta constant 1 /// T cell receptor beta constant 2 | 4,47E-14 | 5,08197 |
| DSC2 | desmocollin 2 | 2,40E-18 | 5,0543 |
| ADAM23 | ADAM metallopeptidase domain 23 | 3,08E-21 | 5,05061 |
| IGF2 /// INS-IGF2 | insulin-like growth factor 2 (somatomedin A) /// INS-IGF2 readthrough transcript | 1,05E-15 | 4,92893 |
| AGTR1 | angiotensin II receptor, type 1 | 6,94E-15 | 4,90909 |
| ATP8A1 | ATPase, aminophospholipid transporter (APLT), class I, type 8A, member 1 | 3,62E-21 | 4,85526 |
| GPRC5A | G protein-coupled receptor, family C, group 5, member A | 1,71E-20 | 4,78254 |
| DLG1 | discs, large homolog 1 (Drosophila) | 1,48E-10 | 4,75775 |
| PDCD1LG2 | programmed cell death 1 ligand 2 | 2,99E-13 | 4,74131 |
| TGFB2 | transforming growth factor, beta 2 | 1,60E-12 | 4,65836 |
| SH3RF2 | SH3 domain containing ring finger 2 | 4,00E-17 | 4,64978 |
| SERTAD4 | SERTA domain containing 4 | 2,38E-16 | 4,6266 |
| JAM2 | junctional adhesion molecule 2 | 1,79E-12 | 4,58682 |
| EPPK1 | epiplakin 1 | 5,20E-17 | 4,57703 |
| LOC100505470 /// LOC646576 | hypothetical LOC100505470 /// hypothetical LOC646576 | 2,92E-14 | 4,53172 |
| ZPLD1 | zona pellucida-like domain containing 1 | 1,67E-12 | 4,52553 |
| ARAP2 | ArfGAP with RhoGAP domain, ankyrin repeat and PH domain 2 | 2,47E-14 | 4,49183 |
| PLAT | plasminogen activator, tissue | 1,41E-21 | 4,46367 |
| SCUBE3 | signal peptide, CUB domain, EGF-like 3 | 2,47E-07 | 4,46227 |
| FLT1 | fms-related tyrosine kinase 1 (vascular endothelial growth factor/vascular permea | 5,52E-10 | 4,46141 |
| RPL31 | ribosomal protein L31 | 2,09E-18 | 4,44763 |
| PCOLCE2 | procollagen C-endopeptidase enhancer 2 | 1,15E-17 | 4,42416 |
| C15orf54 | chromosome 15 open reading frame 54 | 5,76E-17 | 4,41239 |
| SCD5 | stearoyl-CoA desaturase 5 | 1,20E-18 | 4,3752 |
| CNTN1 | Contactin 1 | 1,98E-13 | 4,35623 |
| HLA-DPA1 | major histocompatibility complex, class II, DP alpha 1 | 3,94E-17 | 4,33503 |
| CNTNAP3 | contactin associated protein-like 3 | 9,55E-15 | 4,31896 |
| PHKA1 | phosphorylase kinase, alpha 1 (muscle) | 3,20E-18 | 4,2578 |
| KCTD20 | potassium channel tetramerisation domain containing 20 | 1,21E-22 | 4,24464 |
| LOC285628 | hypothetical protein LOC285628 | 1,69E-06 | 4,19179 |
| SRGN | serglycin | 5,18E-17 | 4,16015 |
| EDIL3 | EGF-like repeats and discoidin I-like domains 3 | 7,94E-08 | 4,14482 |
| NEDD4L | neural precursor cell expressed, developmentally down-regulated 4-like | 4,91E-15 | 4,13967 |
| CCDC81 | coiled-coil domain containing 81 | 3,30E-11 | 4,04453 |
| SYNPO2 | synaptopodin 2 | 3,08E-13 | 3,99263 |
| FHL1 | four and a half LIM domains 1 | 5,78E-10 | 3,93926 |
| TNFRSF10D | tumor necrosis factor receptor superfamily, member 10d, decoy with truncated deat | 4,74E-17 | 3,87082 |
| ANKRD37 | ankyrin repeat domain 37 | 6,64E-15 | 3,83403 |
| IL11 | interleukin 11 | 3,35E-12 | 3,81313 |
| TSPAN2 | tetraspanin 2 | 1,74E-12 | 3,79212 |
| CCND2 | cyclin D2 | 3,60E-14 | 3,79202 |
| LYPD6B | LY6/PLAUR domain containing 6B | 2,28E-16 | 3,78424 |
| AQP1 | aquaporin 1 (Colton blood group) | 1,35E-13 | 3,77163 |
| MCAM | melanoma cell adhesion molecule | 3,40E-20 | 3,76729 |
| FAM84B | family with sequence similarity 84, member B | 9,44E-07 | 3,75514 |
| MAB21L2 | mab-21-like 2 (C. elegans) | 1,06E-17 | 3,74137 |
| RCAN1 | regulator of calcineurin 1 | 1,79E-08 | 3,73659 |
| ATP7B | ATPase, Cu++ transporting, beta polypeptide | 6,84E-19 | 3,73335 |
| JAG1 | jagged 1 | 1,10E-13 | 3,70545 |
| DBNDD2 /// SYS1 /// SYS1-DBNDD2 | dysbindin (dystrobrevin binding protein 1) domain containing 2 /// SYS1 Golgi-loc | 2,98E-20 | 3,6737 |
| RUNX1 | runt-related transcription factor 1 | 1,47E-18 | 3,65248 |
| HCLS1 | hematopoietic cell-specific Lyn substrate 1 | 1,07E-16 | 3,65028 |
| KCTD4 | potassium channel tetramerisation domain containing 4 | 6,75E-12 | 3,61333 |
| CALD1 | caldesmon 1 | 3,80E-05 | 3,59851 |
| PLS1 | plastin 1 | 1,63E-15 | 3,59025 |
| GRIA3 | glutamate receptor, ionotrophic, AMPA 3 | 8,95E-15 | 3,58865 |
| ARSJ | arylsulfatase family, member J | 4,56E-22 | 3,58483 |
| MPP7 | membrane protein, palmitoylated 7 (MAGUK p55 subfamily member 7) | 9,50E-16 | 3,55506 |
| HEPH | hephaestin | 1,67E-16 | 3,5293 |
| NFIB | nuclear factor I/B | 9,50E-12 | 3,5117 |
| TNC | tenascin C | 6,79E-17 | 3,50049 |
| ADAMTS3 | ADAM metallopeptidase with thrombospondin type 1 motif, 3 | 5,58E-10 | 3,45227 |
| CDH10 | cadherin 10, type 2 (T2-cadherin) | 2,65E-15 | 3,44412 |
| RGNEF | 190 kDa guanine nucleotide exchange factor | 1,60E-08 | 3,40782 |
| CACNA1A | calcium channel, voltage-dependent, P/Q type, alpha 1A subunit | 8,17E-15 | 3,39945 |
| DSC3 | desmocollin 3 | 3,55E-18 | 3,32568 |
| EFNB2 | ephrin-B2 | 8,30E-13 | 3,30776 |
| QPCT | glutaminyl-peptide cyclotransferase | 1,43E-11 | 3,29213 |
| LINGO2 | leucine rich repeat and Ig domain containing 2 | 4,99E-11 | 3,27479 |
| LAMA5 | laminin, alpha 5 | 1,23E-16 | 3,2475 |
| COL8A1 | collagen, type VIII, alpha 1 | 9,83E-05 | 3,24208 |
| GAS6 | growth arrest-specific 6 | 2,71E-17 | 3,23332 |
| SGCD | sarcoglycan, delta (35kDa dystrophin-associated glycoprotein) | 2,82E-08 | 3,22708 |
| SLC16A3 | solute carrier family 16, member 3 (monocarboxylic acid transporter 4) | 3,69E-05 | 3,22673 |
| SHISA3 | shisa homolog 3 (Xenopus laevis) | 1,72E-18 | 3,20652 |
| GLIPR1 | GLI pathogenesis-related 1 | 2,64E-22 | 3,20296 |
| DAB2 | disabled homolog 2, mitogen-responsive phosphoprotein (Drosophila) | 0,001301 | 3,20156 |
| MYCT1 | myc target 1 | 1,83E-08 | 3,19443 |
| EPHA3 | EPH receptor A3 | 2,86E-11 | 3,1912 |
| MYEF2 | myelin expression factor 2 | 4,30E-14 | 3,18606 |
| FGF1 | fibroblast growth factor 1 (acidic) | 2,47E-12 | 3,18361 |
| SORBS1 | sorbin and SH3 domain containing 1 | 1,08E-14 | 3,15278 |
| CDCP1 | CUB domain containing protein 1 | 1,50E-08 | 3,14719 |
| PLCB4 | phospholipase C, beta 4 | 3,12E-06 | 3,14375 |
| SMURF2 | SMAD specific E3 ubiquitin protein ligase 2 | 9,86E-09 | 3,10896 |
| WNT16 | wingless-type MMTV integration site family, member 16 | 1,73E-09 | 3,09481 |
| C6orf99 | chromosome 6 open reading frame 99 | 1,68E-11 | 3,08793 |
| CYB5R2 | cytochrome b5 reductase 2 | 6,32E-18 | 3,07171 |
| PALMD | palmdelphin | 7,69E-18 | 3,04127 |
| CD200 | CD200 molecule | 1,99E-08 | 3,02666 |
| FPR1 | formyl peptide receptor 1 | 2,96E-11 | 3,01313 |
| DSP | desmoplakin | 2,47E-06 | 3,00967 |
|  | **Downregulated** |  |  |
| HGF | hepatocyte growth factor (hepapoietin A; scatter factor) | 2,37E-31 | -37,7134 |
| IL33 | interleukin 33 | 8,66E-20 | -36,865 |
| G0S2 | G0/G1switch 2 | 2,01E-27 | -28,6178 |
| DOK5 | docking protein 5 | 2,00E-26 | -27,8488 |
| KYNU | kynureninase (L-kynurenine hydrolase) | 2,15E-28 | -21,6892 |
| TNFAIP6 | tumor necrosis factor, alpha-induced protein 6 | 4,26E-23 | -18,5556 |
| NDN | necdin homolog (mouse) | 5,67E-38 | -17,2732 |
| ITGBL1 | integrin, beta-like 1 (with EGF-like repeat domains) | 3,93E-22 | -15,9321 |
| LRRC17 | leucine rich repeat containing 17 | 7,51E-26 | -15,1663 |
| CHI3L1 | chitinase 3-like 1 (cartilage glycoprotein-39) | 1,86E-09 | -14,5364 |
| F2RL1 | coagulation factor II (thrombin) receptor-like 1 | 7,91E-19 | -14,3471 |
| GATA3 | GATA binding protein 3 | 6,13E-28 | -13,6379 |
| CARD16 | caspase recruitment domain family, member 16 | 4,98E-26 | -13,4806 |
| LOC100132891 | hypothetical LOC100132891 | 5,67E-23 | -12,0172 |
| TMTC1 | transmembrane and tetratricopeptide repeat containing 1 | 1,08E-17 | -11,8544 |
| MASP1 | mannan-binding lectin serine peptidase 1 (C4/C2 activating component of Ra-reacti | 6,81E-28 | -11,6694 |
| VAMP8 | vesicle-associated membrane protein 8 (endobrevin) | 8,49E-27 | -11,6281 |
| IQGAP2 | IQ motif containing GTPase activating protein 2 | 1,17E-15 | -10,6731 |
| STMN2 | stathmin-like 2 | 5,21E-12 | -10,6187 |
| GPNMB | glycoprotein (transmembrane) nmb | 1,13E-08 | -10,5814 |
| C1R | complement component 1, r subcomponent | 7,07E-21 | -10,4225 |
| NAP1L3 | nucleosome assembly protein 1-like 3 | 1,70E-23 | -10,1453 |
| IFIT3 | interferon-induced protein with tetratricopeptide repeats 3 | 9,78E-12 | -9,91912 |
| CYP1B1 | cytochrome P450, family 1, subfamily B, polypeptide 1 | 2,33E-09 | -9,05579 |
| NUPR1 | nuclear protein, transcriptional regulator, 1 | 2,84E-13 | -8,98737 |
| E2F7 | E2F transcription factor 7 | 8,10E-20 | -8,9307 |
| CASP1 | caspase 1, apoptosis-related cysteine peptidase (interleukin 1, beta, convertase) | 2,74E-22 | -8,73738 |
| CCL7 | chemokine (C-C motif) ligand 7 | 1,04E-15 | -8,538 |
| LMCD1 | LIM and cysteine-rich domains 1 | 2,12E-23 | -8,22886 |
| APOBEC3B | apolipoprotein B mRNA editing enzyme, catalytic polypeptide-like 3B | 1,88E-12 | -7,87537 |
| TSHZ1 | teashirt zinc finger homeobox 1 | 5,25E-19 | -7,66108 |
| FST | follistatin | 1,06E-26 | -7,51366 |
| GXYLT2 | glucoside xylosyltransferase 2 | 4,67E-25 | -7,46392 |
| IFIT1 | interferon-induced protein with tetratricopeptide repeats 1 | 4,50E-05 | -7,40654 |
| KCTD12 | potassium channel tetramerisation domain containing 12 | 2,78E-21 | -7,33004 |
| ADORA2B | adenosine A2b receptor | 3,12E-31 | -7,17501 |
| UACA | uveal autoantigen with coiled-coil domains and ankyrin repeats | 5,64E-33 | -7,11174 |
| LAMA1 | laminin, alpha 1 | 6,52E-12 | -6,7933 |
| PTGIS | prostaglandin I2 (prostacyclin) synthase | 1,48E-16 | -6,52039 |
| HTR2B | 5-hydroxytryptamine (serotonin) receptor 2B | 2,18E-08 | -6,49987 |
| GPC6 | glypican 6 | 8,42E-17 | -6,42502 |
| SCIN | scinderin | 4,71E-06 | -6,35289 |
| MME | membrane metallo-endopeptidase | 3,54E-20 | -6,35099 |
| RUNX2 | runt-related transcription factor 2 | 1,05E-17 | -6,24807 |
| GALNT12 | UDP-N-acetyl-alpha-D-galactosamine:polypeptide N-acetylgalactosaminyltransferase | 6,34E-20 | -6,22537 |
| SLC38A4 | solute carrier family 38, member 4 | 1,09E-14 | -6,14599 |
| EPSTI1 | epithelial stromal interaction 1 (breast) | 1,39E-11 | -6,10991 |
| TRIM58 | tripartite motif-containing 58 | 1,24E-14 | -6,08121 |
| ELOVL2 | elongation of very long chain fatty acids (FEN1/Elo2, SUR4/Elo3, yeast)-like 2 | 1,70E-12 | -6,08066 |
| EGR2 | early growth response 2 | 1,15E-05 | -6,03208 |
| CHN1 | chimerin (chimaerin) 1 | 2,53E-28 | -6,02721 |
| ATP10A | ATPase, class V, type 10A | 4,23E-18 | -5,93013 |
| FOXF1 | forkhead box F1 | 1,37E-27 | -5,91731 |
| HMMR | hyaluronan-mediated motility receptor (RHAMM) | 1,74E-10 | -5,91415 |
| PDZRN3 | PDZ domain containing ring finger 3 | 1,98E-12 | -5,90235 |
| LOC389834 | ankyrin repeat domain 57 pseudogene | 2,90E-21 | -5,85378 |
| BEX4 | brain expressed, X-linked 4 | 4,90E-19 | -5,845 |
| FBLN5 | fibulin 5 | 4,89E-14 | -5,80767 |
| ITGB3BP | integrin beta 3 binding protein (beta3-endonexin) | 8,24E-17 | -5,80648 |
| TMEM97 | transmembrane protein 97 | 9,68E-21 | -5,71228 |
| PAG1 | phosphoprotein associated with glycosphingolipid microdomains 1 | 1,21E-13 | -5,6985 |
| DIRAS3 | DIRAS family, GTP-binding RAS-like 3 | 2,27E-09 | -5,66888 |
| CDKN3 | cyclin-dependent kinase inhibitor 3 | 1,14E-05 | -5,62609 |
| ST6GAL2 | ST6 beta-galactosamide alpha-2,6-sialyltranferase 2 | 9,79E-17 | -5,59987 |
| P4HA3 | prolyl 4-hydroxylase, alpha polypeptide III | 1,29E-17 | -5,57949 |
| PTPRG | protein tyrosine phosphatase, receptor type, G | 1,76E-18 | -5,54889 |
| GATA4 | GATA binding protein 4 | 7,02E-13 | -5,51422 |
| SLC12A8 | solute carrier family 12 (potassium/chloride transporters), member 8 | 4,38E-15 | -5,51033 |
| METTL7A | methyltransferase like 7A | 5,19E-14 | -5,4539 |
| CRISPLD2 | cysteine-rich secretory protein LCCL domain containing 2 | 4,82E-17 | -5,43596 |
| WFDC1 | WAP four-disulfide core domain 1 | 1,43E-06 | -5,42781 |
| PSG5 | pregnancy specific beta-1-glycoprotein 5 | 1,07E-05 | -5,39071 |
| KLF4 | Kruppel-like factor 4 (gut) | 1,57E-07 | -5,36634 |
| SLC14A1 | solute carrier family 14 (urea transporter), member 1 (Kidd blood group) | 8,05E-12 | -5,34307 |
| GEM | GTP binding protein overexpressed in skeletal muscle | 9,55E-17 | -5,27515 |
| SEMA6D | sema domain, transmembrane domain (TM), and cytoplasmic domain, (semaphorin) 6D | 4,40E-17 | -5,2369 |
| HSD17B2 | hydroxysteroid (17-beta) dehydrogenase 2 | 4,12E-12 | -5,2137 |
| KIF20A | kinesin family member 20A | 4,18E-07 | -5,03822 |
| PSMB9 | proteasome (prosome, macropain) subunit, beta type, 9 (large multifunctional pept | 5,39E-15 | -5,03317 |
| PTPLAD2 | protein tyrosine phosphatase-like A domain containing 2 | 8,46E-22 | -4,93447 |
| MXRA5 | matrix-remodelling associated 5 | 3,11E-06 | -4,91685 |
| C1S | complement component 1, s subcomponent | 5,32E-12 | -4,91257 |
| INSIG1 | insulin induced gene 1 | 2,33E-10 | -4,89585 |
| TCF21 | transcription factor 21 | 6,56E-14 | -4,8901 |
| GNG2 | guanine nucleotide binding protein (G protein), gamma 2 | 2,64E-20 | -4,89005 |
| NCAM2 | neural cell adhesion molecule 2 | 7,05E-15 | -4,87759 |
| TGFBI | transforming growth factor, beta-induced, 68kDa | 2,23E-25 | -4,78415 |
| PARP9 | poly (ADP-ribose) polymerase family, member 9 | 1,57E-10 | -4,73708 |
| CDC20 | cell division cycle 20 homolog (S. cerevisiae) | 1,13E-05 | -4,73213 |
| SLFN11 | schlafen family member 11 | 8,15E-18 | -4,70239 |
| EGR1 | Early growth response 1 | 8,55E-13 | -4,70173 |
| CCNA2 | cyclin A2 | 8,29E-06 | -4,61152 |
| LOC339803 | hypothetical LOC339803 | 3,73E-16 | -4,60033 |
| MYO10 | myosin X | 2,70E-17 | -4,595 |
| DLGAP5 | discs, large (Drosophila) homolog-associated protein 5 | 3,02E-06 | -4,59105 |
| PTGER2 | prostaglandin E receptor 2 (subtype EP2), 53kDa | 4,13E-13 | -4,57138 |
| DEPDC1 | DEP domain containing 1 | 1,52E-07 | -4,50164 |
| ENPP1 | ectonucleotide pyrophosphatase/phosphodiesterase 1 | 1,26E-18 | -4,49543 |
| C15orf48 | chromosome 15 open reading frame 48 | 1,83E-08 | -4,45311 |
| C14orf159 | chromosome 14 open reading frame 159 | 4,28E-18 | -4,44824 |
| FIBIN | fin bud initiation factor homolog (zebrafish) | 5,66E-08 | -4,43361 |
| IFIH1 | interferon induced with helicase C domain 1 | 6,82E-05 | -4,41344 |
| PMP22 | peripheral myelin protein 22 | 3,69E-22 | -4,32681 |
| FAM129A | family with sequence similarity 129, member A | 5,91E-07 | -4,32492 |
| IFITM1 | interferon induced transmembrane protein 1 (9-27) | 0,000102 | -4,31422 |
| SOBP | sine oculis binding protein homolog (Drosophila) | 3,14E-17 | -4,30636 |
| PABPC4L | poly(A) binding protein, cytoplasmic 4-like | 4,75E-11 | -4,24978 |
| FGF7 /// KGFLP1 /// KGFLP2 | fibroblast growth factor 7 /// keratinocyte growth factor-like protein 1 /// kera | 1,59E-13 | -4,22881 |
| UNC5B | unc-5 homolog B (C. elegans) | 0,000674 | -4,22445 |
| FBXL13 | F-box and leucine-rich repeat protein 13 | 1,45E-11 | -4,21712 |
| IFI44L | interferon-induced protein 44-like | 0,007287 | -4,19432 |
| USP18 | ubiquitin specific peptidase 18 | 3,11E-09 | -4,17771 |
| LOC100507248 | hypothetical LOC100507248 | 3,99E-16 | -4,17489 |
| CTSK | cathepsin K | 3,36E-12 | -4,15889 |
| MLF1IP | MLF1 interacting protein | 1,34E-05 | -4,15837 |
| CREG1 | cellular repressor of E1A-stimulated genes 1 | 6,43E-13 | -4,1447 |
| MOCOS | molybdenum cofactor sulfurase | 3,60E-10 | -4,13521 |
| UCP2 | uncoupling protein 2 (mitochondrial, proton carrier) | 9,05E-11 | -4,13469 |
| LAMA2 | laminin, alpha 2 | 4,35E-16 | -4,12406 |
| CDK1 | cyclin-dependent kinase 1 | 7,54E-06 | -4,11829 |
| BUB1 | budding uninhibited by benzimidazoles 1 homolog (yeast) | 3,47E-08 | -4,10745 |
| ETV1 | ets variant 1 | 2,49E-15 | -4,10111 |
| ANGPTL1 | angiopoietin-like 1 | 3,36E-05 | -4,09003 |
| CTHRC1 | collagen triple helix repeat containing 1 | 6,40E-19 | -4,07901 |
| NEK2 | NIMA (never in mitosis gene a)-related kinase 2 | 4,09E-07 | -4,06013 |
| LMNB1 | lamin B1 | 5,13E-06 | -4,05173 |
| VDR | vitamin D (1,25- dihydroxyvitamin D3) receptor | 7,09E-10 | -4,04766 |
| PBK | PDZ binding kinase | 0,000369 | -4,04753 |
| TRPA1 | transient receptor potential cation channel, subfamily A, member 1 | 1,32E-07 | -4,04513 |
| FLRT3 | fibronectin leucine rich transmembrane protein 3 | 5,74E-09 | -4,03218 |
| MMP2 | matrix metallopeptidase 2 (gelatinase A, 72kDa gelatinase, 72kDa type IV collagen | 1,70E-13 | -4,02033 |
| XAF1 | XIAP associated factor 1 | 8,76E-07 | -4,01909 |
| ARMC9 | armadillo repeat containing 9 | 8,25E-13 | -4,01562 |
| CCNB2 | cyclin B2 | 3,27E-06 | -4,01482 |
| TYRP1 | tyrosinase-related protein 1 | 1,40E-10 | -4,01 |
| DEPDC6 | DEP domain containing 6 | 3,04E-09 | -4,00584 |
| UBE2L6 | ubiquitin-conjugating enzyme E2L 6 | 1,13E-12 | -4,00047 |
| PDE4DIP | phosphodiesterase 4D interacting protein | 3,23E-18 | -3,99946 |
| C5orf13 | chromosome 5 open reading frame 13 | 4,57E-22 | -3,95788 |
| DOCK11 | dedicator of cytokinesis 11 | 1,95E-22 | -3,93755 |
| C3orf55 | chromosome 3 open reading frame 55 | 2,99E-15 | -3,93043 |
| LOC84856 | hypothetical LOC84856 | 1,58E-17 | -3,92014 |
| CDKN2C | cyclin-dependent kinase inhibitor 2C (p18, inhibits CDK4) | 9,12E-06 | -3,90454 |
| SLC2A5 | solute carrier family 2 (facilitated glucose/fructose transporter), member 5 | 1,69E-05 | -3,89166 |
| TTK | TTK protein kinase | 1,21E-05 | -3,87651 |
| PBX1 | pre-B-cell leukemia homeobox 1 | 9,56E-08 | -3,8454 |
| AQP3 | aquaporin 3 (Gill blood group) | 7,29E-10 | -3,82385 |
| COL6A3 | collagen, type VI, alpha 3 | 9,86E-29 | -3,75557 |
| RNASEL | ribonuclease L (2',5'-oligoisoadenylate synthetase-dependent) | 1,79E-13 | -3,75461 |
| DTL | denticleless homolog (Drosophila) | 7,43E-05 | -3,75155 |
| MAB21L1 | mab-21-like 1 (C. elegans) | 8,58E-13 | -3,7473 |
| ANXA10 | annexin A10 | 6,34E-11 | -3,73476 |
| BIRC5 | baculoviral IAP repeat-containing 5 | 0,000427 | -3,72975 |
| NDC80 | NDC80 homolog, kinetochore complex component (S. cerevisiae) | 8,73E-07 | -3,71959 |
| PYCARD | PYD and CARD domain containing | 1,50E-11 | -3,71463 |
| PLSCR1 | phospholipid scramblase 1 | 5,74E-08 | -3,71378 |
| HERC6 | hect domain and RLD 6 | 0,002331 | -3,68698 |
| LOC100288525 | hypothetical protein LOC100288525 | 4,36E-14 | -3,68092 |
| PLA2G4C | phospholipase A2, group IVC (cytosolic, calcium-independent) | 0,000226 | -3,68043 |
| LOC100505806 /// SNORD123 | hypothetical LOC100505806 /// small nucleolar RNA, C/D box 123 | 1,83E-22 | -3,67826 |
| CCDC99 | coiled-coil domain containing 99 | 2,24E-15 | -3,67021 |
| OIP5 | Opa interacting protein 5 | 2,59E-05 | -3,66804 |
| TDO2 | tryptophan 2,3-dioxygenase | 2,49E-09 | -3,66345 |
| CCDC85A | coiled-coil domain containing 85A | 2,76E-11 | -3,65155 |
| AIM1 | absent in melanoma 1 | 3,69E-09 | -3,64767 |
| TOP2A | topoisomerase (DNA) II alpha 170kDa | 2,04E-05 | -3,64281 |
| PDGFRA | platelet-derived growth factor receptor, alpha polypeptide | 4,60E-22 | -3,64262 |
| SPON2 | spondin 2, extracellular matrix protein | 4,45E-15 | -3,57526 |
| QPRT | quinolinate phosphoribosyltransferase | 4,77E-13 | -3,56413 |
| PRIM1 | primase, DNA, polypeptide 1 (49kDa) | 2,61E-06 | -3,56049 |
| GLB1L | galactosidase, beta 1-like | 7,04E-14 | -3,56044 |
| CCDC34 | coiled-coil domain containing 34 | 6,42E-07 | -3,55579 |
| GOLM1 | golgi membrane protein 1 | 3,62E-25 | -3,55237 |
| DDX60 | DEAD (Asp-Glu-Ala-Asp) box polypeptide 60 | 7,95E-05 | -3,5506 |
| XYLT1 | xylosyltransferase I | 4,07E-09 | -3,54685 |
| SAMD9L | sterile alpha motif domain containing 9-like | 1,39E-08 | -3,54388 |
| PPP4R4 | protein phosphatase 4, regulatory subunit 4 | 6,95E-13 | -3,53832 |
| GTSE1 | G-2 and S-phase expressed 1 | 0,000109 | -3,52728 |
| B4GALT1 | UDP-Gal:betaGlcNAc beta 1,4- galactosyltransferase, polypeptide 1 | 1,06E-18 | -3,52293 |
| PKDCC | protein kinase domain containing, cytoplasmic homolog (mouse) | 3,45E-18 | -3,51663 |
| GAS2L3 | Growth arrest-specific 2 like 3 | 1,24E-10 | -3,50095 |
| HOXA13 | homeobox A13 | 2,54E-07 | -3,49793 |
| CENPW | centromere protein W | 6,44E-06 | -3,4832 |
| BTN3A2 | butyrophilin, subfamily 3, member A2 | 3,08E-15 | -3,4621 |
| NUSAP1 | nucleolar and spindle associated protein 1 | 0,000269 | -3,43256 |
| CYBASC3 | cytochrome b, ascorbate dependent 3 | 3,16E-17 | -3,40248 |
| ST3GAL6 | ST3 beta-galactoside alpha-2,3-sialyltransferase 6 | 3,52E-07 | -3,3875 |
| TTC32 | tetratricopeptide repeat domain 32 | 2,75E-12 | -3,38312 |
| PCDH18 | protocadherin 18 | 2,98E-11 | -3,38303 |
| IFIT2 | interferon-induced protein with tetratricopeptide repeats 2 | 0,000177 | -3,38292 |
| GINS2 | GINS complex subunit 2 (Psf2 homolog) | 0,000359 | -3,37794 |
| CASC5 | cancer susceptibility candidate 5 | 1,65E-05 | -3,37287 |
| FAM46A | family with sequence similarity 46, member A | 1,17E-22 | -3,36927 |
| CENPA | centromere protein A | 1,67E-05 | -3,36698 |
| RRM2 | ribonucleotide reductase M2 | 0,006179 | -3,33791 |
| GPR68 | G protein-coupled receptor 68 | 3,40E-05 | -3,33688 |
| PTPRE | protein tyrosine phosphatase, receptor type, E | 4,66E-08 | -3,33306 |
| C11orf75 | chromosome 11 open reading frame 75 | 1,28E-15 | -3,32169 |
| AKR1C3 | aldo-keto reductase family 1, member C3 (3-alpha hydroxysteroid dehydrogenase, ty | 2,62E-10 | -3,31104 |
| BHLHE40 | basic helix-loop-helix family, member e40 | 1,88E-09 | -3,29618 |
| KIF2C | kinesin family member 2C | 4,79E-05 | -3,29249 |
| CENPF | centromere protein F, 350/400kDa (mitosin) | 3,14E-07 | -3,28957 |
| SPATA13 | spermatogenesis associated 13 | 3,83E-11 | -3,28658 |
| ZNF395 | zinc finger protein 395 | 9,95E-13 | -3,27395 |
| DDX60L | DEAD (Asp-Glu-Ala-Asp) box polypeptide 60-like | 5,76E-07 | -3,27196 |
| LOXL4 | lysyl oxidase-like 4 | 2,90E-08 | -3,26588 |
| KSR1 | kinase suppressor of ras 1 | 1,26E-16 | -3,25837 |
| RHOU | ras homolog gene family, member U | 1,92E-11 | -3,2481 |
| SP110 | SP110 nuclear body protein | 1,07E-05 | -3,24077 |
| FAM118A | family with sequence similarity 118, member A | 3,62E-12 | -3,23806 |
| XG | Xg blood group | 2,00E-14 | -3,23796 |
| CHURC1 | churchill domain containing 1 | 9,95E-27 | -3,23249 |
| CEP55 | centrosomal protein 55kDa | 0,001759 | -3,23153 |
| WDR66 | WD repeat domain 66 | 1,79E-15 | -3,22967 |
| FAM72A /// FAM72B /// FAM72C /// FAM72D | family with sequence similarity 72, member A /// family with sequence similarity | 1,55E-06 | -3,22815 |
| NMI | N-myc (and STAT) interactor | 3,45E-16 | -3,22392 |
| KIAA0101 | KIAA0101 | 0,000972 | -3,2216 |
| NRP1 | neuropilin 1 | 1,59E-16 | -3,20814 |
| RGS17 | regulator of G-protein signaling 17 | 1,49E-09 | -3,20135 |
| GPRC5B | G protein-coupled receptor, family C, group 5, member B | 1,41E-14 | -3,20129 |
| KIF4A | kinesin family member 4A | 4,79E-05 | -3,19168 |
| PCDH9 | protocadherin 9 | 2,88E-10 | -3,18255 |
| RUNX1T1 | runt-related transcription factor 1; translocated to, 1 (cyclin D-related) | 5,64E-05 | -3,17382 |
| SPC25 | SPC25, NDC80 kinetochore complex component, homolog (S. cerevisiae) | 0,003241 | -3,16986 |
| FAM117B | family with sequence similarity 117, member B | 1,90E-07 | -3,15937 |
| CKAP2L | cytoskeleton associated protein 2-like | 1,67E-06 | -3,15845 |
| CTSH | cathepsin H | 3,34E-08 | -3,15245 |
| LIMCH1 | LIM and calponin homology domains 1 | 3,33E-11 | -3,14955 |
| CCNB1 | cyclin B1 | 0,000147 | -3,14949 |
| SGOL2 | shugoshin-like 2 (S. pombe) | 2,42E-06 | -3,14736 |
| NCRNA00292 | non-protein coding RNA 292 | 6,40E-13 | -3,14712 |
| CHCHD5 | coiled-coil-helix-coiled-coil-helix domain containing 5 | 6,54E-07 | -3,1365 |
| FOXM1 | forkhead box M1 | 0,000275 | -3,12747 |
| BCAT1 | branched chain amino-acid transaminase 1, cytosolic | 2,65E-14 | -3,12529 |
| RBM47 | RNA binding motif protein 47 | 2,55E-16 | -3,12484 |
| GK | glycerol kinase | 3,72E-10 | -3,11966 |
| MT1E | metallothionein 1E | 7,75E-20 | -3,11191 |
| BDKRB2 | bradykinin receptor B2 | 6,90E-11 | -3,11006 |
| PTGS1 | prostaglandin-endoperoxide synthase 1 (prostaglandin G/H synthase and cyclooxygen | 3,96E-09 | -3,10799 |
| RAB27B | RAB27B, member RAS oncogene family | 1,39E-07 | -3,10551 |
| GLT8D2 | glycosyltransferase 8 domain containing 2 | 6,34E-16 | -3,10384 |
| PRC1 | protein regulator of cytokinesis 1 | 0,003304 | -3,10338 |
| AUTS2 | autism susceptibility candidate 2 | 9,81E-15 | -3,10127 |
| TK1 | thymidine kinase 1, soluble | 2,85E-06 | -3,10117 |
| TMCC3 | transmembrane and coiled-coil domain family 3 | 9,64E-09 | -3,09912 |
| CDCA3 | cell division cycle associated 3 | 0,001888 | -3,08631 |
| CGNL1 | cingulin-like 1 | 1,55E-10 | -3,08158 |
| HMGCS1 | 3-hydroxy-3-methylglutaryl-CoA synthase 1 (soluble) | 0,000902 | -3,08125 |
| CDT1 | Chromatin licensing and DNA replication factor 1 | 0,000121 | -3,07599 |
| DUT | deoxyuridine triphosphatase | 1,90E-10 | -3,07589 |
| SLC2A13 | solute carrier family 2 (facilitated glucose transporter), member 13 | 3,67E-07 | -3,07449 |
| HECW2 | HECT, C2 and WW domain containing E3 ubiquitin protein ligase 2 | 6,69E-06 | -3,06562 |
| IRAK3 | interleukin-1 receptor-associated kinase 3 | 8,46E-06 | -3,06147 |
| NPTX1 | neuronal pentraxin I | 8,16E-09 | -3,05538 |
| DNAJC12 | DnaJ (Hsp40) homolog, subfamily C, member 12 | 2,60E-12 | -3,05431 |
| SHCBP1 | SHC SH2-domain binding protein 1 | 0,006907 | -3,04857 |
| ABCC3 | ATP-binding cassette, sub-family C (CFTR/MRP), member 3 | 4,35E-10 | -3,04821 |
| NCAPG | non-SMC condensin I complex, subunit G | 0,000306 | -3,04366 |
| FAM83D | family with sequence similarity 83, member D | 0,004437 | -3,04291 |
| CCL8 | chemokine (C-C motif) ligand 8 | 0,000241 | -3,03965 |
| LAMA4 | laminin, alpha 4 | 0,000471 | -3,0371 |
| KIF14 | kinesin family member 14 | 1,71E-05 | -3,01314 |
| ZADH2 | zinc binding alcohol dehydrogenase domain containing 2 | 2,06E-09 | -3,01065 |
| C1orf38 | chromosome 1 open reading frame 38 | 3,89E-07 | -3,01033 |
| EFCAB7 | EF-hand calcium binding domain 7 | 3,21E-11 | -3,00967 |
| SETDB2 | SET domain, bifurcated 2 | 1,46E-08 | -3,00707 |
| MND1 | meiotic nuclear divisions 1 homolog (S. cerevisiae) | 0,007584 | -3,0029 |
| DTWD1 | DTW domain containing 1 | 3,84E-17 | -3,00269 |

Supplemental File 6. Table of Fold Change value from Microarray and qPCR analysis for each comparison.

| **Fold Change for** | **ADORA2B** | **CCL7** | **SFRP1** | **KYNU** | **ANKRD1** | **MMP1** | **LAMC2** | **G0S2** | **ALDH1A1** | **MAB21L1** | **NDN** | **YWHAZ** |
| --- | --- | --- | --- | --- | --- | --- | --- | --- | --- | --- | --- | --- |
| **Senescent hMSC/n vs Young hMSC/n** | | | | | | | | | | | | |
| Microarray | ND | ND | ND | ND | -11,2205 | 4,34315 | ND | ND | ND | ND | ND | 1 |
| qPCR | 0,816735 | 4,241349 | 0,953098 | 0,732824 | 0,092066 | 4,6585 | 1,139223 | 1,466246 | 0,103725 | 0,917549 | 2,039583 | 1 |
| P value |  |  |  |  | 0,003* | 0,0173* |  |  |  |  |  |  |
| **Senescent hMSC/inv vs Young hMSC/inv** | | | | | | | | | | | | |
| Microarray | ND | ND | -3,57804 | ND | -4,46141 | ND | ND | 6,36868 | ND | ND | 5,27179 | 1 |
| qPCR | 8,38413 | 1,84346 | 0,271548 | 0,575128 | 0,212635 | 0,621564 | 0,833506 | 45,71479 | 1,343754 | 0,399078 | 13,57535 | 1 |
| P value |  |  | 0,01* |  | <0.001* |  |  | 0,052* |  |  | 0,078* |  |
| **Young hMSC/inv vs Young hMSC/n** | | | | | | | | | | | | |
| Microarray | 4,2307 | 4,1251 | -19,1773 | 4,88697 | -8,77528 | -8,79661 | -14,4033 | 3,16497 | -26,1744 | 5,26257 | 6,29509 | 1 |
| qPCR | 0,561207 | 13,8286 | 0,095798 | 17,35176 | 0,396335 | 0,55391 | 0,05942 | 9,59623 | 0,139944 | 1,876331 | 2,820094 | 1 |
| P value | 0,029* | 0,099 | <0.001* | 0,023* | 0,8 | 0,23 | 0,021 | 0,048* | <0.001* | 0,001* | 0,015 |  |
| **Senescent hMSC/inv vs Senescent hMSC/n** | | | | | | | | | | | | |
| Microarray | 7,17501 | 8,538 | -39,0604 | 21,6892 | -13,4826 | -96,6301 | -17,3405 | 28,6178 | -56,2836 | 3,7473 | 17,2732 | 1 |
| qPCR | 8,0109 | 8,947832 | 0,023669 | 18,11707 | 0,117488 | 0,010657 | 0,010294 | 419,3468 | 4,269237 | 2,044939 | 15,15871 | 1 |
| P value | 0,001* | 0,036* | 0,003* | <0.001* | <0.001* | 0,003* | <0.001* | <0.001* | 0,258 | 0,01* | <0.001* |  |

* Statistical significance between the Fold Change value of Microarray and qPCR (T Test; p<0.05). ND = Gene not differentially expressed in the microarray analysis.

Supplemental File 7. Table with all functional categories of DEG in senescent hMSC/n compared to young hMSC/n.

| **Senescente hMSC/n vc Young hMSC/n** | |
| --- | --- |
| **Cellular and molecular function** | **Molecules Number** |
| Cell Growth and proliferation cell | 12 |
| Cell Death | 11 |
| Small molecule Biochemistry | 10 |
| Cellular Movement | 10 |
| Cellular Development | 10 |
| Cell to cell signaling and interation | 9 |
| Cell morphology | 8 |
| Embrionic development | 8 |
| Cell cycle | 7 |
| Carbohydrate Metabolism | 6 |
| Molecular Transport | 6 |
| Cellular Assembly and Organization | 4 |
| Conective Tissue Development and Function | 4 |
| Lipid Metabolism | 4 |
| Protein Syntesis | 4 |

*The categories with a p value ˂ 0.05 and with a biological representation ≥ 5% of molecules are represented.

Supplemental File 8. Table with functional annotation of the five most gene enriched categories with lower p-value of DEG in senescent hMSC/n compared to young hMSC/n.

| **senescent hMSC/n vs Young hMSC/n** | | | |
| --- | --- | --- | --- |
| **Functional Annotation** | **Molecules** | **Nº** | **P value** |
| **Small biochemical molecules (10 molecules)** | | |  |
| Synthesis of glycosaminoglycan | ANGPT1,GALNT5,HAS3,XYLT1 | 4 | 1,69E-05 |
| Accumulation of cyclic gmp | THBS1 | 1 | 4,61E-03 |
| Binding of heparan sulfate proteoglycan | THBS1 | 1 | 4,61E-03 |
| Formation of retinyl ester | RBP1 | 1 | 4,61E-03 |
| Production of l-triiodothyronine | DIO2 | 1 | 4,61E-03 |
| Storage of retinyl ester | RBP1 | 1 | 4,61E-03 |
| Synthesis of heparina | ANGPT1 | 1 | 4,61E-03 |
| Aggregation of hyaluronic acid | HAS3 | 1 | 9,19E-03 |
| Catabolism of androgen | HSD17B6 | 1 | 9,19E-03 |
| Metabolism of thyroid hormone | DIO2 | 1 | 9,19E-03 |
| Retention of hyaluronic acid | HAS3 | 1 | 9,19E-03 |
| Uptake of myristic acid | THBS1 | 1 | 9,19E-03 |
| Accumulation of cholesterol | FABP4 | 1 | 1,38E-02 |
| Import of l-glutamic acid | SLC1A1 | 1 | 1,38E-02 |
| Quantity of chondroitin sulfate | XYLT1 | 1 | 1,38E-02 |
| Modification of hyaluronic acid | THBS1 | 1 | 1,83E-02 |
| Metabolism of hormone | DIO2,HSD17B6 | 2 | 2,22E-02 |
| Accumulation of triacylglycerol | FABP4 | 1 | 2,28E-02 |
| Oxidation of dihydrotestosterone | HSD17B6 | 1 | 2,73E-02 |
| Uptake of l-glutamic acid | SLC1A1 | 1 | 2,73E-02 |
| Binding of heparina | THBS1 | 1 | 3,63E-02 |
| Synthesis of hyaluronic acid | HAS3 | 1 | 4,07E-02 |
| Metabolism of vitamin a | RBP1 | 1 | 4,51E-02 |
| Secretion of 5-hydroxytryptamine | THBS1 | 1 | 4,51E-02 |
| Synthesis of chondroitin sulfate | XYLT1 | 1 | 4,95E-02 |
| **Carbohydrate Metabolism (6 molecules)** | | |  |
| Synthesis of glycosaminoglycan | ANGPT1,GALNT5,HAS3,XYLT1 | 4 | 1,69E-05 |
| Synthesis of polysaccharide | ANGPT1,GALNT5,HAS3,IGFBP3,XYLT1 | 5 | 1,79E-05 |
| Binding of heparan sulfate proteoglycan | THBS1 | 1 | 4,61E-03 |
| Synthesis of heparina | ANGPT1 | 1 | 4,61E-03 |
| Aggregation of hyaluronic acid | HAS3 | 1 | 9,19E-03 |
| Retention of hyaluronic acid | HAS3 | 1 | 9,19E-03 |
| Quantity of chondroitin sulfate | XYLT1 | 1 | 1,38E-02 |
| Modification of hyaluronic acid | THBS1 | 1 | 1,83E-02 |
| Binding of heparina | THBS1 | 1 | 3,63E-02 |
| Synthesis of hyaluronic acid | HAS3 | 1 | 4,07E-02 |
| Synthesis of chondroitin sulfate | XYLT1 | 1 | 4,95E-02 |
| **Cell movement (10 molecules)** | | |  |
| Mobility of carcinoma cell lines | CADM1,SCUBE3 | 2 | 6,23E-05 |
| Mobility of lung cancer cell lines | CADM1,SCUBE3 | 2 | 6,23E-05 |
| Cell movement of endothelial cell lines | HOXD10,NTN4,THBS1 | 3 | 2,51E-03 |
| Cell flattening of t lymphocytes | THBS1 | 1 | 4,61E-03 |
| Invasion of mammary cells | MMP1 (includes EG:300339) | 1 | 4,61E-03 |
| Mobility of adenocarcinoma cell lines | SCUBE3 | 1 | 4,61E-03 |
| Mobilization of endothelial progenitor cells | ANGPT1 | 1 | 4,61E-03 |
| Recruitment of endothelial progenitor cells | ANGPT1 | 1 | 4,61E-03 |
| Recruitment of hematopoietic progenitor cells | ANGPT1 | 1 | 4,61E-03 |
| Migration of endothelial cells | ANGPT1,HAS3,IGFBP3,THBS1 | 4 | 7,87E-03 |
| Cell movement of skin cell lines | NTN4,THBS1 | 2 | 8,35E-03 |
| Chemotaxis of microvascular endothelial cells | THBS1 | 1 | 1,38E-02 |
| Migration of endothelial progenitor cells | IGFBP3 | 1 | 1,38E-02 |
| Migration of endothelial cell lines | HOXD10, NTN4 | 2 | 1,67E-02 |
| Migration of smooth muscle cells | IGFBP5,THBS1 | 2 | 1,74E-02 |
| Mobilization of hematopoietic progenitor cells | ANGPT1 | 1 | 2,28E-02 |
| Chemotaxis of smooth muscle cells | THBS1 | 1 | 3,18E-02 |
| Invasion of adenocarcinoma cell lines | SCUBE3 | 1 | 3,18E-02 |
| Chemotaxis of skin cell lines | THBS1 | 1 | 3,63E-02 |
| **Cell signaling and interaction (9 molecules)** | | |  |
| Binding of breast cancer cell lines | IGFBP3,OXTR,THBS1 | 3 | 1,16E-04 |
| Activation of tumor cell lines | CADM1,MMP1 (includes EG:300339),THBS1 | 3 | 2,51E-03 |
| Binding of tumor cell lines | HAS3,IGFBP3,OXTR,THBS1 | 4 | 4,31E-03 |
| Stimulation of tumor cell lines | IGFBP3,THBS1 | 2 | 4,54E-03 |
| Adhesion of streptococcus pyogenes | THBS1 | 1 | 4,61E-03 |
| Adhesion of squamous cell carcinoma cell lines | THBS1 | 1 | 4,61E-03 |
| Association of fibroblastos | IGFBP3 | 1 | 4,61E-03 |
| Recruitment of endothelial progenitor cells | ANGPT1 | 1 | 4,61E-03 |
| Recruitment of hematopoietic progenitor cells | ANGPT1 | 1 | 4,61E-03 |
| Stimulation of prostate cancer cell lines | IGFBP3 | 1 | 4,61E-03 |
| Synaptogenesis of retinal ganglion cells | THBS1 | 1 | 4,61E-03 |
| Adhesion of endothelial cell lines | NTN4,THBS1 | 2 | 4,96E-03 |
| Activation of breast cancer cell lines | MMP1 (includes EG:300339) | 1 | 9,19E-03 |
| Activation of lung cancer cell lines | CADM1 | 1 | 9,19E-03 |
| Binding of mammary tumor cells | IGFBP5 | 1 | 9,19E-03 |
| Stimulation of skin cancer cell lines | THBS1 | 1 | 9,19E-03 |
| Activation of carcinoma cell lines | CADM1 | 1 | 1,38E-02 |
| Phagocytosis of fibroblastos | THBS1 | 1 | 1,38E-02 |
| Binding of cells | HAS3,IGFBP3,IGFBP5,OXTR,THBS1 | 5 | 1,49E-02 |
| Activation of skin cancer cell lines | THBS1 | 1 | 1,83E-02 |
| Adhesion of melanoma cells | THBS1 | 1 | 1,83E-02 |
| Aggregation of kidney cell lines | CADM1 | 1 | 1,83E-02 |
| Activation of peripheral blood leukocytes | CADM1 | 1 | 1,18E-02 |
| dhesion of bone marrow cell lines | THBS1 | 1 | 3,18E-02 |
| Adhesion of skin cell lines | NTN4 | 1 | 3,63E-02 |
| Disassembly of focal adhesions | THBS1 | 1 | 4,07E-02 |
| Adhesion of microvascular endothelial cells | THBS1 | 1 | 4,51E-02 |
| Binding of fibroblastos | IGFBP3 | 1 | 4,51E-02 |
| Secretion of 5-hydroxytryptamine | THBS1 | 1 | 4,51E-02 |
| **Cell cycle (7 molecules)** | | | |
| Mitogenesis of tumor cell lines | IGFBP3,IGFBP5,THBS1 | 3 | 5,94E-04 |
| S phase of endothelial cells | ANGPT1 | 1 | 4,61E-03 |
| Endomitosis of monocytes | TNFRSF9 | 1 | 4,61E-03 |
| Mitogenesis of skin cancer cell lines | THBS1 | 1 | 9,19E-03 |
| Mitogenesis of cervical cancer cell lines | IGFBP5 | 1 | 1,83E-02 |
| Mitogenesis of prostate cancer cell lines | IGFBP3 | 1 | 2,73E-02 |
| Cell cycle progression of tumor cell lines | IGFBP3,KRT19 (human),RUNX1T1 | 3 | 4,73E-02 |
| Arrest in g2/m phase of breast cancer cell lines | IGFBP5 | 1 | 4,95E-02 |

Supplemental File 9. Table with all functional categories of DEG in senescent hMSC/inv compared to young hMSC/inv.

| **Senescent hMSC/inv vs Young hMSC/inv** | |
| --- | --- |
| **Cellular and molecular function** | **Molecules number** |
| Cellular Movement | 85 |
| Cell Growth and proliferation cell | 84 |
| Cell Death | 75 |
| Cellular Development | 74 |
| Conective Tissue Development and Function | 41 |
| Cell cycle | 38 |
| Cell to cell signaling and interation | 37 |
| Small molecule Biochemistry | 33 |
| Embrionic development | 30 |
| Cell morphology | 29 |
| Cellular Function and Maintenance | 27 |
| Cell signaling | 24 |
| Molecular Transport | 24 |
| Carbohydrate Metabolism | 21 |
| Protein Syntesis | 21 |
| Vitamin and Mineral Metabolism | 19 |
| Cellular Assembly and Organization | 18 |
| Lipid Metabolism | 18 |
| Gene Expression | 12 |
| DNA replication, recombination and repair | 11 |

The categories with a p value ˂ 0.05 and with a biological representation ≥ 5% of molecules are represented.

Supplemental File 10. Table with the functional annotation for the five most gene enriched categories with lower p-valueof DEG for senescent hMSC/inv compared to young hMSC/inv.

| **Functional Annotation** | **P value** | **Prediction of the activation state** | **Molecules** | **Molecules Number** |
| --- | --- | --- | --- | --- |
| **Proliferation of cell growth (84 molecules)** | | | | |
| Proliferation of cells | 2,68e-08 | Decreased | ACTB,ADAM12,AK4,ANGPTL1,BEX2,BHLHE40,CASP1,CCDC8,CCND2,CD274,CDCP1,CDKN1C,CHRM3,COL6A3,CREG1,CXADR,CXCL12 (INCLUDES EG:20315),CXCL2,CYP1B1,DAB2,DDIT3,DIRAS3,DLG1,DUSP5,EDN1,EGF (INCLUDES EG:13645),EHF,EIF4EBP1,ENO1,ENPP1,EREG,ETV1,F2RL1,F3,FBLN2,FGF1,FHL1 (INCLUDES EG:14199),FST,GDF15,GPNMB,GRP,HAS3,HBEGF,HGF,HMOX1,IGFBP5,KISS1,KLF4,LAMP3,MMP2,MYOCD,NDN,NOG,NRG1 (INCLUDES EG:112400),NRP1 (INCLUDES EG:18186),NTN4,NUPR1,OXTR,PDCD1LG2 (INCLUDES EG:309304),PLAT,PTPRG,RGS4,RGS5,RPS6KA2,RUNX1T1,RUNX2,SCUBE3,SERPINB2,SERPINE1,SFRP1,SLC2A1,SULT1E1,THBS1,TIMP3,TNFRSF9,TRPC4,TSLP,TXNIP,UNC5B,VDR,WARS,WNT16 | 82 |
| Proliferation of tumor cell lines | 2,23e-06 |  | BEX2,CCND2,CDCP1,CDKN1C,CHRM3,COL6A3,CREG1,CXADR,CXCL12 (INCLUDES EG:20315),CYP1B1,DAB2,DIRAS3,DUSP5,EDN1,EGF (INCLUDES EG:13645),EHF,EREG,ETV1,F2RL1,FBLN2,FGF1,FST,GDF15,GRP,HAS3,HBEGF,HGF,HMOX1,IGFBP5,KISS1,KLF4,MYOCD,NDN,NRG1 (INCLUDES EG:112400),NRP1 (INCLUDES EG:18186),OXTR,PLAT,PTPRG,RUNX1T1,RUNX2,SERPINB2,SERPINE1,SLC2A1,SULT1E1,THBS1,TIMP3,TXNIP,VDR | 48 |
| Proliferation of prostate cancer cell lines | 3,30e-07 |  | CXADR,DAB2,DIRAS3,EGF (INCLUDES EG:13645),EHF,FGF1,FST,GDF15,GRP,HAS3,HBEGF,HGF,HMOX1,IGFBP5,NRG1 (INCLUDES EG:112400),OXTR,VDR | 17 |
| Colony formation of cells | 3,91e-05 |  | ANKRD1,CXCL12 (INCLUDES EG:20315),DIRAS3,EDN1,EGF (INCLUDES EG:13645),EHF,EIF4EBP1,ENO1,EREG,HGF,KLF4,NDN,NRG1 (INCLUDES EG:112400),NUPR1,RUNX1T1,RUNX2,SFRP1 | 17 |
| Proliferation of breast cancer cell lines | 4,89e-04 |  | BEX2,CCND2,COL6A3,DAB2,EGF (INCLUDES EG:13645),FGF1,GDF15,HBEGF,HGF,IGFBP5,KISS1,NRG1 (INCLUDES EG:112400),NRP1 (INCLUDES EG:18186),PTPRG,SULT1E1,VDR | 16 |
| Proliferation of endothelial cells | 2,50e-07 |  | ANGPTL1,DAB2,DLG1,EDN1,EGF (INCLUDES EG:13645),F2RL1,F3,FGF1,HAS3,HGF,HMOX1,NRP1 (INCLUDES EG:18186),RGS5,RUNX2,THBS1 | 15 |
| Colony formation of tumor cell lines | 5,97e-04 |  | ANKRD1,DIRAS3,EDN1,EGF (INCLUDES EG:13645),EHF,ENO1,HGF,KLF4,NDN,NRG1 (INCLUDES EG:112400),NUPR1,RUNX2 | 12 |
| Proliferation of connective tissue cells | 2,97e-04 |  | CXCL12 (INCLUDES EG:20315),EDN1,EGF (INCLUDES EG:13645),EREG,FGF1,HGF,IGFBP5,NOG,SFRP1,VDR,WNT16 | 11 |
| Proliferation of muscle cells | 3,01e-06 | Decreased | EDN1,EREG,FHL1 (INCLUDES EG:14199),HBEGF,HGF,HMOX1,IGFBP5,NOG,SERPINE1,THBS1 | 10 |
| Proliferation of ovarian cancer cell lines | 3,01e-06 |  | CXCL12 (INCLUDES EG:20315),DAB2,DIRAS3,EDN1,EGF (INCLUDES EG:13645),FST,GDF15,HAS3,HBEGF,NRG1 (INCLUDES EG:112400) | 10 |
| Proliferation of epithelial cells | 5,64e-05 |  | CCND2,CDKN1C,EGF (INCLUDES EG:13645),EREG,HBEGF,HGF,RGS4,SFRP1,VDR,WNT16 | 10 |
| Proliferation of carcinoma cell lines | 1,13e-02 |  | CDCP1,CXCL12 (INCLUDES EG:20315),DAB2,EGF (INCLUDES EG:13645),FBLN2,GRP,HGF,HMOX1,KISS1,NRG1 (INCLUDES EG:112400) | 10 |
| Proliferation of smooth muscle cells | 9,19e-06 | Decreased | EDN1,EREG,FHL1 (INCLUDES EG:14199),HBEGF,HGF,HMOX1,IGFBP5,SERPINE1,THBS1 | 9 |
| Arrest in growth of cells | 1,43e-04 |  | CDKN1C,DAB2,EGF (INCLUDES EG:13645),FGF1,GDF15,HMOX1,KLF4,NRP1 (INCLUDES EG:18186),SERPINE1 | 9 |
| Proliferation of epithelial cell lines | 1,65e-04 |  | CCDC8,DAB2,DIRAS3,FGF1,HGF,OXTR,SCUBE3,TRPC4,UNC5B | 9 |
| Proliferation of tumor cells | 2,68e-03 |  | CASP1,DDIT3,EDN1,EGF (INCLUDES EG:13645),EIF4EBP1,FST,HGF,SFRP1,THBS1 | 9 |
| Proliferation of colon cancer cell lines | 1,11e-02 |  | DUSP5,EDN1,EGF (INCLUDES EG:13645),EREG,F2RL1,FST,GRP,KLF4,NRG1 (INCLUDES EG:112400) | 9 |
| Proliferation of kidney cell lines | 2,38e-04 |  | CCDC8,DIRAS3,EGF (INCLUDES EG:13645),FGF1,HGF,OXTR,SCUBE3,UNC5B | 8 |
| Proliferation of cancer cells | 3,59e-03 |  | CASP1,DDIT3,EDN1,EGF (INCLUDES EG:13645),EIF4EBP1,HGF,SFRP1,THBS1 | 8 |
| Proliferation of vascular endothelial cells | 2,30e-04 |  | DAB2,EGF (INCLUDES EG:13645),F2RL1,HGF,NRP1 (INCLUDES EG:18186),RGS5,THBS1 | 7 |
| Proliferation of embryonic cell lines | 3,54e-04 |  | CCDC8,DIRAS3,FGF1,HGF,OXTR,SCUBE3,UNC5B | 7 |
| Formation of cells | 8,71e-04 |  | ADAM12,EGF (INCLUDES EG:13645),IL33,NRG1 (INCLUDES EG:112400),RUNX2,SERPINE1,THBS1 | 7 |
| Proliferation of leukemia cell lines | 1,73e-02 |  | CDCP1,CDKN1C,HMOX1,NRG1 (INCLUDES EG:112400),RUNX2,SERPINB2,TXNIP | 7 |
| Arrest in growth of tumor cell lines | 3,09e-03 |  | CDKN1C,DAB2,GDF15,HMOX1,KLF4,SERPINE1 | 6 |
| Proliferation of keratinocytes | 1,53e-03 |  | EGF (INCLUDES EG:13645),EREG,HGF,VDR,WNT16 | 5 |
| Colony formation of breast cancer cell lines | 2,59e-03 | Decreased | DIRAS3,EGF (INCLUDES EG:13645),NRG1 (INCLUDES EG:112400),NUPR1,RUNX2 | 5 |
| Proliferation of endothelial cell lines | 6,58e-03 |  | CDKN1C,EGF (INCLUDES EG:13645),HGF,NTN4,THBS1 | 5 |
| Proliferation of skin cell lines | 5,43e-04 |  | EGF (INCLUDES EG:13645),HGF,NTN4,THBS1 | 4 |
| Proliferation of stomach cancer cell lines | 2,06e-03 |  | F2RL1,HBEGF,NRG1 (INCLUDES EG:112400),TXNIP | 4 |
| Expansion of cells | 1,51e-02 |  | CXCL12 (INCLUDES EG:20315),EGF (INCLUDES EG:13645),RUNX1T1,TNFRSF9 | 4 |
| Proliferation of sarcoma cell lines | 2,86e-04 |  | HGF,MYOCD,TIMP3 | 3 |
| Proliferation of lymphatic endothelial cells | 4,24e-04 |  | EDN1,FGF1,HGF | 3 |
| Proliferation of lung cancer cells | 5,98e-04 |  | DDIT3,EGF (INCLUDES EG:13645),THBS1 | 3 |
| Colony formation of cancer cells | 1,37e-03 |  | EIF4EBP1,HGF,SFRP1 | 3 |
| Proliferation of osteoblasts | 2,58e-03 |  | HGF,IGFBP5,SFRP1 | 3 |
| Proliferation of neuronal cells | 3,66e-03 |  | CXCL12 (INCLUDES EG:20315),EGF (INCLUDES EG:13645),HGF | 3 |
| Stimulation of tumor cell lines | 6,56e-03 |  | EGF (INCLUDES EG:13645),HGF,THBS1 | 3 |
| Proliferation of skin cancer cell lines | 7,45e-03 |  | EGF (INCLUDES EG:13645),HGF,THBS1 | 3 |
| Proliferation of sarcoma cells | 1,05e-02 |  | CASP1,EDN1,EGF (INCLUDES EG:13645) | 3 |
| Proliferation of bladder cancer cell lines | 1,70e-02 |  | CXADR,HBEGF,PLAT | 3 |
| Stimulation of skin cancer cell lines | 3,11e-04 |  | EGF (INCLUDES EG:13645),THBS1 | 2 |
| Colony formation of glioma cells | 1,83e-03 |  | HGF,SFRP1 | 2 |
| Proliferation of small cell lung cancer cells | 1,83e-03 |  | EGF (INCLUDES EG:13645),THBS1 | 2 |
| Formation of foam cells | 3,01e-03 |  | EGF (INCLUDES EG:13645),IL33 | 2 |
| Proliferation of neural stem cells | 3,01e-03 |  | CXCL12 (INCLUDES EG:20315),EGF (INCLUDES EG:13645) | 2 |
| Proliferation of embryonic cancer cell lines | 4,46e-03 |  | DAB2,EGF (INCLUDES EG:13645) | 2 |
| Proliferation of nervous tissue cell lines | 6,17e-03 |  | CXCL12 (INCLUDES EG:20315),NRP1 (INCLUDES EG:18186) | 2 |
| Proliferation of eye cell lines | 8,13e-03 |  | HGF,TRPC4 | 2 |
| Proliferation of brain cells | 1,03e-02 |  | CXCL12 (INCLUDES EG:20315),EGF (INCLUDES EG:13645) | 2 |
| Proliferation of hepatic stellate cells | 1,03e-02 |  | CXCL12 (INCLUDES EG:20315),EDN1 | 2 |
| Proliferation of leiomyoma cells | 1,03e-02 |  | EDN1,EGF (INCLUDES EG:13645) | 2 |
| Colony formation of melanoma cell lines | 1,28e-02 |  | EDN1,HGF | 2 |
| Arrest in growth of chondrocyte cell lines | 1,77e-02 |  | FGF1 | 1 |
| Arrest in growth of nervous tissue cell lines | 1,77e-02 |  | NRP1 (INCLUDES EG:18186) | 1 |
| **Cell death (75 molecules)** | | | | |
| Cell death | 6,04e-07 |  | ADAM12,ANKRD1,ASNS,ATP2B1,BEX2,BHLHE40,CACNA1A,CASP1,CCDC8,CD274,CDCP1,CDKN1C,CFLAR,CHI3L1,CXCL12 (INCLUDES EG:20315),CYP1B1,DAB2,DDIT3,DDIT4,DIRAS3,DUSP5,EDN1,EFEMP1,EGF (INCLUDES EG:13645),EHF,EIF4EBP1,ENO1,F2RL1,F3,FBXO32,FGF1,FST,G0S2,GDF15,HBEGF,HGF,HMOX1,IGFBP5,IL33,IRAK1,KISS1,KLF4,KRT19 (HUMAN),NDN,NRG1 (INCLUDES EG:112400),NRP1 (INCLUDES EG:18186),NTN4,NUPR1,OBFC2A,OLR1,PBX1,PLAT,PTGIS,PTPRG,RBP1,RGS4,RGS5,RPS6KA2,RUNX1T1,SERPINB2,SERPINE1,SFRP1,SLC22A3,SLC2A1,SNCAIP,ST6GAL1,THBS1,TIMP3,TNFRSF10D,TNFRSF9,TRIB3,UNC5B,VDR,WNT16 | 74 |
| Apoptosis | 1,71e-06 |  | ADAM12,ANKRD1,ASNS,BEX2,BHLHE40,CASP1,CCDC8,CD274,CDCP1,CDKN1C,CFLAR,CXCL12 (INCLUDES EG:20315),CYP1B1,DAB2,DDIT3,DDIT4,DIRAS3,EDN1,EFEMP1,EGF (INCLUDES EG:13645),EHF,EIF4EBP1,ENO1,F3,FBXO32,FGF1,FST,G0S2,GDF15,HBEGF,HGF,HMOX1,IGFBP5,KISS1,KLF4,NDN,NRG1 (INCLUDES EG:112400),NRP1 (INCLUDES EG:18186),NTN4,NUPR1,OLR1,PLAT,PTGIS,RGS4,RGS5,RPS6KA2,RUNX1T1,SERPINE1,SFRP1,ST6GAL1,THBS1,TIMP3,TNFRSF10D,TNFRSF9,TRIB3,UNC5B,WNT16 | 57 |
| Cell death of tumor cell lines | 3,86e-06 |  | ANKRD1,ASNS,ATP2B1,BEX2,BHLHE40,CASP1,CCDC8,CDCP1,CDKN1C,CFLAR,CXCL12 (INCLUDES EG:20315),CYP1B1,DAB2,DDIT3,DDIT4,DIRAS3,EDN1,EFEMP1,EGF (INCLUDES EG:13645),EHF,EIF4EBP1,ENO1,FBXO32,FST,G0S2,GDF15,HBEGF,HGF,HMOX1,IGFBP5,KISS1,KLF4,NDN,NRG1 (INCLUDES EG:112400),NRP1 (INCLUDES EG:18186),NUPR1,OBFC2A,RUNX1T1,SFRP1,ST6GAL1,THBS1,TIMP3,TNFRSF10D,UNC5B,VDR,WNT16 | 46 |
| Apoptosis of tumor cell lines | 1,74e-06 |  | ANKRD1,ASNS,BEX2,BHLHE40,CASP1,CCDC8,CDCP1,CDKN1C,CFLAR,CXCL12 (INCLUDES EG:20315),CYP1B1,DAB2,DDIT3,DDIT4,DIRAS3,EDN1,EFEMP1,EGF (INCLUDES EG:13645),EHF,EIF4EBP1,ENO1,FBXO32,FST,G0S2,GDF15,HBEGF,HGF,HMOX1,IGFBP5,KISS1,KLF4,NDN,NRG1 (INCLUDES EG:112400),NRP1 (INCLUDES EG:18186),NUPR1,RUNX1T1,SFRP1,ST6GAL1,TIMP3,TNFRSF10D,UNC5B,WNT16 | 42 |
| Cell survival | 7,91e-06 |  | BEX2,CASP1,CDKN1C,CFLAR,CHI3L1,CXCL12 (INCLUDES EG:20315),DAB2,DDIT3,DUSP5,EGF (INCLUDES EG:13645),FGF1,GDF15,HBEGF,HGF,HMOX1,IL33,IRAK1,KRT19 (HUMAN),NRG1 (INCLUDES EG:112400),NRP1 (INCLUDES EG:18186),NTN4,NUPR1,OLR1,PBX1,PTPRG,RBP1,RUNX1T1,SERPINB2,SFRP1,SLC22A3,SLC2A1,THBS1,TNFRSF9,VDR | 34 |
| Cell viability | 8,13e-06 |  | BEX2,CASP1,CDKN1C,CFLAR,CHI3L1,CXCL12 (INCLUDES EG:20315),DAB2,DDIT3,DUSP5,EGF (INCLUDES EG:13645),FGF1,GDF15,HBEGF,HGF,HMOX1,IL33,IRAK1,KRT19 (HUMAN),NRG1 (INCLUDES EG:112400),NRP1 (INCLUDES EG:18186),NTN4,NUPR1,OLR1,PBX1,PTPRG,RBP1,RUNX1T1,SERPINB2,SFRP1,SLC22A3,SLC2A1,THBS1,TNFRSF9 | 33 |
| Cell viability of tumor cell lines | 9,50e-03 |  | BEX2,CASP1,CDKN1C,CFLAR,CXCL12 (INCLUDES EG:20315),DUSP5,EGF (INCLUDES EG:13645),GDF15,HBEGF,HGF,HMOX1,IRAK1,KRT19 (HUMAN),NRG1 (INCLUDES EG:112400),NRP1 (INCLUDES EG:18186),NUPR1,PBX1,PTPRG,SERPINB2,SLC2A1 | 20 |
| Cell death of prostate cancer cell lines | 5,87e-05 |  | CASP1,CFLAR,DDIT3,DIRAS3,EDN1,EGF (INCLUDES EG:13645),EHF,FST,GDF15,HBEGF,HGF,IGFBP5 | 12 |
| Apoptosis of breast cancer cell lines | 1,56e-03 |  | BEX2,CASP1,CFLAR,DAB2,DDIT4,DIRAS3,EGF (INCLUDES EG:13645),FBXO32,HGF,IGFBP5,NRG1 (INCLUDES EG:112400),SFRP1 | 12 |
| Cell death of colon cancer cell lines | 1,56e-03 |  | CASP1,CDKN1C,CFLAR,DDIT3,EDN1,EGF (INCLUDES EG:13645),G0S2,GDF15,HMOX1,NRG1 (INCLUDES EG:112400),ST6GAL1,VDR | 12 |
| Cell death of epithelial cells | 2,54e-03 |  | CASP1,CFLAR,EGF (INCLUDES EG:13645),FGF1,HGF,NRG1 (INCLUDES EG:112400),PTGIS,RGS4,SFRP1,TIMP3,TRIB3,UNC5B | 12 |
| Cell death of kidney cell lines | 2,83e-03 |  | CASP1,CFLAR,EGF (INCLUDES EG:13645),F3,FGF1,HGF,NRG1 (INCLUDES EG:112400),PTGIS,RGS4,TRIB3,UNC5B | 11 |
| Apoptosis of prostate cancer cell lines | 3,71e-04 |  | CFLAR,DIRAS3,EDN1,EGF (INCLUDES EG:13645),EHF,FST,GDF15,HBEGF,HGF,IGFBP5 | 10 |
| Apoptosis of kidney cell lines | 8,52e-04 |  | CASP1,CFLAR,EGF (INCLUDES EG:13645),F3,FGF1,HGF,NRG1 (INCLUDES EG:112400),PTGIS,RGS4,TRIB3 | 10 |
| Apoptosis of colon cancer cell lines | 4,36e-03 |  | CASP1,CDKN1C,CFLAR,DDIT3,EDN1,EGF (INCLUDES EG:13645),G0S2,GDF15,NRG1 (INCLUDES EG:112400),ST6GAL1 | 10 |
| Cell death of epithelial cell lines | 5,83e-03 |  | CASP1,CFLAR,EGF (INCLUDES EG:13645),FGF1,HGF,NRG1 (INCLUDES EG:112400),PTGIS,RGS4,TRIB3,UNC5B | 10 |
| Cell death of endothelial cells | 4,20e-05 |  | CASP1,CFLAR,DDIT3,F2RL1,HGF,OLR1,PLAT,RGS5,THBS1 | 9 |
| Apoptosis of epithelial cell lines | 2,08e-03 |  | CASP1,CFLAR,EGF (INCLUDES EG:13645),FGF1,HGF,NRG1 (INCLUDES EG:112400),PTGIS,RGS4,TRIB3 | 9 |
| Cell death of tumor cells | 4,59e-03 |  | ADAM12,CASP1,CFLAR,CXCL12 (INCLUDES EG:20315),DDIT3,ENO1,HBEGF,HMOX1,SFRP1 | 9 |
| Cell death of embryonic cell lines | 1,40e-02 |  | CASP1,CFLAR,EGF (INCLUDES EG:13645),FGF1,NRG1 (INCLUDES EG:112400),PTGIS,RGS4,TRIB3,UNC5B | 9 |
| Apoptosis of endothelial cells | 1,62e-04 |  | CASP1,CFLAR,DDIT3,HGF,OLR1,PLAT,RGS5,THBS1 | 8 |
| Inhibition of apoptosis | 3,59e-03 |  | CFLAR,F3,HMOX1,NRG1 (INCLUDES EG:112400),SERPINB2,SOCS2,THBS1,TNFRSF10D | 8 |
| Apoptosis of embryonic cell lines | 5,86e-03 |  | CASP1,CFLAR,EGF (INCLUDES EG:13645),FGF1,NRG1 (INCLUDES EG:112400),PTGIS,RGS4,TRIB3 | 8 |
| Cell death of lung cancer cell lines | 1,43e-02 |  | CASP1,CDCP1,CDKN1C,CFLAR,DDIT3,EGF (INCLUDES EG:13645),G0S2,NRG1 (INCLUDES EG:112400) | 8 |
| Cell death of vascular endothelial cells | 1,31e-04 |  | CASP1,CFLAR,DDIT3,F2RL1,HGF,RGS5,THBS1 | 7 |
| Apoptosis of brain cancer cell lines | 8,13e-04 |  | CASP1,CDKN1C,CFLAR,EIF4EBP1,HGF,NRP1 (INCLUDES EG:18186),UNC5B | 7 |
| Apoptosis of tumor cells | 1,27e-02 |  | ADAM12,CASP1,CFLAR,CXCL12 (INCLUDES EG:20315),DDIT3,ENO1,HBEGF | 7 |
| Cell viability of blood cells | 4,66e-03 | Increased | CXCL12 (INCLUDES EG:20315),EGF (INCLUDES EG:13645),HGF,IL33,RUNX1T1,TNFRSF9 | 6 |
| Cell death of ovarian cancer cell lines | 7,45e-03 |  | CASP1,CFLAR,DAB2,DDIT3,DIRAS3,HGF | 6 |
| Cell viability of breast cancer cell lines | 8,21e-03 |  | BEX2,GDF15,HGF,KRT19 (HUMAN),NRG1 (INCLUDES EG:112400),PBX1 | 6 |
| Apoptosis of bone cancer cell lines | 1,45e-02 |  | BHLHE40,CCDC8,CFLAR,IGFBP5,NDN,NUPR1 | 6 |
| Cell viability of endothelial cells | 2,26e-04 |  | DAB2,DDIT3,FGF1,HGF,OLR1 | 5 |
| Apoptosis of vascular endothelial cells | 3,82e-03 |  | CASP1,DDIT3,HGF,RGS5,THBS1 | 5 |
| Cell viability of leukocytes | 1,30e-02 |  | CXCL12 (INCLUDES EG:20315),EGF (INCLUDES EG:13645),HGF,IL33,TNFRSF9 | 5 |
| Cell viability of phagocytes | 2,06e-03 |  | CXCL12 (INCLUDES EG:20315),HGF,IL33,TNFRSF9 | 4 |
| Cell viability of lymphoma cell lines | 4,39e-03 |  | CFLAR,HBEGF,HMOX1,IRAK1 | 4 |
| Cell viability of mononuclear leukocytes | 7,44e-03 |  | CXCL12 (INCLUDES EG:20315),EGF (INCLUDES EG:13645),HGF,TNFRSF9 | 4 |
| Apoptosis of muscle cells | 8,70e-03 |  | CASP1,EDN1,NRG1 (INCLUDES EG:112400),TIMP3 | 4 |
| Apoptosis of fibroblasts | 1,61e-02 |  | CFLAR,EGF (INCLUDES EG:13645),SFRP1,TIMP3 | 4 |
| Cell death of pancreatic cancer cell lines | 1,71e-02 |  | ASNS,DDIT3,EFEMP1,HGF | 4 |
| Cell viability of skin cell lines | 4,24e-04 |  | EGF (INCLUDES EG:13645),NTN4,THBS1 | 3 |
| Cell viability of endothelial cell lines | 1,72e-03 |  | EGF (INCLUDES EG:13645),NTN4,THBS1 | 3 |
| Survival of hematopoietic cells | 9,42e-03 |  | CXCL12 (INCLUDES EG:20315),EGF (INCLUDES EG:13645),RUNX1T1 | 3 |
| Apoptosis of dermal cells | 1,05e-02 |  | CFLAR,HGF,SFRP1 | 3 |
| Cell viability of monocytes | 3,01e-03 |  | HGF,TNFRSF9 | 2 |
| Apoptosis of dermal fibroblastos | 4,46e-03 |  | CFLAR,SFRP1 | 2 |
| Cell viability of vascular endothelial cells | 4,46e-03 |  | DAB2,DDIT3 | 2 |
| Apoptosis of peripheral blood leukocytes | 1,54e-02 |  | CD274,KLF4 | 2 |
| Apoptosis of hcaec cells | 1,77e-02 |  | OLR1 | 1 |
| Apoptosis of embryonic stem cells | 1,77e-02 |  | NRP1 (INCLUDES EG:18186) | 1 |
| **Cellular development (74 molecules)** | | | | |
| Proliferation of tumor cell lines | 2,23e-06 |  | BEX2,CCND2,CDCP1,CDKN1C,CHRM3,COL6A3,CREG1,CXADR,CXCL12 (INCLUDES EG:20315),CYP1B1,DAB2,DIRAS3,DUSP5,EDN1,EGF (INCLUDES EG:13645),EHF,EREG,ETV1,F2RL1,FBLN2,FGF1,FST,GDF15,GRP,HAS3,HBEGF,HGF,HMOX1,IGFBP5,KISS1,KLF4,MYOCD,NDN,NRG1 (INCLUDES EG:112400),NRP1 (INCLUDES EG:18186),OXTR,PLAT,PTPRG,RUNX1T1,RUNX2,SERPINB2,SERPINE1,SLC2A1,SULT1E1,THBS1,TIMP3,TXNIP,VDR | 48 |
| Differentiation of cells | 2,93e-06 |  | ANGPTL2,BHLHE40,CREG1,CXCL12 (INCLUDES EG:20315),EGF (INCLUDES EG:13645),ENO1,ENPP1,EREG,FGF1,FST,HBEGF,HGF,IGFBP5,KLF4,MYOCD,NOG,NRG1 (INCLUDES EG:112400),RGS4,RUNX1T1,RUNX2,SERPINB2,SFRP1,ST8SIA4,STMN2,TRIB3,TXNIP,UNC5B,VDR,WNT16 | 29 |
| Proliferation of prostate cancer cell lines | 3,30e-07 |  | CXADR,DAB2,DIRAS3,EGF (INCLUDES EG:13645),EHF,FGF1,FST,GDF15,GRP,HAS3,HBEGF,HGF,HMOX1,IGFBP5,NRG1 (INCLUDES EG:112400),OXTR,VDR | 17 |
| Endothelial cell development | 1,81e-07 |  | ANGPTL1,DAB2,DLG1,EDN1,EGF (INCLUDES EG:13645),F2RL1,F3,FGF1,HAS3,HGF,HMOX1,MMP2,NRP1 (INCLUDES EG:18186),RGS5,RUNX2,THBS1 | 16 |
| Proliferation of breast cancer cell lines | 4,89e-04 |  | BEX2,CCND2,COL6A3,DAB2,EGF (INCLUDES EG:13645),FGF1,GDF15,HBEGF,HGF,IGFBP5,KISS1,NRG1 (INCLUDES EG:112400),NRP1 (INCLUDES EG:18186),PTPRG,SULT1E1,VDR | 16 |
| Proliferation of endothelial cells | 2,50e-07 |  | ANGPTL1,DAB2,DLG1,EDN1,EGF (INCLUDES EG:13645),F2RL1,F3,FGF1,HAS3,HGF,HMOX1,NRP1 (INCLUDES EG:18186),RGS5,RUNX2,THBS1 | 15 |
| Differentiation of tumor cell lines | 3,16e-05 |  | BHLHE40,CREG1,ENO1,FST,HGF,IGFBP5,KLF4,NRG1 (INCLUDES EG:112400),RUNX1T1,RUNX2,SERPINB2,ST8SIA4,VDR | 13 |
| Proliferation of muscle cells | 3,01e-06 | DECREASED | EDN1,EREG,FHL1 (INCLUDES EG:14199),HBEGF,HGF,HMOX1,IGFBP5,NOG,SERPINE1,THBS1 | 10 |
| Proliferation of ovarian cancer cell lines | 3,01e-06 |  | CXCL12 (INCLUDES EG:20315),DAB2,DIRAS3,EDN1,EGF (INCLUDES EG:13645),FST,GDF15,HAS3,HBEGF,NRG1 (INCLUDES EG:112400) | 10 |
| Proliferation of carcinoma cell lines | 1,13e-02 |  | CDCP1,CXCL12 (INCLUDES EG:20315),DAB2,EGF (INCLUDES EG:13645),FBLN2,GRP,HGF,HMOX1,KISS1,NRG1 (INCLUDES EG:112400) | 10 |
| Proliferation of smooth muscle cells | 9,19e-06 | DECREASED | EDN1,EREG,FHL1 (INCLUDES EG:14199),HBEGF,HGF,HMOX1,IGFBP5,SERPINE1,THBS1 | 9 |
| Proliferation of epithelial cell lines | 1,65e-04 |  | CCDC8,DAB2,DIRAS3,FGF1,HGF,OXTR,SCUBE3,TRPC4,UNC5B | 9 |
| Proliferation of tumor cells | 2,68e-03 |  | CASP1,DDIT3,EDN1,EGF (INCLUDES EG:13645),EIF4EBP1,FST,HGF,SFRP1,THBS1 | 9 |
| Proliferation of colon cancer cell lines | 1,11e-02 |  | DUSP5,EDN1,EGF (INCLUDES EG:13645),EREG,F2RL1,FST,GRP,KLF4,NRG1 (INCLUDES EG:112400) | 9 |
| Proliferation of kidney cell lines | 2,38e-04 |  | CCDC8,DIRAS3,EGF (INCLUDES EG:13645),FGF1,HGF,OXTR,SCUBE3,UNC5B | 8 |
| Proliferation of cancer cells | 3,59e-03 |  | CASP1,DDIT3,EDN1,EGF (INCLUDES EG:13645),EIF4EBP1,HGF,SFRP1,THBS1 | 8 |
| Proliferation of vascular endothelial cells | 2,30e-04 |  | DAB2,EGF (INCLUDES EG:13645),F2RL1,HGF,NRP1 (INCLUDES EG:18186),RGS5,THBS1 | 7 |
| Proliferation of embryonic cell lines | 3,54e-04 |  | CCDC8,DIRAS3,FGF1,HGF,OXTR,SCUBE3,UNC5B | 7 |
| Proliferation of leukemia cell lines | 1,73e-02 |  | CDCP1,CDKN1C,HMOX1,NRG1 (INCLUDES EG:112400),RUNX2,SERPINB2,TXNIP | 7 |
| Arrest in growth of tumor cell lines | 3,09e-03 |  | CDKN1C,DAB2,GDF15,HMOX1,KLF4,SERPINE1 | 6 |
| Differentiation of leukemia cell lines | 8,21e-03 |  | CREG1,ENO1,KLF4,RUNX1T1,RUNX2,SERPINB2 | 6 |
| Tubulation of endothelial cells | 9,23e-04 |  | EDN1,EGF (INCLUDES EG:13645),HGF,OLR1,TRPC4 | 5 |
| Differentiation of keratinocytes | 1,03e-03 |  | EGF (INCLUDES EG:13645),EREG,TXNIP,VDR,WNT16 | 5 |
| Proliferation of keratinocytes | 1,53e-03 |  | EGF (INCLUDES EG:13645),EREG,HGF,VDR,WNT16 | 5 |
| Epithelial-mesenchymal transition | 3,04e-03 |  | EGF (INCLUDES EG:13645),HGF,NOG,NRP1 (INCLUDES EG:18186),SCUBE3 | 5 |
| Proliferation of endothelial cell lines | 6,58e-03 |  | CDKN1C,EGF (INCLUDES EG:13645),HGF,NTN4,THBS1 | 5 |
| Proliferation of skin cell lines | 5,43e-04 |  | EGF (INCLUDES EG:13645),HGF,NTN4,THBS1 | 4 |
| Differentiation of adipocytes | 2,06e-03 |  | ENPP1,FGF1,SFRP1,TRIB3 | 4 |
| Proliferation of stomach cancer cell lines | 2,06e-03 |  | F2RL1,HBEGF,NRG1 (INCLUDES EG:112400),TXNIP | 4 |
| Branching of cells | 4,39e-03 |  | ANGPTL2,HGF,RGS4,UNC5B | 4 |
| Proliferation of sarcoma cell lines | 2,86e-04 |  | HGF,MYOCD,TIMP3 | 3 |
| Proliferation of lymphatic endothelial cells | 4,24e-04 |  | EDN1,FGF1,HGF | 3 |
| Proliferation of lung cancer cells | 5,98e-04 |  | DDIT3,EGF (INCLUDES EG:13645),THBS1 | 3 |
| Colony formation of cancer cells | 1,37e-03 |  | EIF4EBP1,HGF,SFRP1 | 3 |
| Proliferation of osteoblasts | 2,58e-03 |  | HGF,IGFBP5,SFRP1 | 3 |
| Proliferation of neuronal cells | 3,66e-03 |  | CXCL12 (INCLUDES EG:20315),EGF (INCLUDES EG:13645),HGF | 3 |
| Proliferation of skin cancer cell lines | 7,45e-03 |  | EGF (INCLUDES EG:13645),HGF,THBS1 | 3 |
| Branching of endothelial cells | 9,42e-03 |  | ANGPTL2,RGS4,UNC5B | 3 |
| Proliferation of sarcoma cells | 1,05e-02 |  | CASP1,EDN1,EGF (INCLUDES EG:13645) | 3 |
| Epithelial-mesenchymal transition of tumor cell lines | 1,29e-02 |  | EGF (INCLUDES EG:13645),NRP1 (INCLUDES EG:18186),SCUBE3 | 3 |
| Tubulation of vascular endothelial cells | 1,29e-02 |  | EGF (INCLUDES EG:13645),HGF,TRPC4 | 3 |
| Proliferation of bladder cancer cell lines | 1,70e-02 |  | CXADR,HBEGF,PLAT | 3 |
| Tubulation of lymphatic endothelial cells | 9,23e-04 |  | EDN1,HGF | 2 |
| Colony formation of glioma cells | 1,83e-03 |  | HGF,SFRP1 | 2 |
| Proliferation of small cell lung cancer cells | 1,83e-03 |  | EGF (INCLUDES EG:13645),THBS1 | 2 |
| Proliferation of neural stem cells | 3,01e-03 |  | CXCL12 (INCLUDES EG:20315),EGF (INCLUDES EG:13645) | 2 |
| Proliferation of embryonic cancer cell lines | 4,46e-03 |  | DAB2,EGF (INCLUDES EG:13645) | 2 |
| Differentiation of rhabdomyosarcoma cell lines | 6,17e-03 |  | FST,ST8SIA4 | 2 |
| Differentiation of smooth muscle cells | 6,17e-03 |  | EREG,MYOCD | 2 |
| Proliferation of nervous tissue cell lines | 6,17e-03 |  | CXCL12 (INCLUDES EG:20315),NRP1 (INCLUDES EG:18186) | 2 |
| Proliferation of hepatic stellate cells | 1,03e-02 |  | CXCL12 (INCLUDES EG:20315),EDN1 | 2 |
| Proliferation of leiomyoma cells | 1,03e-02 |  | EDN1,EGF (INCLUDES EG:13645) | 2 |
| Arrest in differentiation of granulocytes | 1,77e-02 |  | RUNX1T1 | 1 |
| Arrest in differentiation of lymphoma cell lines | 1,77e-02 |  | RUNX1T1 | 1 |
| Arrest in growth of nervous tissue cell lines | 1,77e-02 |  | NRP1 (INCLUDES EG:18186) | 1 |
| **Cell Movement (55 molecules)** | | | | |
| Cell movement | 2,34e-08 |  | AGTR1,ANGPTL1,ARHGEF6,CDCP1,CHI3L1,CHRM3,CXADR,CXCL12 (INCLUDES EG:20315),CXCL2,DAB2,EDN1,EGF (INCLUDES EG:13645),EREG,F2RL1,F3,FGF1,FHL1 (INCLUDES EG:14199),GDF15,GRP,HAS3,HBEGF,HGF,HMOX1,IGFBP5,IL33,KAL1,KISS1,KLF4,KRT19 (HUMAN),L1CAM,LTBP2,MMP2,NPPB,NRG1 (INCLUDES EG:112400),NRP1 (INCLUDES EG:18186),NTN4,OLR1,RGS4,RUNX2,SERPINE1,SFRP1,SLC2A1,ST6GAL1,THBS1,TIMP3,WARS,WNT5B | 47 |
| Migration of cells | 7,99e-08 |  | ANGPTL1,ARHGEF6,CHI3L1,CHRM3,CXADR,CXCL12 (INCLUDES EG:20315),CXCL2,DAB2,EDN1,EGF (INCLUDES EG:13645),EREG,F2RL1,F3,FGF1,FHL1 (INCLUDES EG:14199),GRP,HAS3,HBEGF,HGF,HMOX1,IGFBP5,IL33,KISS1,KLF4,L1CAM,LTBP2,MMP2,NPPB,NRG1 (INCLUDES EG:112400),NRP1 (INCLUDES EG:18186),NTN4,OLR1,RGS4,RUNX2,SERPINE1,SFRP1,SLC2A1,ST6GAL1,THBS1,TIMP3,WARS,WNT5B | 42 |
| Invasion of cells | 8,58e-07 |  | CHI3L1,CXCL12 (INCLUDES EG:20315),CYP1B1,DAB2,EDN1,EGF (INCLUDES EG:13645),ETV1,FST,GDF15,GRP,HAS3,HBEGF,HGF,HMOX1,KISS1,KLF4,MMP16,MMP2,NRG1 (INCLUDES EG:112400),NRP1 (INCLUDES EG:18186),PLAT,RGS4,SCUBE3,SFRP1,SLC2A1,TIMP3,UNC5B | 27 |
| Cell movement of tumor cell lines | 5,75e-05 |  | CDCP1,CXCL12 (INCLUDES EG:20315),CXCL2,DAB2,EDN1,EGF (INCLUDES EG:13645),EREG,F2RL1,FGF1,GDF15,GRP,HAS3,HGF,KISS1,KLF4,KRT19 (HUMAN),L1CAM,LTBP2,MMP2,NRG1 (INCLUDES EG:112400),NRP1 (INCLUDES EG:18186),RUNX2,SERPINE1,SLC2A1,ST6GAL1,THBS1 | 26 |
| Invasion of tumor cell lines | 2,02e-06 |  | CXCL12 (INCLUDES EG:20315),CYP1B1,DAB2,EDN1,EGF (INCLUDES EG:13645),ETV1,GDF15,GRP,HAS3,HBEGF,HGF,HMOX1,KISS1,KLF4,MMP16,MMP2,NRG1 (INCLUDES EG:112400),NRP1 (INCLUDES EG:18186),PLAT,SCUBE3,SFRP1,SLC2A1,TIMP3,UNC5B | 24 |
| Migration of tumor cell lines | 2,50e-05 |  | CXCL12 (INCLUDES EG:20315),CXCL2,DAB2,EDN1,EGF (INCLUDES EG:13645),EREG,F2RL1,FGF1,GRP,HAS3,HGF,KISS1,KLF4,L1CAM,LTBP2,MMP2,NRG1 (INCLUDES EG:112400),NRP1 (INCLUDES EG:18186),RUNX2,SERPINE1,SLC2A1,ST6GAL1,THBS1 | 23 |
| Chemotaxis | 2,30e-04 |  | AGTR1,CXADR,CXCL12 (INCLUDES EG:20315),CXCL2,EGF (INCLUDES EG:13645),F2RL1,HBEGF,HGF,IL33,KAL1,KISS1,L1CAM,MMP2,NRP1 (INCLUDES EG:18186),SERPINE1,THBS1 | 16 |
| Homing of cells | 4,62e-04 |  | AGTR1,CXADR,CXCL12 (INCLUDES EG:20315),CXCL2,EGF (INCLUDES EG:13645),F2RL1,HBEGF,HGF,IL33,KISS1,L1CAM,MMP2,NRP1 (INCLUDES EG:18186),SERPINE1,THBS1 | 15 |
| Chemotaxis of cells | 1,15e-03 |  | AGTR1,CXADR,CXCL12 (INCLUDES EG:20315),CXCL2,EGF (INCLUDES EG:13645),F2RL1,HBEGF,HGF,IL33,KISS1,MMP2,NRP1 (INCLUDES EG:18186),SERPINE1,THBS1 | 14 |
| Migration of endothelial cells | 1,09e-05 |  | ANGPTL1,ARHGEF6,CXCL12 (INCLUDES EG:20315),EDN1,EGF (INCLUDES EG:13645),F2RL1,FGF1,HAS3,HGF,NRP1 (INCLUDES EG:18186),OLR1,SERPINE1,THBS1 | 13 |
| Leukocyte migration | 1,56e-02 |  | CXADR,CXCL12 (INCLUDES EG:20315),CXCL2,F2RL1,FGF1,HGF,HMOX1,IL33,NRG1 (INCLUDES EG:112400),SERPINE1,THBS1,TIMP3 | 12 |
| Cell movement of breast cancer cell lines | 3,96e-04 |  | CXCL12 (INCLUDES EG:20315),EGF (INCLUDES EG:13645),FGF1,HGF,KISS1,KRT19 (HUMAN),L1CAM,MMP2,NRG1 (INCLUDES EG:112400),NRP1 (INCLUDES EG:18186),SERPINE1 | 11 |
| Migration of breast cancer cell lines | 1,46e-03 |  | CXCL12 (INCLUDES EG:20315),EGF (INCLUDES EG:13645),FGF1,HGF,KISS1,MMP2,NRG1 (INCLUDES EG:112400),NRP1 (INCLUDES EG:18186),SERPINE1 | 9 |
| Migration of tumor cells | 1,07e-04 |  | CXCL12 (INCLUDES EG:20315),EGF (INCLUDES EG:13645),F3,HGF,L1CAM,MMP2,SFRP1 | 7 |
| Cell movement of carcinoma cell lines | 2,52e-04 |  | CXCL12 (INCLUDES EG:20315),EGF (INCLUDES EG:13645),GRP,HGF,KISS1,NRG1 (INCLUDES EG:112400),RUNX2 | 7 |
| Cell movement of colon cancer cell lines | 4,50e-04 |  | CDCP1,EGF (INCLUDES EG:13645),F2RL1,GRP,HGF,KLF4,ST6GAL1 | 7 |
| Cell movement of ovarian cancer cell lines | 2,13e-05 |  | EDN1,EGF (INCLUDES EG:13645),GDF15,HAS3,HGF,NRG1 (INCLUDES EG:112400) | 6 |
| Invasion of colon cancer cell lines | 2,96e-04 |  | GRP,HAS3,HGF,KLF4,NRP1 (INCLUDES EG:18186),UNC5B | 6 |
| Cell movement of smooth muscle cells | 3,29e-04 |  | FHL1 (INCLUDES EG:14199),HBEGF,HGF,IGFBP5,MMP2,THBS1 | 6 |
| Migration of colon cancer cell lines | 5,39e-04 |  | EGF (INCLUDES EG:13645),F2RL1,GRP,HGF,KLF4,ST6GAL1 | 6 |
| Cell movement of tumor cells | 5,92e-04 |  | CXCL12 (INCLUDES EG:20315),EGF (INCLUDES EG:13645),HGF,L1CAM,MMP2,SFRP1 | 6 |
| Migration of carcinoma cell lines | 5,92e-04 |  | EGF (INCLUDES EG:13645),GRP,HGF,KISS1,NRG1 (INCLUDES EG:112400),RUNX2 | 6 |
| Movement of vascular endothelial cells | 3,93e-03 |  | ARHGEF6,CXCL12 (INCLUDES EG:20315),EGF (INCLUDES EG:13645),F2RL1,HGF,THBS1 | 6 |
| Cell movement of neurons | 1,58e-05 |  | CXCL12 (INCLUDES EG:20315),EGF (INCLUDES EG:13645),HBEGF,HGF,L1CAM | 5 |
| Migration of ovarian cancer cell lines | 4,30e-05 |  | EDN1,EGF (INCLUDES EG:13645),HAS3,HGF,NRG1 (INCLUDES EG:112400) | 5 |
| Invasion of squamous cell carcinoma cell lines | 2,63e-04 |  | CXCL12 (INCLUDES EG:20315),EGF (INCLUDES EG:13645),GRP,HBEGF,HGF | 5 |
| Migration of cervical cancer cell lines | 1,53e-03 |  | CXCL12 (INCLUDES EG:20315),CXCL2,EGF (INCLUDES EG:13645),EREG,HGF | 5 |
| Cell movement of cancer cells | 1,68e-03 |  | CXCL12 (INCLUDES EG:20315),HGF,L1CAM,MMP2,SFRP1 | 5 |
| Cell movement of endothelial cell lines | 3,82e-03 | Decreased | EGF (INCLUDES EG:13645),HGF,NRG1 (INCLUDES EG:112400),NTN4,THBS1 | 5 |
| Cell movement of lung cancer cell lines | 4,41e-03 |  | CXCL12 (INCLUDES EG:20315),EGF (INCLUDES EG:13645),HGF,NRG1 (INCLUDES EG:112400),RUNX2 | 5 |
| Invasion of carcinoma cell lines | 7,00e-03 |  | EGF (INCLUDES EG:13645),GRP,HGF,SCUBE3,TIMP3 | 5 |
| Cell movement of embryonic cell lines | 1,11e-02 |  | AGTR1,CXCL12 (INCLUDES EG:20315),EGF (INCLUDES EG:13645),HGF,L1CAM | 5 |
| Migration of vascular endothelial cells | 1,50e-02 |  | ARHGEF6,CXCL12 (INCLUDES EG:20315),EGF (INCLUDES EG:13645),F2RL1,HGF | 5 |
| Migration of neurons | 8,31e-05 |  | EGF (INCLUDES EG:13645),HBEGF,HGF,L1CAM | 4 |
| Migration of lymphatic system cells | 3,00e-04 |  | CXCL12 (INCLUDES EG:20315),FGF1,HGF,NRG1 (INCLUDES EG:112400) | 4 |
| Homing of embryonic cell lines | 6,48e-04 |  | AGTR1,CXCL12 (INCLUDES EG:20315),EGF (INCLUDES EG:13645),L1CAM | 4 |
| Homing of epithelial cell lines | 6,48e-04 |  | AGTR1,CXCL12 (INCLUDES EG:20315),EGF (INCLUDES EG:13645),L1CAM | 4 |
| Homing of kidney cell lines | 1,05e-03 |  | AGTR1,CXCL12 (INCLUDES EG:20315),EGF (INCLUDES EG:13645),L1CAM | 4 |
| Invasion of ovarian cancer cell lines | 1,05e-03 |  | CXCL12 (INCLUDES EG:20315),EDN1,EGF (INCLUDES EG:13645),HGF | 4 |
| Migration of melanoma cell lines | 1,22e-03 |  | CXCL12 (INCLUDES EG:20315),L1CAM,LTBP2,NRG1 (INCLUDES EG:112400) | 4 |
| Invasion of pancreatic cancer cell lines | 1,40e-03 |  | EGF (INCLUDES EG:13645),HGF,NRP1 (INCLUDES EG:18186),PLAT | 4 |
| Cell movement of skin cell lines | 1,82e-03 |  | EGF (INCLUDES EG:13645),HGF,NTN4,THBS1 | 4 |
| Migration of hepatoma cell lines | 1,82e-03 |  | CXCL12 (INCLUDES EG:20315),EGF (INCLUDES EG:13645),HGF,SLC2A1 | 4 |
| Migration of endothelial cell lines | 6,86e-03 | Decreased | EGF (INCLUDES EG:13645),HGF,NRG1 (INCLUDES EG:112400),NTN4 | 4 |
| Migration of smooth muscle cells | 7,44e-03 | Decreased | FHL1 (INCLUDES EG:14199),HBEGF,IGFBP5,THBS1 | 4 |
| Cell movement of epithelial cells | 8,05e-03 |  | CHRM3,HBEGF,HGF,RGS4 | 4 |
| Migration of lung cancer cell lines | 9,38e-03 |  | EGF (INCLUDES EG:13645),HGF,NRG1 (INCLUDES EG:112400),RUNX2 | 4 |
| Cell movement of central nervous system cells | 8,11e-04 |  | CHI3L1,CXCL12 (INCLUDES EG:20315),HGF | 3 |
| Migration of neuroblastoma cell lines | 8,11e-04 |  | CXCL12 (INCLUDES EG:20315),HGF,SERPINE1 | 3 |
| Cell movement of glioma cells | 1,07e-03 |  | CXCL12 (INCLUDES EG:20315),HGF,SFRP1 | 3 |
| Mobility of cells | 1,07e-03 |  | NRG1 (INCLUDES EG:112400),SCUBE3,TM4SF1 | 3 |
| Invasion of cervical cancer cell lines | 2,12e-03 |  | CXCL12 (INCLUDES EG:20315),EGF (INCLUDES EG:13645),HGF | 3 |
| Migration of keratinocyte cancer cell lines | 2,58e-03 |  | EGF (INCLUDES EG:13645),HGF,SERPINE1 | 3 |
| Scattering of tumor cell lines | 2,58e-03 |  | EGF (INCLUDES EG:13645),HGF,L1CAM | 3 |
| Cell movement of lymphoma cell lines | 3,66e-03 |  | CXCL12 (INCLUDES EG:20315),EGF (INCLUDES EG:13645),HGF | 3 |
| Chemotaxis of embryonic cell lines | 4,98e-03 |  | AGTR1,CXCL12 (INCLUDES EG:20315),EGF (INCLUDES EG:13645) | 3 |
| Chemotaxis of epithelial cell lines | 4,98e-03 |  | AGTR1,CXCL12 (INCLUDES EG:20315),EGF (INCLUDES EG:13645) | 3 |
| Migration of skin cell lines | 6,56e-03 |  | EGF (INCLUDES EG:13645),HGF,NTN4 | 3 |
| Chemotaxis of endothelial cells | 7,45e-03 |  | CXCL12 (INCLUDES EG:20315),HGF,THBS1 | 3 |
| Chemotaxis of kidney cell lines | 7,45e-03 |  | AGTR1,CXCL12 (INCLUDES EG:20315),EGF (INCLUDES EG:13645) | 3 |
| Migration of fibrosarcoma cell lines | 1,56e-02 |  | DAB2,KISS1,SERPINE1 | 3 |
| Migration of epithelial cells | 1,70e-02 |  | CHRM3,HBEGF,RGS4 | 3 |
| Migration of haec cells | 3,11e-04 |  | CXCL12 (INCLUDES EG:20315),HGF | 2 |
| Scattering of stomach cancer cell lines | 3,11e-04 |  | EGF (INCLUDES EG:13645),HGF | 2 |
| Invasion of sarcoma cell lines | 9,23e-04 |  | HGF,TIMP3 | 2 |
| Migration of astrocytes | 9,23e-04 |  | CHI3L1,HGF | 2 |
| Mobility of lung cancer cell lines | 9,23e-04 |  | NRG1 (INCLUDES EG:112400),SCUBE3 | 2 |
| Chemotaxis of cervical cancer cell lines | 1,83e-03 |  | CXCL12 (INCLUDES EG:20315),EGF (INCLUDES EG:13645) | 2 |
| Migration of progenitor cells | 1,83e-03 |  | CXCL12 (INCLUDES EG:20315),HGF | 2 |
| Mobility of carcinoma cell lines | 3,01e-03 |  | NRG1 (INCLUDES EG:112400),SCUBE3 | 2 |
| Scattering of breast cancer cell lines | 4,46e-03 |  | HGF,L1CAM | 2 |
| Cell movement of brain cells | 6,17e-03 |  | CXCL12 (INCLUDES EG:20315),HGF | 2 |
| Chemotaxis of smooth muscle cells | 6,17e-03 |  | MMP2,THBS1 | 2 |
| Invasion of trophoblast cells | 6,17e-03 |  | EGF (INCLUDES EG:13645),FST | 2 |
| Migration of bone marrow precursor cells | 6,17e-03 |  | CXCL12 (INCLUDES EG:20315),NRG1 (INCLUDES EG:112400) | 2 |
| Migration of lymphatic endothelial cells | 6,17e-03 |  | FGF1,HGF | 2 |
| Invasion of thyroid tumor cell lines | 8,13e-03 |  | HGF,TIMP3 | 2 |
| Migration of stem cells | 8,13e-03 |  | CXCL12 (INCLUDES EG:20315),NRG1 (INCLUDES EG:112400) | 2 |
| Migration of thyroid tumor cell lines | 8,13e-03 |  | HGF,KISS1 | 2 |
| Migration of lymphoma cell lines | 1,03e-02 |  | CXCL12 (INCLUDES EG:20315),EGF (INCLUDES EG:13645) | 2 |
| Chemotaxis of vascular endothelial cells | 1,28e-02 |  | HGF,THBS1 | 2 |
| Cell movement of lung cell lines | 1,54e-02 |  | EGF (INCLUDES EG:13645),NRG1 (INCLUDES EG:112400) | 2 |
| **Cell-cell interaction and signaling (37 molecules)** | | | | |
| Binding of cells | 5,58e-05 |  | ANGPTL1,CXADR,CXCL12 (INCLUDES EG:20315),EGF (INCLUDES EG:13645),F3,FGF1,HAS3,HGF,IGFBP5,MMP2,NPPB,NRP1 (INCLUDES EG:18186),OLR1,OXTR,SERPINE1,ST6GAL1,THBS1 | 17 |
| Adhesion of tumor cell lines | 1,55e-06 |  | ADAM12,CD274,CDCP1,CXCL12 (INCLUDES EG:20315),CXCL2,DAB2,EGF (INCLUDES EG:13645),GRP,HAS3,HGF,L1CAM,NRG1 (INCLUDES EG:112400),SERPINB2,SERPINE1,ST6GAL1,THBS1 | 16 |
| Activation of cells | 6,83e-04 |  | CXCL12 (INCLUDES EG:20315),EDN1,EGF (INCLUDES EG:13645),F2RL1,F3,FGF1,HBEGF,HGF,IL33,MMP2,PAG1,PLAT,SOCS2,THBS1,TSLP | 15 |
| Binding of tumor cell lines | 1,08e-05 |  | CXADR,CXCL12 (INCLUDES EG:20315),EGF (INCLUDES EG:13645),F3,HAS3,HGF,MMP2,NRP1 (INCLUDES EG:18186),OXTR,SERPINE1,ST6GAL1,THBS1 | 12 |
| Binding of breast cancer cell lines | 1,25e-06 |  | EGF (INCLUDES EG:13645),HGF,MMP2,OXTR,SERPINE1,THBS1 | 6 |
| Binding of endothelial cells | 1,26e-03 |  | ANGPTL1,CXCL12 (INCLUDES EG:20315),FGF1,HAS3,NRP1 (INCLUDES EG:18186),SERPINE1 | 6 |
| Adhesion of endothelial cells | 5,20e-03 |  | CD274,CXCL12 (INCLUDES EG:20315),HGF,L1CAM,OLR1,THBS1 | 6 |
| Adhesion of breast cancer cell lines | 2,01e-03 |  | CDCP1,EGF (INCLUDES EG:13645),NRG1 (INCLUDES EG:112400),SERPINE1,THBS1 | 5 |
| Adhesion of vascular endothelial cells | 5,79e-03 |  | CD274,CXCL12 (INCLUDES EG:20315),HGF,L1CAM,THBS1 | 5 |
| Cell-cell adhesion | 8,88e-03 |  | CD274,CXADR,DAB2,HGF,TIMP3 | 5 |
| Adhesion of carcinoma cell lines | 4,22e-05 |  | ADAM12,CXCL12 (INCLUDES EG:20315),GRP,THBS1 | 4 |
| Activation of vascular endothelial cells | 1,47e-04 |  | F2RL1,F3,HBEGF,HGF | 4 |
| Binding of prostate cancer cell lines | 1,47e-04 | Decreased | EGF (INCLUDES EG:13645),F3,HAS3,NRP1 (INCLUDES EG:18186) | 4 |
| Adhesion of colon cancer cell lines | 3,60e-03 |  | CD274,EGF (INCLUDES EG:13645),GRP,ST6GAL1 | 4 |
| Cell-cell adhesion of tumor cell lines | 5,74e-03 |  | CD274,DAB2,HGF | 3 |
| Secretion of neurotransmitter | 5,74e-03 |  | RAB27B,SNCAIP,THBS1 | 3 |
| Stimulation of tumor cell lines | 6,56e-03 |  | EGF (INCLUDES EG:13645),HGF,THBS1 | 3 |
| Adhesion of tumor cells | 1,05e-02 |  | CXCL12 (INCLUDES EG:20315),F3,THBS1 | 3 |
| Contact repulsion | 3,11e-04 |  | L1CAM,NRP1 (INCLUDES EG:18186) | 2 |
| Stimulation of skin cancer cell lines | 3,11e-04 |  | EGF (INCLUDES EG:13645),THBS1 | 2 |
| Adhesion of muscle precursor cells | 9,23e-04 |  | ADAM12,SERPINE1 | 2 |
| Activation of skin cancer cell lines | 1,83e-03 |  | EGF (INCLUDES EG:13645),THBS1 | 2 |
| Priming of cells | 6,17e-03 |  | EGF (INCLUDES EG:13645),FGF1 | 2 |
| Disassembly of focal adhesions | 1,03e-02 |  | DAB2,THBS1 | 2 |
| Secretion of 5-hydroxytryptamine | 1,28e-02 |  | RAB27B,THBS1 | 2 |
| Signaling of tumor cell lines | 1,28e-02 |  | EGF (INCLUDES EG:13645),HGF | 2 |
| Activation of stomach cancer cell lines | 1,77e-02 |  | EGF (INCLUDES EG:13645) | 1 |
| Adhesion of streptococcus pyogenes | 1,77e-02 |  | THBS1 | 1 |
| Adhesion of basophils | 1,77e-02 |  | IL33 | 1 |
| Adhesion of endothelial progenitor cells | 1,77e-02 |  | OLR1 | 1 |
| Adhesion of small cell lung cancer cells | 1,77e-02 |  | CXCL12 (INCLUDES EG:20315) | 1 |
| Adhesion of squamous cell carcinoma cell lines | 1,77e-02 |  | THBS1 | 1 |
| Antibody-dependent cell-mediated cytotoxicity of ovarian cancer cell lines | 1,77e-02 |  | HBEGF | 1 |
| Attraction of axons | 1,77e-02 |  | L1CAM | 1 |
| Binding of adrenocortical cells | 1,77e-02 |  | NPPB | 1 |
| Binding of endocrine cell lines | 1,77e-02 |  | HGF | 1 |
| Binding of rhabdomyosarcoma cell lines | 1,77e-02 |  | CXCL12 (INCLUDES EG:20315) | 1 |
| Binding of thyroid tumor cell lines | 1,77e-02 |  | HGF | 1 |

Supplemental File 11. Table with the functional classification of the 30 common DEG in senescent compared to young hMSCs for both karyotypes.

| **Função celular e molecular** | **Moléculas** |
| --- | --- |
| Cell Growth and proliferation cell | 10 |
| Cellular Development | 10 |
| Cell Death | 9 |
| Embrionic development | 8 |
| Cell to cell signaling and interation | 6 |
| Conective Tissue Development and Function | 6 |
| Cellular Movement | 5 |
| Cell cycle | 5 |
| Small molecule Biochemistry | 5 |
| Protein Syntesis | 4 |
| Cell morphology | 4 |
| Cellular Function and Maintenance | 4 |
| Carbohydrate Metabolism | 3 |
| Cellular Assembly and Organization | 3 |
| Lipid Metabolism | 2 |
| Drug Metabolism | 2 |
| Cellular Compromise | 2 |

Supplemental File 12. Table with functional annotation for the five most gene enriched categories with lower p-value for the 30 common DEG in the senescent compared to young hMSCs of both karyotypes.

| **Functions annotation** | **P value** | **Molecules** | **Molecules number** |
| --- | --- | --- | --- |
| **Cell death** | | | |
| Cell death | 3,77E-02 | ANKRD1,IGFBP5,KRT19 (human),NTN4,RBP1,RUNX1T1,SERPINB2,THBS1,TNFRSF9 | 9 |
| Cell viability | 8,54E-04 | KRT19 (human),NTN4,RBP1,RUNX1T1,SERPINB2,THBS1,TNFRSF9 | 7 |
| Cell viability of skin cell lines | 1,46E-04 | NTN4,THBS1 | 2 |
| Cell viability of endothelial cell lines | 3,67E-04 | NTN4,THBS1 | 2 |
| Cell viability of blood cells | 1,41E-02 | RUNX1T1,TNFRSF9 | 2 |
| Initiation of apoptosis | 2,64E-02 | THBS1,TNFRSF9 | 2 |
| Inhibition of apoptosis | 3,31E-02 | SERPINB2,THBS1 | 2 |
| Delay in apoptosis of lymphoma cell lines | 4,12E-03 | RUNX1T1 | 1 |
| Cell viability of monocytes | 1,03E-02 | TNFRSF9 | 1 |
| Cell viability of breast cell lines | 1,64E-02 | RBP1 | 1 |
| Permeability of endothelial cell lines | 2,85E-02 | NTN4 | 1 |
| Apoptosis of microvascular endothelial cells | 4,45E-02 | THBS1 | 1 |
| **Cell-to-cell signaling and interaction** | | | |
| Binding of cells | 3,76E-03 | HAS3,IGFBP5,OXTR,THBS1 | 4 |
| Binding of tumor cell lines | 3,14E-03 | HAS3,OXTR,THBS1 | 3 |
| Adhesion of tumor cell lines | 9,79E-03 | HAS3,SERPINB2,THBS1 | 3 |
| Binding of breast cancer cell lines | 8,40E-04 | OXTR,THBS1 | 2 |
| Adhesion of endothelial cell lines | 1,01E-03 | NTN4,THBS1 | 2 |
| Adhesion of streptococcus pyogenes | 2,06E-03 | THBS1 | 1 |
| Adhesion of squamous cell carcinoma cell lines | 2,06E-03 | THBS1 | 1 |
| Synaptogenesis of retinal ganglion cells | 2,06E-03 | THBS1 | 1 |
| Binding of mammary tumor cells | 4,12E-03 | IGFBP5 | 1 |
| Stimulation of skin cancer cell lines | 4,12E-03 | THBS1 | 1 |
| Phagocytosis of fibroblastos | 6,18E-03 | THBS1 | 1 |
| Activation of skin cancer cell lines | 8,23E-03 | THBS1 | 1 |
| Adhesion of melanoma cells | 8,23E-03 | THBS1 | 1 |
| Adhesion of bone marrow cell lines | 1,44E-02 | THBS1 | 1 |
| Adhesion of skin cell lines | 1,64E-02 | NTN4 | 1 |
| Disassembly of focal adhesions | 1,84E-02 | THBS1 | 1 |
| Adhesion of microvascular endothelial cells | 2,05E-02 | THBS1 | 1 |
| Secretion of 5-hydroxytryptamine | 2,05E-02 | THBS1 | 1 |
| Adhesion of carcinoma cell lines | 2,45E-02 | THBS1 | 1 |
| Adhesion of lung cancer cell lines | 2,65E-02 | THBS1 | 1 |
| Binding of prostate cancer cell lines | 3,25E-02 | HAS3 | 1 |
| Activation of macrophages | 4,65E-02 | THBS1 | 1 |
| Activation of blood platelets | 4,84E-02 | THBS1 | 1 |
| Adhesion of prostate cancer cell lines | 4,84E-02 | HAS3 | 1 |
| **Cellular development** | | | |
| Proliferation of tumor cell lines | 3,83E-02 | HAS3,IGFBP5,OXTR,RUNX1T1,SERPINB2,THBS1 | 6 |
| Differentiation of cells | 3,60E-02 | IGFBP5,RUNX1T1,SERPINB2,STMN2 | 4 |
| Differentiation of tumor cell lines | 6,23E-03 | IGFBP5,RUNX1T1,SERPINB2 | 3 |
| Proliferation of prostate cancer cell lines | 8,16E-03 | HAS3,IGFBP5,OXTR | 3 |
| Proliferation of blood cells | 1,79E-02 | RUNX1T1,THBS1,TNFRSF9 | 3 |
| Proliferation of skin cell lines | 9,23E-04 | NTN4,THBS1 | 2 |
| Expansion of blood cells | 3,51E-03 | RUNX1T1,TNFRSF9 | 2 |
| Proliferation of endothelial cell lines | 8,11E-03 | NTN4,THBS1 | 2 |
| Proliferation of embryonic cell lines | 9,84E-03 | OXTR,SCUBE3 | 2 |
| Proliferation of smooth muscle cells | 1,06E-02 | IGFBP5,THBS1 | 2 |
| Proliferation of kidney cell lines | 1,50E-02 | OXTR,SCUBE3 | 2 |
| Differentiation of leukemia cell lines | 1,72E-02 | RUNX1T1,SERPINB2 | 2 |
| Proliferation of epithelial cell lines | 2,11E-02 | OXTR,SCUBE3 | 2 |
| Proliferation of endothelial cells | 4,42E-02 | HAS3,THBS1 | 2 |
| Proliferation of t lymphocytes | 4,61E-02 | THBS1,TNFRSF9 | 2 |
| Arrest in differentiation of granulocytes | 2,06E-03 | RUNX1T1 | 1 |
| Arrest in differentiation of lymphoma cell lines | 2,06E-03 | RUNX1T1 | 1 |
| Synaptogenesis of retinal ganglion cells | 2,06E-03 | THBS1 | 1 |
| Expansion of erythroid cells | 4,12E-03 | RUNX1T1 | 1 |
| Proliferation of small cell lung cancer cells | 8,23E-03 | THBS1 | 1 |
| Epithelial-mesenchymal transition of lung cancer cell lines | 1,03E-02 | SCUBE3 | 1 |
| Epithelial-mesenchymal transition of carcinoma cell lines | 1,23E-02 | SCUBE3 | 1 |
| Proliferation of monocytes | 1,23E-02 | TNFRSF9 | 1 |
| Differentiation of myeloid progenitor cells | 1,64E-02 | RUNX1T1 | 1 |
| Proliferation of osteoblastos | 3,25E-02 | IGFBP5 | 1 |
| Differentiation of neuroblastoma cell lines | 4,05E-02 | IGFBP5 | 1 |
| Proliferation of skin cancer cell lines | 4,65E-02 | THBS1 | 1 |
| **Cellular growth and proliferation** | | | |
| Proliferation of tumor cell lines | 3,83E-02 | HAS3,IGFBP5,OXTR,RUNX1T1,SERPINB2,THBS1 | 6 |
| Proliferation of prostate cancer cell lines | 8,16E-03 | HAS3,IGFBP5,OXTR | 3 |
| Proliferation of blood cells | 1,79E-02 | RUNX1T1,THBS1,TNFRSF9 | 3 |
| Proliferation of skin cell lines | 9,23E-04 | NTN4,THBS1 | 2 |
| Expansion of blood cells | 3,51E-03 | RUNX1T1,TNFRSF9 | 2 |
| Proliferation of endothelial cell lines | 8,11E-03 | NTN4,THBS1 | 2 |
| Proliferation of embryonic cell lines | 9,84E-03 | OXTR,SCUBE3 | 2 |
| Proliferation of smooth muscle cells | 1,06E-02 | IGFBP5,THBS1 | 2 |
| Proliferation of kidney cell lines | 1,50E-02 | OXTR,SCUBE3 | 2 |
| Proliferation of epithelial cell lines | 2,11E-02 | OXTR,SCUBE3 | 2 |
| Proliferation of endothelial cells | 4,42E-02 | HAS3,THBS1 | 2 |
| Proliferation of t lymphocytes | 4,61E-02 | THBS1,TNFRSF9 | 2 |
| Expansion of erythroid cells | 4,12E-03 | RUNX1T1 | 1 |
| Formation of thyroid cells | 4,12E-03 | THBS1 | 1 |
| Stimulation of skin cancer cell lines | 4,12E-03 | THBS1 | 1 |
| Proliferation of small cell lung cancer cells | 8,23E-03 | THBS1 | 1 |
| Proliferation of monocytes | 1,23E-02 | TNFRSF9 | 1 |
| Colony formation of erythroid cells | 1,44E-02 | RUNX1T1 | 1 |
| Colony formation of hepatoma cell lines | 1,64E-02 | ANKRD1 | 1 |
| Proliferation of osteoblastos | 3,25E-02 | IGFBP5 | 1 |
| Proliferation of skin cancer cell lines | 4,65E-02 | THBS1 | 1 |
| **Cellular movement** | | | |
| Cell movement of skin cell lines | 1,72E-03 | NTN4,THBS1 | 2 |
| Migration of smooth muscle cells | 3,68E-03 | IGFBP5,THBS1 | 2 |
| Cell movement of endothelial cell lines | 6,52E-03 | NTN4,THBS1 | 2 |
| Migration of endothelial cells | 4,81E-02 | HAS3,THBS1 | 2 |
| Cell flattening of t lymphocytes | 2,06E-03 | THBS1 | 1 |
| Mobility of adenocarcinoma cell lines | 2,06E-03 | SCUBE3 | 1 |
| Chemotaxis of microvascular endothelial cells | 6,18E-03 | THBS1 | 1 |
| Mobility of carcinoma cell lines | 6,18E-03 | SCUBE3 | 1 |
| Mobility of lung cancer cell lines | 6,18E-03 | SCUBE3 | 1 |
| Chemotaxis of smooth muscle cells | 1,44E-02 | THBS1 | 1 |
| Invasion of adenocarcinoma cell lines | 1,44E-02 | SCUBE3 | 1 |
| Chemotaxis of skin cell lines | 1,64E-02 | THBS1 | 1 |
| Migration of fibroblasts | 2,45E-02 | THBS1 | 1 |
| Chemotaxis of endothelial cell lines | 2,65E-02 | THBS1 | 1 |
| Migration of ovarian cancer cell lines | 4,25E-02 | HAS3 | 1 |
| Migration of skin cell lines | 4,45E-02 | NTN4 | 1 |

Supplemental File 13. Table with all the functional categories of DEG in young hMSC/inv compared to young hMSc/n.

| **hMSC/inv Young vs hMSC/n Young** | |
| --- | --- |
| Functional categories ***** | **Nº of DEG** |
| Cellular Growth and Proliferation | 27 |
| Cellular Development | 21 |
| Cellular Movement | 18 |
| Cell Death | 17 |
| Cel to cell Signaling and interaction | 15 |
| Embryonic Development | 12 |
| Cell Signaling | 10 |
| Vitamin and Mineral Metabolism | 9 |
| Cellular Function and Maintenance | 8 |
| Small Molecule Biochemistry | 8 |
| Cell Morphology | 7 |
| Cellular Assembly and Organization | 7 |
| Antigen presentation | 6 |
| Lipid Metabolism | 6 |
| Molecular transport | 6 |
| Carbohydrate Metabolism | 5 |
| Cellular compromise | 4 |
|  |  |

*Functional categories that p value ˂0,05 and representation ≥ 5% of DEG

Supplemental File 14. Table with all functional categories of DEG in senescent hMSC/inv compared to hMSC/n.

| **Senescent hMSC/inv vs Senescent hMSC/n** | |
| --- | --- |
| **Categoria funcional** | **Nº of DEG** |
| Cellular Growth and Proliferation | 104 |
| Cell Death | 92 |
| Cellular Development | 86 |
| Cellular Movement | 70 |
| Cell Cycle | 55 |
| Cellular Assembly and Organization | 47 |
| DNA replication, recombinatin and repair | 45 |
| Small Molecule Biochemistry | 41 |
| Cel-To-cell Signaling and interacion | 34 |
| Embryonic Development | 30 |
| Carbohydrate Metabolism | 29 |
| Lipid Metabolism | 26 |
| Cell Morphology | 24 |
| Molecular Transport | 20 |
| Cellular Function and Maintenance | 17 |
| Cell Signaling | 15 |

*Functional categories that p value ˂0,05 and representation of ≥ 5% of DEG

Supplemental File 15. Table with functional annotation of the five most gene enriched categories with lower p-value of DEG in young hMSC/inv compared to young hMSC/n.

| **Functional Annotation** | **P value** | **Molecules** | **Number of de Molecules** |
| --- | --- | --- | --- |
| **Cell Movement (21 molecules)** | | | |
| Migration of cells | 1,67E-02 | CCL20,CCL7,CXADR,CXCL12 (includes EG:20315),DCBLD2,DOCK4,IGF2,LAMC2,NREP,NTN4,SFRP1,SPP1 (includes EG:20750) | 12 |
| Invasion of cells | 1,31E-02 | CTSK,CXCL12 (includes EG:20315),IGF2,MMP1 (includes EG:300339),PLAT,SCUBE3,SFRP1,SPP1 (includes EG:20750) | 8 |
| Cell movement of tumor cell lines | 3,81E-02 | CCL20,CCL7,CXCL12 (includes EG:20315),DCBLD2,IGF2,LAMC2,NREP,SPP1 (includes EG:20750) | 8 |
| Invasion of tumor cell lines | 1,78E-02 | CTSK,CXCL12 (includes EG:20315),MMP1 (includes EG:300339),PLAT,SCUBE3,SFRP1,SPP1 (includes EG:20750) | 7 |
| Migration of tumor cell lines | 2,98E-02 | CCL20,CXCL12 (includes EG:20315),DCBLD2,IGF2,LAMC2,NREP,SPP1 (includes EG:20750) | 7 |
| Chemotaxis of phagocytes | 2,56E-03 | CCL20,CCL7,CXADR,CXCL12 (includes EG:20315),SPP1 (includes EG:20750) | 5 |
| Cell movement of myeloid cells | 9,75E-03 | CCL20,CCL7,CXADR,CXCL12 (includes EG:20315),SPP1 (includes EG:20750) | 5 |
| Chemotaxis of neutrophils | 1,01E-03 | CCL7,CXADR,CXCL12 (includes EG:20315),SPP1 (includes EG:20750) | 4 |
| Cell movement of embryonic cell lines | 1,37E-03 | CCL20,CXCL12 (includes EG:20315),DOCK4,IGF2 | 4 |
| Chemotaxis of mononuclear leukocytes | 7,10E-03 | CCL20,CCL7,CXCL12 (includes EG:20315),SPP1 (includes EG:20750) | 4 |
| Migration of mononuclear leukocytes | 1,59E-02 | CCL20,CCL7,CXCL12 (includes EG:20315),SPP1 (includes EG:20750) | 4 |
| Cell movement of glioma cells | 3,93E-05 | CXCL12 (includes EG:20315),SFRP1,SPP1 (includes EG:20750) | 3 |
| Invasion of hepatoma cell lines | 5,77E-04 | CXCL12 (includes EG:20315),MMP1 (includes EG:300339),SPP1 (includes EG:20750) | 3 |
| Cell movement of pbmcs | 8,00E-04 | CCL20,CCL7,CXCL12 (includes EG:20315) | 3 |
| Chemotaxis of dendritic cells | 1,40E-03 | CCL20,CCL7,CXCL12 (includes EG:20315) | 3 |
| Migration of dendritic cells | 1,40E-03 | CCL20,CCL7,CXCL12 (includes EG:20315) | 3 |
| Adhesion of t lymphocytes | 2,21E-03 | CCL20,CXCL12 (includes EG:20315),SPP1 (includes EG:20750) | 3 |
| Chemotaxis of t lymphocytes | 3,68E-03 | CCL20,CXCL12 (includes EG:20315),SPP1 (includes EG:20750) | 3 |
| Migration of monocytes | 3,90E-03 | CCL20,CCL7,CXCL12 (includes EG:20315) | 3 |
| Migration of embryonic cell lines | 5,92E-03 | CCL20,DOCK4,IGF2 | 3 |
| Cell movement of epithelial cell lines | 9,57E-03 | CCL20,CXCL12 (includes EG:20315),DOCK4 | 3 |
| Cell movement of kidney cell lines | 1,84E-02 | CCL20,CXCL12 (includes EG:20315),DOCK4 | 3 |
| Transmigration of peripheral blood leukocytes | 2,37E-04 | CCL7,CXCL12 (includes EG:20315) | 2 |
| Cell movement of rhabdomyosarcoma cell lines | 5,88E-04 | CXCL12 (includes EG:20315),IGF2 | 2 |
| Transmigration of dendritic cells | 1,09E-03 | CCL7,CXCL12 (includes EG:20315) | 2 |
| Migration of lymphoma cell lines | 1,39E-03 | CXCL12 (includes EG:20315),LAMC2 | 2 |
| Mobility of cells | 1,39E-03 | SCUBE3,TM4SF1 | 2 |
| Chemotaxis of b lymphocytes | 2,52E-03 | CCL20,CXCL12 (includes EG:20315) | 2 |
| Migration of pbmcs | 2,52E-03 | CCL7,CXCL12 (includes EG:20315) | 2 |
| Cell movement of embryonic cells | 3,97E-03 | CXCL12 (includes EG:20315),IGF2 | 2 |
| Chemotaxis of pbmcs | 3,97E-03 | CCL20,CXCL12 (includes EG:20315) | 2 |
| Migration of hematopoietic progenitor cells | 7,74E-03 | CCL20,CXCL12 (includes EG:20315) | 2 |
| Recruitment of leukocytes | 7,74E-03 | CCL7,CXCL12 (includes EG:20315) | 2 |
| Migration of hepatoma cell lines | 1,17E-02 | CXCL12 (includes EG:20315),IGF2 | 2 |
| Invasion of squamous cell carcinoma cell lines | 1,64E-02 | CXCL12 (includes EG:20315),SPP1 (includes EG:20750) | 2 |
| Transmigration of mononuclear leukocytes | 2,07E-02 | CCL7,CXCL12 (includes EG:20315) | 2 |
| Cell movement of eosinophils | 2,78E-02 | CCL7,CXCL12 (includes EG:20315) | 2 |
| Migration of cervical cancer cell lines | 3,72E-02 | CXCL12 (includes EG:20315),DCBLD2 | 2 |
| Migration of epithelial cell lines | 3,72E-02 | CCL20,DOCK4 | 2 |
| Chemotaxis of leukemia cell lines | 4,16E-02 | CCL7,CXCL12 (includes EG:20315) | 2 |
| Invasion of prostate cancer cell lines | 4,76E-02 | MMP1 (includes EG:300339),SPP1 (includes EG:20750) | 2 |
| Migration of brain cancer cell lines | 4,76E-02 | CXCL12 (includes EG:20315),NREP | 2 |
| Migration of colon cancer cell lines | 4,92E-02 | CCL20,IGF2 | 2 |
| Adhesion of th17 cells | 6,35E-03 | CCL20 | 1 |
| Chemotaxis of acute lymphoblastic leukemia cells | 6,35E-03 | CXCL12 (includes EG:20315) | 1 |
| Chemotaxis of glioma cells | 6,35E-03 | CXCL12 (includes EG:20315) | 1 |
| Chemotaxis of granule cell precursors | 6,35E-03 | CXCL12 (includes EG:20315) | 1 |
| Chemotaxis of megakaryocytes | 6,35E-03 | CXCL12 (includes EG:20315) | 1 |
| Invasion of mammary cells | 6,35E-03 | MMP1 (includes EG:300339) | 1 |
| Invasion of smooth muscle cells | 6,35E-03 | SPP1 (includes EG:20750) | 1 |
| Locomotion of rhabdomyosarcoma cell lines | 6,35E-03 | CXCL12 (includes EG:20315) | 1 |
| Migration of th17 cells | 6,35E-03 | CCL20 | 1 |
| Migration of blood-derived mast cells | 6,35E-03 | CXCL12 (includes EG:20315) | 1 |
| Mobility of adenocarcinoma cell lines | 6,35E-03 | SCUBE3 | 1 |
| Transmigration of neural stem cells | 6,35E-03 | CXCL12 (includes EG:20315) | 1 |
| Chemoattraction of langerhans cells | 1,27E-02 | CCL20 | 1 |
| Chemotaxis of th1 cells | 1,27E-02 | CXCL12 (includes EG:20315) | 1 |
| Chemotaxis of th2 cells | 1,27E-02 | CXCL12 (includes EG:20315) | 1 |
| Chemotaxis of bone marrow cell lines | 1,27E-02 | CXCL12 (includes EG:20315) | 1 |
| Chemotaxis of natural killer t lymphocytes | 1,27E-02 | CCL20 | 1 |
| Chemotaxis of plasmacytoid dendritic cells | 1,27E-02 | CXCL12 (includes EG:20315) | 1 |
| Chemotaxis of pro-b lymphocytes | 1,27E-02 | CXCL12 (includes EG:20315) | 1 |
| Extravasation of hepatoma cell lines | 1,27E-02 | CXCL12 (includes EG:20315) | 1 |
| Invasion of hybrid cells | 1,27E-02 | SPP1 (includes EG:20750) | 1 |
| Migration of b-lymphocyte derived cell lines | 1,27E-02 | CXCL12 (includes EG:20315) | 1 |
| Migration of haec cells | 1,27E-02 | CXCL12 (includes EG:20315) | 1 |
| Migration of cholangiocarcinoma cell lines | 1,27E-02 | CXCL12 (includes EG:20315) | 1 |
| Migration of rhabdomyosarcoma cell lines | 1,27E-02 | IGF2 | 1 |
| Mobilization of osteoclasts | 1,27E-02 | CXCL12 (includes EG:20315) | 1 |
| Recruitment of inflammatory leukocytes | 1,27E-02 | CXCL12 (includes EG:20315) | 1 |
| Adhesion of memory t lymphocytes | 1,89E-02 | CCL20 | 1 |
| Beat of cilia | 1,89E-02 | ADORA2B | 1 |
| Chemotaxis of naive t lymphocytes | 1,89E-02 | CXCL12 (includes EG:20315) | 1 |
| Migration of langerhans cell precursors | 1,89E-02 | CCL20 | 1 |
| Migration of megakaryocytes | 1,89E-02 | CXCL12 (includes EG:20315) | 1 |
| Mobility of carcinoma cell lines | 1,89E-02 | SCUBE3 | 1 |
| Mobility of lung cancer cell lines | 1,89E-02 | SCUBE3 | 1 |
| Mobilization of colony-forming granulocyte-macrophages | 1,89E-02 | CXCL12 (includes EG:20315) | 1 |
| Mobilization of stem cells | 1,89E-02 | CXCL12 (includes EG:20315) | 1 |
| Chemotaxis of cervical cancer cell lines | 2,52E-02 | CXCL12 (includes EG:20315) | 1 |
| Chemotaxis of melanoma cell lines | 2,52E-02 | CXCL12 (includes EG:20315) | 1 |
| Chemotaxis of peripheral blood lymphocytes | 2,52E-02 | CXCL12 (includes EG:20315) | 1 |
| Chemotaxis of rhabdomyosarcoma cell lines | 2,52E-02 | CXCL12 (includes EG:20315) | 1 |
| Migration of peripheral t lymphocyte | 2,52E-02 | CXCL12 (includes EG:20315) | 1 |
| Migration of plasmacytoid dendritic cells | 2,52E-02 | CXCL12 (includes EG:20315) | 1 |
| Migration of progenitor cells | 2,52E-02 | CXCL12 (includes EG:20315) | 1 |
| Chemotaxis of blood-derived mast cells | 3,14E-02 | CXCL12 (includes EG:20315) | 1 |
| Migration of lymphoblasts | 3,14E-02 | CXCL12 (includes EG:20315) | 1 |
| Migration of b lymphocytes | 3,75E-02 | CXCL12 (includes EG:20315) | 1 |
| Chemotaxis of germ cell tumor cell lines | 4,36E-02 | CXCL12 (includes EG:20315) | 1 |
| Chemotaxis of lymphoma cell lines | 4,36E-02 | CXCL12 (includes EG:20315) | 1 |
| Chemotaxis of monocyte-derived dendritic cells | 4,36E-02 | CXCL12 (includes EG:20315) | 1 |
| Invasion of adenocarcinoma cell lines | 4,36E-02 | SCUBE3 | 1 |
| Invasion of kidney cancer cell lines | 4,36E-02 | SFRP1 | 1 |
| Migration of bone marrow precursor cells | 4,36E-02 | CXCL12 (includes EG:20315) | 1 |
| Migration of thymocytes | 4,36E-02 | CXCL12 (includes EG:20315) | 1 |
| Nk cell migration | 4,97E-02 | CXCL12 (includes EG:20315) | 1 |
| Migration of peripheral blood monocytes | 4,97E-02 | CXCL12 (includes EG:20315) | 1 |
| Migration of skin cancer cell lines | 4,97E-02 | LAMC2 | 1 |
| Migration of trophoblast cells | 4,97E-02 | IGF2 | 1 |
| **Cell signaling and interaction (15 molecules)** | | | |
| Adhesion of bone marrow cells | 2,37E-04 | CCL20,CXCL12 (includes EG:20315) | 2 |
| Activation of osteoclasts | 1,39E-03 | CXCL12 (includes EG:20315),IGF2 | 2 |
| Binding of tumor cell lines | 2,20E-03 | CXADR,CXCL12 (includes EG:20315),IGF2,OXTR,SPP1 (includes EG:20750) | 5 |
| Adhesion of t lymphocytes | 2,21E-03 | CCL20,CXCL12 (includes EG:20315),SPP1 (includes EG:20750) | 3 |
| Activation of trophoblast cells | 6,35E-03 | IGF2 | 1 |
| Adhesion of th17 cells | 6,35E-03 | CCL20 | 1 |
| Adhesion of cell surfasse | 6,35E-03 | NCAM1 | 1 |
| Adhesion of mesenchymal stem cells | 6,35E-03 | CCL20 | 1 |
| Adhesion of small cell lung cancer cells | 6,35E-03 | CXCL12 (includes EG:20315) | 1 |
| Binding of granulosa cells | 6,35E-03 | IGF2 | 1 |
| Binding of rhabdomyosarcoma cell lines | 6,35E-03 | CXCL12 (includes EG:20315) | 1 |
| Long-term potentiation of hippocampus | 6,35E-03 | PLAT | 1 |
| Reassembly of tight junctions | 6,35E-03 | CLDN1 | 1 |
| Retention of cell-associated matrix | 6,35E-03 | SPP1 (includes EG:20750) | 1 |
| Structural integrity of tight junctions | 6,35E-03 | CXADR | 1 |
| Binding of breast cancer cell lines | 7,74E-03 | OXTR,SPP1 (includes EG:20750) | 2 |
| Recruitment of leukocytes | 7,74E-03 | CCL7,CXCL12 (includes EG:20315) | 2 |
| Activation of breast cancer cell lines | 1,27E-02 | MMP1 (includes EG:300339) | 1 |
| Activation of smooth muscle cell lines | 1,27E-02 | TSLP | 1 |
| Adhesion of pro-b lymphocytes | 1,27E-02 | CXCL12 (includes EG:20315) | 1 |
| Binding of th1 cells | 1,27E-02 | CXCL12 (includes EG:20315) | 1 |
| Binding of bladder cancer cell lines | 1,27E-02 | CXADR | 1 |
| Chemoattraction of langerhans cells | 1,27E-02 | CCL20 | 1 |
| Fusion of thymocytes | 1,27E-02 | CXCL12 (includes EG:20315) | 1 |
| Recruitment of inflammatory leukocytes | 1,27E-02 | CXCL12 (includes EG:20315) | 1 |
| Binding of cells | 1,51E-02 | CCL20,CXADR,CXCL12 (includes EG:20315),IGF2,OXTR,SPP1 (includes EG:20750) | 6 |
| Adhesion of bone marrow stromal cells | 1,89E-02 | CXCL12 (includes EG:20315) | 1 |
| Adhesion of memory t lymphocytes | 1,89E-02 | CCL20 | 1 |
| Adhesion of osteoblastos | 1,89E-02 | TNFRSF11B | 1 |
| Recognition of neurons | 1,89E-02 | NTM | 1 |
| Adhesion of connective tissue cells | 2,42E-02 | CXCL12 (includes EG:20315),TNFRSF11B | 2 |
| Activation of micróglia | 2,52E-02 | PLAT | 1 |
| Activation of myeloid dendritic cells | 2,52E-02 | TSLP | 1 |
| Binding of megakaryocytes | 2,52E-02 | CXCL12 (includes EG:20315) | 1 |
| Binding of ovarian cancer cell lines | 2,52E-02 | IGF2 | 1 |
| Release of acetylcholine | 2,52E-02 | IGF2 | 1 |
| Binding of lymphocytes | 3,04E-02 | CCL20,CXCL12 (includes EG:20315) | 2 |
| Adhesion of peripheral blood lymphocytes | 4,97E-02 | CXCL12 (includes EG:20315) | 1 |
| Adhesion of skin cell lines | 4,97E-02 | NTN4 | 1 |
| **Cellular development (21 molecules)** | | | |
| Proliferation of tumor cell lines | 2,47E-02 | ADORA2B,ALDH1A1,CCL20,CXADR,CXCL12 (includes EG:20315),DUSP4,GATA4,IGF2,MEG3,NDN,OXTR,PLAT,SPP1 (includes EG:20750),SULT1E1 | 14 |
| Proliferation of immune cells | 1,82E-02 | CCL20,CXCL12 (includes EG:20315),IGF2,SPP1 (includes EG:20750),TSLP | 5 |
| Proliferation of t lymphocytes | 2,16E-02 | CXCL12 (includes EG:20315),IGF2,SPP1 (includes EG:20750),TSLP | 4 |
| Differentiation of tumor cell lines | 2,95E-02 | CTSK,ENO1,IGF2,NCAM1 | 4 |
| Proliferation of prostate cancer cell lines | 4,01E-02 | ALDH1A1,CXADR,IGF2,OXTR | 4 |
| Proliferation of bladder cancer cell lines | 6,47E-04 | CXADR,IGF2,PLAT | 3 |
| Proliferation of embryonic cell lines | 1,12E-02 | IGF2,OXTR,SCUBE3 | 3 |
| Proliferation of ovarian cancer cell lines | 1,73E-02 | CXCL12 (includes EG:20315),GATA4,IGF2 | 3 |
| Proliferation of fibroblastos | 1,79E-02 | IGF2,SFRP1,TNFRSF11B | 3 |
| Proliferation of hepatoma cell lines | 3,28E-02 | CXCL12 (includes EG:20315),IGF2,SPP1 (includes EG:20750) | 3 |
| Proliferation of osteoblastos | 4,51E-03 | IGF2,SFRP1 | 2 |
| Differentiation of neuroblastoma cell lines | 7,03E-03 | IGF2,NCAM1 | 2 |
| Proliferation of stem cells | 1,35E-02 | CCL20,CXCL12 (includes EG:20315) | 2 |
| Differentiation of adipocytes | 1,45E-02 | SFRP1,SPP1 (includes EG:20750) | 2 |
| Proliferation of hematopoietic progenitor cells | 3,87E-02 | CCL20,CXCL12 (includes EG:20315) | 2 |
| Formation of myoblasts | 6,35E-03 | IGF2 | 1 |
| Proliferation of granule cell precursors | 6,35E-03 | CXCL12 (includes EG:20315) | 1 |
| Sprouting of microvessel | 6,35E-03 | IGF2 | 1 |
| Formation of myotube | 1,27E-02 | IGF2 | 1 |
| Proliferation of oval cells | 1,27E-02 | CXCL12 (includes EG:20315) | 1 |
| Proliferation of neuroblastoma cells | 1,89E-02 | IGF2 | 1 |
| Colony formation of glioma cells | 2,52E-02 | SFRP1 | 1 |
| Epithelial-mesenchymal transition of lung cancer cell lines | 3,14E-02 | SCUBE3 | 1 |
| Proliferation of neural stem cells | 3,14E-02 | CXCL12 (includes EG:20315) | 1 |
| Epithelial-mesenchymal transition of carcinoma cell lines | 3,75E-02 | SCUBE3 | 1 |
| Proliferation of myeloid progenitor cells | 3,75E-02 | CCL20 | 1 |
| Proliferation of nervous tissue cell lines | 4,36E-02 | CXCL12 (includes EG:20315) | 1 |
| Differentiation of adipoblasts | 4,97E-02 | SPP1 (includes EG:20750) | 1 |
| Osteoclastogenesis of pbmcs | 4,97E-02 | TNFRSF11B | 1 |
| Proliferation of multilineage progenitor cells | 4,97E-02 | CCL20 | 1 |
| **Proliferation and cell growth (27 molecules)** | | | |
| Proliferation of cells | 4,55E-03 | ADORA2B,ALDH1A1,CCL20,CXADR,CXCL12 (includes EG:20315),DCBLD2,DUSP4,EIF1AY,ENO1,FABP4,GATA4,HOXC10,IGF2,MEG3,NDN,NTN4,OXTR,PHLDA1,PLAT,SCUBE3,SFRP1,SPP1 (includes EG:20750),SULT1E1,TNFRSF11B,TNFRSF21,TSLP | 26 |
| Proliferation of tumor cell lines | 2,47E-02 | ADORA2B,ALDH1A1,CCL20,CXADR,CXCL12 (includes EG:20315),DUSP4,GATA4,IGF2,MEG3,NDN,OXTR,PLAT,SPP1 (includes EG:20750),SULT1E1 | 14 |
| Colony formation of cells | 3,08E-03 | ALDH1A1,ANKRD1,CXCL12 (includes EG:20315),ENO1,NDN,PHLDA1,SFRP1 | 7 |
| Proliferation of immune cells | 1,82E-02 | CCL20,CXCL12 (includes EG:20315),IGF2,SPP1 (includes EG:20750),TSLP | 5 |
| Proliferation of t lymphocytes | 2,16E-02 | CXCL12 (includes EG:20315),IGF2,SPP1 (includes EG:20750),TSLP | 4 |
| Proliferation of connective tissue cells | 2,38E-02 | CXCL12 (includes EG:20315),IGF2,SFRP1,TNFRSF11B | 4 |
| Proliferation of prostate cancer cell lines | 4,01E-02 | ALDH1A1,CXADR,IGF2,OXTR | 4 |
| Colony formation of tumor cell lines | 4,78E-02 | ALDH1A1,ANKRD1,ENO1,NDN | 4 |
| Proliferation of embryonic cell lines | 1,12E-02 | IGF2,OXTR,SCUBE3 | 3 |
| Proliferation of ovarian cancer cell lines | 1,73E-02 | CXCL12 (includes EG:20315),GATA4,IGF2 | 3 |
| Proliferation of fibroblastos | 1,79E-02 | IGF2,SFRP1,TNFRSF11B | 3 |
| Proliferation of bladder cancer cell lines | 6,47E-04 | CXADR,IGF2,PLAT | 3 |
| Proliferation of hepatoma cell lines | 3,28E-02 | CXCL12 (includes EG:20315),IGF2,SPP1 (includes EG:20750) | 3 |
| Colony formation of leukemia cell lines | 3,97E-03 | ALDH1A1,ENO1 | 2 |
| Proliferation of osteoblastos | 4,51E-03 | IGF2,SFRP1 | 2 |
| Proliferation of stem cells | 1,35E-02 | CCL20,CXCL12 (includes EG:20315) | 2 |
| Proliferation of hematopoietic progenitor cells | 3,87E-02 | CCL20,CXCL12 (includes EG:20315) | 2 |
| Formation of myoblasts | 6,35E-03 | IGF2 | 1 |
| Proliferation of granule cell precursors | 6,35E-03 | CXCL12 (includes EG:20315) | 1 |
| Formation of myotube | 1,27E-02 | IGF2 | 1 |
| Proliferation of oval cells | 1,27E-02 | CXCL12 (includes EG:20315) | 1 |
| Proliferation of stromal cell lines | 1,27E-02 | IGF2 | 1 |
| Cloning of cells | 1,89E-02 | PHLDA1 | 1 |
| Proliferation of neuroblastoma cells | 1,89E-02 | IGF2 | 1 |
| Colony formation of glioma cells | 2,52E-02 | SFRP1 | 1 |
| Proliferation of neural stem cells | 3,14E-02 | CXCL12 (includes EG:20315) | 1 |
| Proliferation of myeloid progenitor cells | 3,75E-02 | CCL20 | 1 |
| Proliferation of nervous tissue cell lines | 4,36E-02 | CXCL12 (includes EG:20315) | 1 |
| Colony formation of hepatoma cell lines | 4,97E-02 | ANKRD1 | 1 |
| Proliferation of multilineage progenitor cells | 4,97E-02 | CCL20 | 1 |
| **Presentation of antigen (6 molecules)** | | | |
| Chemotaxis of phagocytes | 2,56E-03 | CCL20,CCL7,CXADR,CXCL12 (includes EG:20315),SPP1 (includes EG:20750) | 5 |
| Chemotaxis of neutrophils | 1,01E-03 | CCL7,CXADR,CXCL12 (includes EG:20315),SPP1 (includes EG:20750) | 4 |
| Chemotaxis of dendritic cells | 1,40E-03 | CCL20,CCL7,CXCL12 (includes EG:20315) | 3 |
| Migration of dendritic cells | 1,40E-03 | CCL20,CCL7,CXCL12 (includes EG:20315) | 3 |
| Transmigration of dendritic cells | 1,09E-03 | CCL7,CXCL12 (includes EG:20315) | 2 |
| Chemotaxis of b lymphocytes | 2,52E-03 | CCL20,CXCL12 (includes EG:20315) | 2 |
| Chemoattraction of langerhans cells | 1,27E-02 | CCL20 | 1 |
| Chemotaxis of plasmacytoid dendritic cells | 1,27E-02 | CXCL12 (includes EG:20315) | 1 |
| Chemotaxis of pro-b lymphocytes | 1,27E-02 | CXCL12 (includes EG:20315) | 1 |
| Migration of langerhans cell precursors | 1,89E-02 | CCL20 | 1 |
| Activation of myeloid dendritic cells | 2,52E-02 | TSLP | 1 |
| Migration of plasmacytoid dendritic cells | 2,52E-02 | CXCL12 (includes EG:20315) | 1 |
| Chemotaxis of blood-derived mast cells | 3,14E-02 | CXCL12 (includes EG:20315) | 1 |
| Chemotaxis of monocyte-derived dendritic cells | 4,36E-02 | CXCL12 (includes EG:20315) | 1 |

Supplemental File 16. Table with functional annotation of the five most gene enriched categories with lower p-value of DEG in senesncent hMSC/inv compared to senesncent hMSC/n.

| **Anotação funcional** | **P value** | **Molecules** | **Number of DEG** |
| --- | --- | --- | --- |
| **Cell cycle (55 molecules)** | | |  |
| Cell cycle progression | 1,54E-05 | BHLHE40,BIRC5,BUB1 (includes EG:100307076),CCNA2,CCNB1,CDC20 (includes EG:107995),CDK1,CDKN2C,CDKN3,CENPA,CENPF,CHRM3,DLG1,DLGAP5,DTL,EDN1,EGF (includes EG:13645),FLT1,FOXM1,HBEGF,HGF,IGFBP5,IL11,KIF2C,KIF4A,NDC80,NEK2,NUSAP1,PDCD1LG2 (includes EG:309304),RUNX1,RUNX1T1,SPC25 (includes EG:100144563),TGFB2,TM4SF1,TNC (includes EG:116640),TOP2A,TTK | 37 |
| Mitosis | 1,59E-08 | BIRC5,BUB1 (includes EG:100307076),CCNB1,CDC20 (includes EG:107995),CDK1,CENPA,CENPF,DLG1,DLGAP5,EDN1,EGF (includes EG:13645),FLT1,FOXM1,HBEGF,HGF,IGFBP5,IL11,KIF2C,KIF4A,NDC80,NEK2,NUSAP1,SPC25 (includes EG:100144563),TNC (includes EG:116640),TOP2A,TTK | 26 |
| Interphase | 7,98E-03 | BCAT1,BHLHE40,BIRC5,CCNA2,CCNB1,CCND2,CDK1,CDKN2C,CDKN3,CENPF,CREG1,CYP1B1,DTL,EGF (includes EG:13645),FOXM1,HBEGF,HGF,IGFBP5,KLF4,PBX1,RUNX2,TPD52L1 | 22 |
| M phase | 8,05E-07 | BIRC5,CCDC99,CCNB1,CDC20 (includes EG:107995),CDK1,CENPF,CEP55,DLGAP5,KIF14,KIF20A,KIF4A,NEDD4L,NUSAP1,PRC1 (includes EG:233406),TM4SF1,TOP2A | 16 |
| Segregation of chromosomes | 7,23E-09 | BUB1 (includes EG:100307076),CCNA2,CCNB1,CCNB2,CENPF,CENPW,KIF2C,NCAPG,NDC80,NEK2,NUSAP1,SPC25 (includes EG:100144563),TOP2A | 13 |
| Cytokinesis | 6,87E-06 | BIRC5,CCNB1,CDC20 (includes EG:107995),CEP55,KIF14,KIF20A,KIF4A,NEDD4L,NUSAP1,PRC1 (includes EG:233406),TM4SF1,TOP2A | 12 |
| G2 phase | 3,86E-03 | BIRC5,CCNA2,CCNB1,CDK1,CENPF,DTL,EGF (includes EG:13645),FOXM1,IGFBP5,RUNX2,TPD52L1 | 11 |
| G2/m phase | 1,68E-03 | BIRC5,CCNA2,CCNB1,CDK1,DTL,EGF (includes EG:13645),FOXM1,IGFBP5,RUNX2,TPD52L1 | 10 |
| Mitosis of tumor cell lines | 2,19E-04 | BIRC5,CCNB1,CDC20 (includes EG:107995),CDK1,DLGAP5,FOXM1,SPC25 (includes EG:100144563),TOP2A,TTK | 9 |
| G1/s phase | 4,60E-03 | BCAT1,BIRC5,CDKN2C,CDKN3,CREG1,EGF (includes EG:13645),FOXM1,HGF,KLF4 | 9 |
| Mitogenesis | 4,78E-04 | EDN1,EGF (includes EG:13645),FLT1,HBEGF,HGF,IGFBP5,IL11,TNC (includes EG:116640) | 8 |
| Mitosis of cervical cancer cell lines | 3,07E-04 | BIRC5,CCNB1,CDC20 (includes EG:107995),DLGAP5,SPC25 (includes EG:100144563),TOP2A,TTK | 7 |
| M phase of cervical cancer cell lines | 4,43E-04 | CCDC99,CEP55,KIF14,KIF20A,KIF4A,NEDD4L,TOP2A | 7 |
| G2/m phase transition | 9,49E-04 | BIRC5,CCNA2,CCNB1,CDK1,EGF (includes EG:13645),FOXM1,TPD52L1 | 7 |
| S phase of tumor cell lines | 8,89E-03 | BHLHE40,CCNA2,CCND2,CDK1,EGF (includes EG:13645),FOXM1,PBX1 | 7 |
| Delay in mitosis | 2,02E-06 | CCNB1,CDC20 (includes EG:107995),CDK1,DLGAP5,FOXM1,TOP2A | 6 |
| Cytokinesis of tumor cell lines | 1,08E-03 | CEP55,KIF14,KIF20A,KIF4A,NEDD4L,TOP2A | 6 |
| Entry into interphase | 1,08E-02 | BHLHE40,EGF (includes EG:13645),FOXM1,HBEGF,PBX1,RUNX2 | 6 |
| Arrest in g2 phase of tumor cell lines | 1,97E-02 | CCNA2,CCNB1,CDK1,EGF (includes EG:13645),FOXM1,IGFBP5 | 6 |
| Delay in mitosis of tumor cell lines | 2,00E-05 | CCNB1,CDC20 (includes EG:107995),CDK1,DLGAP5,TOP2A | 5 |
| Cytokinesis of cervical cancer cell lines | 2,07E-03 | CEP55,KIF14,KIF20A,KIF4A,NEDD4L | 5 |
| Ploidy | 2,18E-02 | BIRC5,EDN1,NEK2,SCIN,TOP2A | 5 |
| Mitogenesis of connective tissue cells | 8,68E-05 | EDN1,EGF (includes EG:13645),HBEGF,IL11 | 4 |
| Delay in mitosis of cervical cancer cell lines | 1,34E-04 | CCNB1,CDC20 (includes EG:107995),DLGAP5,TOP2A | 4 |
| Spindle checkpoint of cells | 1,07E-03 | BIRC5,BUB1 (includes EG:100307076),DLGAP5,TTK | 4 |
| Arrest in g2/m phase transition of tumor cell lines | 5,10E-03 | CCNA2,CCNB1,CDK1,EGF (includes EG:13645) | 4 |
| Formation of mitotic spindle | 1,71E-02 | BIRC5,KIF2C,KIF4A,NEK2 | 4 |
| Mitogenesis of fibroblasts | 5,82E-04 | EGF (includes EG:13645),HBEGF,IL11 | 3 |
| Mitogenesis of smooth muscle cells | 9,13E-04 | EDN1,EGF (includes EG:13645),HBEGF | 3 |
| Polyploidization of cells | 1,88E-03 | BIRC5,SCIN,TOP2A | 3 |
| Segregation of sister chromatids | 3,32E-03 | CCNA2,NDC80,NUSAP1 | 3 |
| Delay in initiation of m phase | 6,47E-03 | BIRC5,CCNB1,TOP2A | 3 |
| Checkpoint control of mitotic spindle | 6,89E-04 | CDC20 (includes EG:107995),NDC80 | 2 |
| Mitogenesis of pancreatic cancer cell lines | 2,03E-03 | EGF (includes EG:13645),HGF | 2 |
| Mitogenesis of cervical cancer cell lines | 3,99E-03 | EGF (includes EG:13645),IGFBP5 | 2 |
| Senescence of vascular endothelial cells | 6,53E-03 | FLT1,TM4SF1 | 2 |
| Mitotic exit | 9,63E-03 | BIRC5,CDC20 (includes EG:107995) | 2 |
| Polyploidization of tumor cell lines | 9,63E-03 | SCIN,TOP2A | 2 |
| Mitosis of bone cancer cell lines | 1,32E-02 | CDK1,FOXM1 | 2 |
| Arrest in g2 phase of endothelial cell lines | 2,63E-02 | FOXM1 | 1 |
| Arrest in s phase of ovarian cancer cell lines | 2,63E-02 | CDK1 | 1 |
| Arrest in spindle checkpoint of cervical cancer cell lines | 2,63E-02 | DLGAP5 | 1 |
| Arrest in sub-g1 phase of endometrial cancer cell lines | 2,63E-02 | CYP1B1 | 1 |
| Cell division of gonadal cell lines | 2,63E-02 | CHRM3 | 1 |
| Cytokinesis of fibrosarcoma cell lines | 2,63E-02 | TOP2A | 1 |
| Delay in g1/s phase transition of germ cell tumor cell lines | 2,63E-02 | CREG1 | 1 |
| Delay in initiation of mitotic exit of cervical cancer cell lines | 2,63E-02 | CDC20 (includes EG:107995) | 1 |
| Delay in segregation of sister chromatids | 2,63E-02 | CCNA2 | 1 |
| Endoreduplication of cervical cancer cell lines | 2,63E-02 | KIF14 | 1 |
| Entry into g2/m phase of endothelial cells | 2,63E-02 | RUNX2 | 1 |
| Entry into cell cycle progression of gonadal cell lines | 2,63E-02 | CHRM3 | 1 |
| Exit from g2/m phase of endothelial cells | 2,63E-02 | RUNX2 | 1 |
| Exit from m phase | 2,63E-02 | CDC20 (includes EG:107995) | 1 |
| Hypodiploidy of prostate cancer cell lines | 2,63E-02 | BIRC5 | 1 |
| Initiation of mitosis | 2,63E-02 | CDK1 | 1 |
| **Cellular organization and association (47 molecules)** | | |  |
| Segregation of chromosomes | 7,23E-09 | BUB1 (includes EG:100307076),CCNA2,CCNB1,CCNB2,CENPF,CENPW,KIF2C,NCAPG,NDC80,NEK2,NUSAP1,SPC25 (includes EG:100144563),TOP2A | 13 |
| Formation of filaments | 1,49E-02 | CHRM3,DLGAP5,EGF (includes EG:13645),FOXM1,GNG2,HGF,KISS1,MME,STMN2,TGFBI | 10 |
| Alignment of chromosomes | 3,12E-07 | BIRC5,CCNA2,DLGAP5,KIF14,KIF2C,NCAPG,TTK | 7 |
| Growth of neurites | 4,04E-03 | EFNB2,EGF (includes EG:13645),HGF,L1CAM,MMP2,TNC (includes EG:116640) | 6 |
| Chromosomal congression of chromosomes | 3,02E-05 | KIF14,KIF2C,NDC80,SGOL2 | 4 |
| Formation of mitotic spindle | 1,71E-02 | BIRC5,KIF2C,KIF4A,NEK2 | 4 |
| Formation of lamellipodia | 2,39E-02 | EGF (includes EG:13645),GRP,HGF,SPATA13 | 4 |
| Organization of nucleus | 2,59E-02 | KIF4A,NDC80,SPC25 (includes EG:100144563),TTK | 4 |
| Segregation of sister chromatids | 3,32E-03 | CCNA2,NDC80,NUSAP1 | 3 |
| Formation of nucleus | 7,81E-03 | CDK1,EGF (includes EG:13645),LMNB1 | 3 |
| Polymerization of microtubules | 1,28E-02 | DLGAP5,FOXM1,STMN2 | 3 |
| Organization of mitotic spindle | 2,16E-02 | NDC80,SPC25 (includes EG:100144563),TTK | 3 |
| Depolymerization of filaments | 2,42E-02 | F2RL1,KIF2C,STMN2 | 3 |
| Function of tight junctions | 6,89E-04 | DLG1,MPP7 | 2 |
| Structural integrity of plasma membrane | 6,89E-04 | CXADR,DLG1 | 2 |
| Formation of nuclear envelope | 2,03E-03 | CDK1,EGF (includes EG:13645) | 2 |
| Alignment of sister chromatids | 3,99E-03 | NDC80,TOP2A | 2 |
| Elongation of mitotic spindle | 3,99E-03 | PRC1 (includes EG:233406),SPC25 (includes EG:100144563) | 2 |
| Attachment of spindle fibers | 1,74E-02 | CASC5,NDC80 | 2 |
| Disruption of microtubules | 1,74E-02 | EGF (includes EG:13645),STMN2 | 2 |
| Formation of tight junctions | 1,74E-02 | DLG1,MPP7 | 2 |
| Destabilization of microtubules | 2,19E-02 | KIF2C,STMN2 | 2 |
| Missegregation of chromosomes | 2,19E-02 | CENPA,KIF4A | 2 |
| Association of kinetochores | 2,63E-02 | BIRC5 | 1 |
| Binding of myosin filaments | 2,63E-02 | FHL1 (includes EG:14199) | 1 |
| Communication of gap junctions | 2,63E-02 | MCAM | 1 |
| Delay in alignment of chromosomes | 2,63E-02 | CCNA2 | 1 |
| Delay in segregation of sister chromatids | 2,63E-02 | CCNA2 | 1 |
| Dispersal of centrosome | 2,63E-02 | NEK2 | 1 |
| Formation of clathrin-coated pits | 2,63E-02 | EGF (includes EG:13645) | 1 |
| Formation of gap junction plaques | 2,63E-02 | EDN1 | 1 |
| Formation of midzone | 2,63E-02 | KIF4A | 1 |
| Formation of sarcomere | 2,63E-02 | HBEGF | 1 |
| **Proliferation and cell growth (104 molecules)** | | |  |
| Proliferation of cells | 2,42E-06 | AK4,AKR1C3,ANGPTL1,AQP1,BCAT1,BDKRB2,BHLHE40,BIRC5,BUB1 (includes EG:100307076),CASP1,CCNA2,CCND2,CD274,CDCP1,CDK1,CDKN2C,CDKN3,CDT1,CHRM3,COL4A2 (includes EG:12827),COL6A3,CREG1,CXADR,CYP1B1,DAB2,DIRAS3,DLG1,DLGAP5,DSP,DTL,EDN1,EFNB2,EGF (includes EG:13645),EGR1,EGR2,ENPP1,ETV1,F2RL1,FGF1,FGF7,FHL1 (includes EG:14199),FLT1,FOXM1,FST,GAS6,GPNMB,GRP,HBEGF,HGF,HMMR,HTR2B,IFIT3,IFITM1,IGFBP5,IL11,INSIG1,JAG1,KIAA0101,KIF20A,KIF2C,KISS1,KLF4,LAMA2,LAMA5,MCAM,MMP2,NCAPG,NEK2,NOG,NRP1 (includes EG:18186),NUPR1,PBK,PDCD1LG2 (includes EG:309304),PDGFRA,PMP22,PTGER2,PTGS1,PTPRG,QPCT,RGS4,RGS5,RRM2,RUNX1,RUNX1T1,RUNX2,SCIN,TGFB2,TGFBI,TNFRSF21,TOP2A,TPD52,TTK,TYRP1,UBE2L6,UNC5B,USP18,VAMP8,VDR,WISP1,WNT16 | 100 |
| Proliferation of tumor cell lines | 4,46E-05 | AKR1C3,BDKRB2,BIRC5,BUB1 (includes EG:100307076),CCNA2,CCND2,CDCP1,CDK1,CDT1,CHRM3,COL4A2 (includes EG:12827),COL6A3,CREG1,CXADR,CYP1B1,DAB2,DIRAS3,DTL,EDN1,EGF (includes EG:13645),EGR1,EGR2,ETV1,F2RL1,FGF1,FGF7,FLT1,FOXM1,FST,GAS6,GRP,HBEGF,HGF,HMMR,IGFBP5,IL11,JAG1,KIAA0101,KIF20A,KISS1,KLF4,NCAPG,NEK2,NRP1 (includes EG:18186),PBK,PDGFRA,PTGER2,PTPRG,RRM2,RUNX1,RUNX1T1,RUNX2,TPD52,USP18,VDR,WISP1 | 56 |
| Proliferation of breast cancer cell lines | 1,39E-03 | BDKRB2,BIRC5,BUB1 (includes EG:100307076),CCND2,CDK1,COL6A3,DAB2,EGF (includes EG:13645),FGF1,FGF7,FOXM1,HBEGF,HGF,IGFBP5,KISS1,NRP1 (includes EG:18186),PBK,PTPRG,VDR | 19 |
| Colony formation of cells | 1,10E-03 | BIRC5,CADM1,DIRAS3,EDN1,EGF (includes EG:13645),HGF,IFIH1,IL11,KLF4,LOXL4,NUPR1,PBK,RRM2,RUNX1,RUNX1T1,RUNX2,TGFB2,TPD52 | 18 |
| Proliferation of endothelial cells | 2,56E-05 | ANGPTL1,COL4A2 (includes EG:12827),DAB2,DLG1,EDN1,EFNB2,EGF (includes EG:13645),F2RL1,FGF1,FLT1,HGF,HTR2B,NRP1 (includes EG:18186),RGS5,RUNX2 | 15 |
| Proliferation of prostate cancer cell lines | 3,06E-04 | BIRC5,CXADR,DAB2,DIRAS3,EGF (includes EG:13645),FGF1,FGF7,FST,GRP,HBEGF,HGF,IGFBP5,PDGFRA,TPD52,VDR | 15 |
| Proliferation of connective tissue cells | 1,79E-04 | AQP1,EDN1,EGF (includes EG:13645),FGF1,FGF7,GAS6,HGF,IGFBP5,NOG,PDGFRA,TGFB2,VDR,WISP1,WNT16 | 14 |
| Proliferation of carcinoma cell lines | 8,95E-03 | BIRC5,CDCP1,DAB2,EGF (includes EG:13645),FGF7,FOXM1,GAS6,GRP,HGF,KISS1,NEK2,PDGFRA | 12 |
| Colony formation of tumor cell lines | 1,14E-02 | CADM1,DIRAS3,EDN1,EGF (includes EG:13645),HGF,KLF4,LOXL4,NUPR1,PBK,RRM2,RUNX2,TPD52 | 12 |
| Proliferation of colon cancer cell lines | 1,51E-02 | BIRC5,CDK1,CDT1,EDN1,EGF (includes EG:13645),EGR2,F2RL1,FST,GRP,KLF4,RRM2 | 11 |
| Proliferation of epithelial cells | 1,16E-03 | CCND2,EGF (includes EG:13645),FGF7,HBEGF,HGF,IL11,RGS4,TGFB2,VDR,WNT16 | 10 |
| Proliferation of tumor cells | 1,05E-02 | CASP1,EDN1,EGF (includes EG:13645),EGR1,FOXM1,FST,HGF,JAG1,MCAM,TGFB2 | 10 |
| Proliferation of kidney cell lines | 6,33E-04 | BIRC5,DIRAS3,DLGAP5,EGF (includes EG:13645),FGF1,HGF,PMP22,QPCT,UNC5B | 9 |
| Proliferation of epithelial cell lines | 2,28E-03 | BIRC5,DAB2,DIRAS3,DLGAP5,EGR1,FGF1,HGF,QPCT,UNC5B | 9 |
| Proliferation of vascular endothelial cells | 3,53E-04 | DAB2,EFNB2,EGF (includes EG:13645),F2RL1,FLT1,HGF,NRP1 (includes EG:18186),RGS5 | 8 |
| Proliferation of hepatoma cell lines | 8,42E-03 | BIRC5,EGF (includes EG:13645),F2RL1,FGF7,GRP,HGF,NCAPG,RRM2 | 8 |
| Proliferation of pancreatic cancer cell lines | 1,53E-03 | EGF (includes EG:13645),FOXM1,HBEGF,HGF,KIAA0101,KIF20A,RRM2 | 7 |
| Proliferation of embryonic cell lines | 3,00E-03 | BIRC5,DIRAS3,DLGAP5,FGF1,HGF,QPCT,UNC5B | 7 |
| Proliferation of ovarian cancer cell lines | 7,41E-03 | DAB2,DIRAS3,EDN1,EGF (includes EG:13645),FST,HBEGF,PDGFRA | 7 |
| Arrest in growth of cells | 2,26E-02 | DAB2,EGF (includes EG:13645),EGR1,FGF1,KLF4,NRP1 (includes EG:18186),RUNX1 | 7 |
| Proliferation of brain cancer cell lines | 2,48E-02 | BIRC5,DTL,EGF (includes EG:13645),EGR1,HGF,JAG1,PDGFRA | 7 |
| Proliferation of keratinocytes | 1,22E-03 | EGF (includes EG:13645),FGF7,HGF,TGFB2,VDR,WNT16 | 6 |
| Proliferation of muscle cells | 2,43E-02 | EDN1,FHL1 (includes EG:14199),HBEGF,HGF,IGFBP5,NOG | 6 |
| Proliferation of lymphatic system cells | 7,55E-03 | BIRC5,EDN1,FGF1,HGF,TGFB2 | 5 |
| Colony formation of breast cancer cell lines | 1,34E-02 | DIRAS3,EGF (includes EG:13645),NUPR1,PBK,RUNX2 | 5 |
| Proliferation of lymphatic endothelial cells | 1,34E-03 | EDN1,FGF1,HGF | 3 |
| Proliferation of neuronal cells | 1,09E-02 | EGF (includes EG:13645),HGF,JAG1 | 3 |
| Proliferation of skin cancer cell lines | 2,16E-02 | EGF (includes EG:13645),HGF,PTGER2 | 3 |
| Proliferation of embryonic cells | 2,42E-02 | EGF (includes EG:13645),FGF7,JAG1 | 3 |
| Expansion of erythroid cells | 6,89E-04 | RUNX1,RUNX1T1 | 2 |
| Colony formation of glioma cells | 3,99E-03 | HGF,TGFB2 | 2 |
| Formation of foam cells | 6,53E-03 | EGF (includes EG:13645),IL33 | 2 |
| Proliferation of heart cells | 6,53E-03 | NOG,WISP1 | 2 |
| Proliferation of embryonic cancer cell lines | 9,63E-03 | DAB2,EGF (includes EG:13645) | 2 |
| Proliferation of exocrine cells | 9,63E-03 | FGF1,FGF7 | 2 |
| Colony formation of erythroid cells | 1,32E-02 | RUNX1,RUNX1T1 | 2 |
| Colony formation of melanoma cell lines | 1,32E-02 | EDN1,HGF | 2 |
| Proliferation of mesenchymal cells | 1,32E-02 | EGF (includes EG:13645),FGF7 | 2 |
| Proliferation of neurosphere cells | 1,32E-02 | EGF (includes EG:13645),JAG1 | 2 |
| Proliferation of leiomyoma cells | 2,19E-02 | EDN1,EGF (includes EG:13645) | 2 |
| Arrest in growth of chondrocyte cell lines | 2,63E-02 | FGF1 | 1 |
| Arrest in growth of nervous tissue cell lines | 2,63E-02 | NRP1 (includes EG:18186) | 1 |
| Colony formation of sarcoma cell lines | 2,63E-02 | HGF | 1 |
| Expansion of dopaminergic neurons | 2,63E-02 | EGF (includes EG:13645) | 1 |
| Expansion of mesencephalic neurons | 2,63E-02 | EGF (includes EG:13645) | 1 |
| Formation of breast cell lines | 2,63E-02 | RUNX2 | 1 |
| Formation of megakaryoblasts | 2,63E-02 | IL11 | 1 |
| Growth of luminal epithelial cells | 2,63E-02 | HGF | 1 |
| Induction of hepatoma cell lines | 2,63E-02 | HGF | 1 |
| Inhibition of skin cancer cell lines | 2,63E-02 | EGF (includes EG:13645) | 1 |
| **Cell movement (70 molecules)** | | |  |
| Cell movement | 7,53E-05 | AGTR1,ANGPTL1,CCL8,CDCP1,CDK1,CHI3L1,CHRM3,COL4A2 (includes EG:12827),CXADR,DAB2,EDN1,EFNB2,EGF (includes EG:13645),EGR1,F2RL1,FBLN5,FGF1,FGF7,FHL1 (includes EG:14199),FLT1,FOXM1,FPR1,GATA3,GRP,HBEGF,HGF,HMMR,HOXD10,IGFBP5,IL11,IL33,KISS1,KLF4,L1CAM,MMP2,MYO10,NPPB,NREP,NRP1 (includes EG:18186),OLR1,PARP9,PBK,PDGFRA,PMP22,RGS4,RUNX2,SPATA13,TGFB2,TNC (includes EG:116640),WISP1 | 50 |
| Migration of cells | 4,12E-05 | ANGPTL1,CCL8,CDK1,CHI3L1,CHRM3,COL4A2 (includes EG:12827),CXADR,DAB2,EDN1,EFNB2,EGF (includes EG:13645),EGR1,F2RL1,FBLN5,FGF1,FGF7,FHL1 (includes EG:14199),FLT1,FOXM1,FPR1,GATA3,GRP,HBEGF,HGF,HMMR,HOXD10,IGFBP5,IL11,IL33,KISS1,KLF4,L1CAM,MMP2,MYO10,NPPB,NREP,NRP1 (includes EG:18186),OLR1,PARP9,PDGFRA,PMP22,RGS4,RUNX2,SPATA13,TGFB2,TNC (includes EG:116640) | 46 |
| Cell movement of tumor cell lines | 1,73E-02 | CDCP1,DAB2,EDN1,EGF (includes EG:13645),EGR1,F2RL1,FBLN5,FGF1,FGF7,FLT1,FOXM1,GRP,HGF,HMMR,KISS1,KLF4,L1CAM,MMP2,MYO10,NREP,NRP1 (includes EG:18186),PBK,RUNX2,TNC (includes EG:116640),WISP1 | 25 |
| Invasion of cells | 2,91E-03 | CHI3L1,CYP1B1,DAB2,EDN1,EGF (includes EG:13645),ETV1,FBLN5,FLT1,FOXM1,FST,GRP,HBEGF,HGF,HMMR,KISS1,KLF4,MCAM,MMP2,NRP1 (includes EG:18186),PTGER2,RGS4,RRM2,UNC5B,WISP1 | 24 |
| Migration of tumor cell lines | 1,29E-02 | DAB2,EDN1,EGF (includes EG:13645),EGR1,F2RL1,FBLN5,FGF1,FGF7,FOXM1,GRP,HGF,HMMR,KISS1,KLF4,L1CAM,MMP2,MYO10,NREP,NRP1 (includes EG:18186),RUNX2,TNC (includes EG:116640) | 21 |
| Invasion of tumor cell lines | 1,63E-02 | CYP1B1,DAB2,EDN1,EGF (includes EG:13645),ETV1,FBLN5,FOXM1,GRP,HBEGF,HGF,HMMR,KISS1,KLF4,MMP2,NRP1 (includes EG:18186),PTGER2,RRM2,UNC5B,WISP1 | 19 |
| Chemotaxis | 5,22E-03 | AGTR1,CCL8,CXADR,EFNB2,EGF (includes EG:13645),F2RL1,FLT1,FPR1,HBEGF,HGF,IL33,KISS1,L1CAM,MMP2,NRP1 (includes EG:18186),PDGFRA,TGFB2 | 17 |
| Homing of cells | 7,95E-03 | AGTR1,CCL8,CXADR,EGF (includes EG:13645),F2RL1,FLT1,FPR1,HBEGF,HGF,IL33,KISS1,L1CAM,MMP2,NRP1 (includes EG:18186),PDGFRA,TGFB2 | 16 |
| Chemotaxis of cells | 1,46E-02 | AGTR1,CCL8,CXADR,EGF (includes EG:13645),F2RL1,FLT1,FPR1,HBEGF,HGF,IL33,KISS1,MMP2,NRP1 (includes EG:18186),PDGFRA,TGFB2 | 15 |
| Cytokinesis | 6,87E-06 | BIRC5,CCNB1,CDC20 (includes EG:107995),CEP55,KIF14,KIF20A,KIF4A,NEDD4L,NUSAP1,PRC1 (includes EG:233406),TM4SF1,TOP2A | 12 |
| Migration of endothelial cells | 1,87E-03 | ANGPTL1,COL4A2 (includes EG:12827),EDN1,EFNB2,EGF (includes EG:13645),F2RL1,FGF1,FLT1,GATA3,HGF,NRP1 (includes EG:18186),OLR1 | 12 |
| Cell movement of tumor cells | 1,84E-05 | CDK1,EGF (includes EG:13645),FLT1,HGF,HMMR,L1CAM,MMP2,PARP9,TGFB2 | 9 |
| Cell movement of cancer cells | 3,15E-05 | CDK1,FLT1,HGF,HMMR,L1CAM,MMP2,PARP9,TGFB2 | 8 |
| Cell movement of colon cancer cell lines | 3,49E-03 | CDCP1,EGF (includes EG:13645),EGR1,F2RL1,GRP,HGF,KLF4 | 7 |
| Cell movement of epithelial cells | 1,08E-03 | CHRM3,FGF7,HBEGF,HGF,PMP22,RGS4 | 6 |
| Cytokinesis of tumor cell lines | 1,08E-03 | CEP55,KIF14,KIF20A,KIF4A,NEDD4L,TOP2A | 6 |
| Migration of colon cancer cell lines | 3,38E-03 | EGF (includes EG:13645),EGR1,F2RL1,GRP,HGF,KLF4 | 6 |
| Cell movement of carcinoma cell lines | 9,41E-03 | EGF (includes EG:13645),GRP,HGF,KISS1,RUNX2,WISP1 | 6 |
| Invasion of pancreatic cancer cell lines | 7,27E-04 | EGF (includes EG:13645),FOXM1,HGF,NRP1 (includes EG:18186),RRM2 | 5 |
| Migration of epithelial cells | 1,18E-03 | CHRM3,FGF7,HBEGF,PMP22,RGS4 | 5 |
| Cytokinesis of cervical cancer cell lines | 2,07E-03 | CEP55,KIF14,KIF20A,KIF4A,NEDD4L | 5 |
| Invasion of colon cancer cell lines | 1,06E-02 | GRP,HGF,KLF4,NRP1 (includes EG:18186),UNC5B | 5 |
| Cell movement of smooth muscle cells | 1,24E-02 | FHL1 (includes EG:14199),HBEGF,HGF,IGFBP5,MMP2 | 5 |
| Migration of carcinoma cell lines | 1,67E-02 | EGF (includes EG:13645),GRP,HGF,KISS1,RUNX2 | 5 |
| Migration of neurons | 3,81E-04 | EGF (includes EG:13645),HBEGF,HGF,L1CAM | 4 |
| Invasion of squamous cell carcinoma cell lines | 8,42E-03 | EGF (includes EG:13645),GRP,HBEGF,HGF | 4 |
| Migration of neuroglia | 5,82E-04 | CHI3L1,HGF,TGFB2 | 3 |
| Migration of keratinocyte cancer cell lines | 6,47E-03 | EGF (includes EG:13645),FGF7,HGF | 3 |
| Migration of fibroblast cell lines | 7,81E-03 | EGF (includes EG:13645),HBEGF,PDGFRA | 3 |
| Scattering of tumor cell lines | 7,81E-03 | EGF (includes EG:13645),HGF,L1CAM | 3 |
| Migration of lymphatic system cells | 1,28E-02 | FGF1,HGF,PDGFRA | 3 |
| Migration of pancreatic cancer cell lines | 1,28E-02 | EGF (includes EG:13645),FOXM1,HGF | 3 |
| Migration of keratinocytes | 1,47E-02 | CHRM3,FGF7,HBEGF | 3 |
| Invasion of fibrosarcoma cell lines | 1,68E-02 | FBLN5,KISS1,MMP2 | 3 |
| Migration of ovarian cancer cell lines | 1,68E-02 | EDN1,EGF (includes EG:13645),HGF | 3 |
| Migration of skin cell lines | 1,91E-02 | EGF (includes EG:13645),HGF,PDGFRA | 3 |
| Homing of embryonic cell lines | 2,16E-02 | AGTR1,EGF (includes EG:13645),L1CAM | 3 |
| Homing of epithelial cell lines | 2,16E-02 | AGTR1,EGF (includes EG:13645),L1CAM | 3 |
| Scattering of stomach cancer cell lines | 6,89E-04 | EGF (includes EG:13645),HGF | 2 |
| Chemorepulsion of brain cancer cell lines | 2,03E-03 | FLT1,NRP1 (includes EG:18186) | 2 |
| Migration of astrocytes | 2,03E-03 | CHI3L1,HGF | 2 |
| Migration of sarcoma cell lines | 2,03E-03 | EGR1,HGF | 2 |
| Chemotaxis of fibroblast cell lines | 3,99E-03 | EGF (includes EG:13645),PDGFRA | 2 |
| Migration of extravillous trophoblast cells | 6,53E-03 | CCL8,IL11 | 2 |
| Scattering of breast cancer cell lines | 9,63E-03 | HGF,L1CAM | 2 |
| Chemotaxis of gonadal cell lines | 1,32E-02 | FPR1,KISS1 | 2 |
| Invasion of trophoblast cells | 1,32E-02 | EGF (includes EG:13645),FST | 2 |
| Migration of lymphatic endothelial cells | 1,32E-02 | FGF1,HGF | 2 |
| Cell movement of leukemia cells | 1,74E-02 | FLT1,HMMR | 2 |
| Migration of glioma cells | 1,74E-02 | HGF,TGFB2 | 2 |
| Migration of thyroid tumor cell lines | 1,74E-02 | HGF,KISS1 | 2 |
| Mobility of cells | 2,19E-02 | CADM1,TM4SF1 | 2 |
| Cell movement of chronic lymphocytic leukemia cells | 2,63E-02 | HMMR | 1 |
| Cell movement of colon carcinoma cells | 2,63E-02 | L1CAM | 1 |
| Cell movement of luminal epithelial cells | 2,63E-02 | HGF | 1 |
| Cell movement of myoepithelial cells | 2,63E-02 | HGF | 1 |
| Cell movement of progenitor cells | 2,63E-02 | HGF | 1 |
| Contact repulsion of axons | 2,63E-02 | L1CAM | 1 |
| Cytokinesis of fibrosarcoma cell lines | 2,63E-02 | TOP2A | 1 |
| Invasion of astrocytes | 2,63E-02 | CHI3L1 | 1 |
| Invasion of melanocytes | 2,63E-02 | HGF | 1 |
| **Replication, recombination and repair of dna (45 molecules)** | | |  |
| Synthesis of dna | 6,71E-05 | BIRC5,CCNA2,CDKN2C,CDT1,CHRM3,EDN1,EGF (includes EG:13645),FBLN5,FGF1,FLT1,HBEGF,HGF,IGFBP5,PDGFRA,RGS4,TGFB2,TNC (includes EG:116640) | 17 |
| Segregation of chromosomes | 7,23E-09 | BUB1 (includes EG:100307076),CCNA2,CCNB1,CCNB2,CENPF,CENPW,KIF2C,NCAPG,NDC80,NEK2,NUSAP1,SPC25 (includes EG:100144563),TOP2A | 13 |
| Alignment of chromosomes | 3,12E-07 | BIRC5,CCNA2,DLGAP5,KIF14,KIF2C,NCAPG,TTK | 7 |
| Dna damage | 3,24E-03 | BIRC5,OLR1,PBK,RRM2,RUNX1,RUNX1T1,TOP2A | 7 |
| Chromosomal congression of chromosomes | 3,02E-05 | KIF14,KIF2C,NDC80,SGOL2 | 4 |
| Condensation of chromosomes | 8,50E-04 | KIF4A,NCAPG,NUSAP1,TOP2A | 4 |
| Spindle checkpoint of cells | 1,07E-03 | BIRC5,BUB1 (includes EG:100307076),DLGAP5,TTK | 4 |
| Incorporation of thymidine | 1,56E-02 | EDN1,EGF (includes EG:13645),HBEGF,IGFBP5 | 4 |
| Formation of mitotic spindle | 1,71E-02 | BIRC5,KIF2C,KIF4A,NEK2 | 4 |
| Hydrolysis of nucleotide | 2,21E-02 | ATP7B,CDK1,IQGAP2,RGS4 | 4 |
| Segregation of sister chromatids | 3,32E-03 | CCNA2,NDC80,NUSAP1 | 3 |
| Hydrolysis of gtp | 1,91E-02 | CDK1,IQGAP2,RGS4 | 3 |
| Organization of mitotic spindle | 2,16E-02 | NDC80,SPC25 (includes EG:100144563),TTK | 3 |
| Checkpoint control of mitotic spindle | 6,89E-04 | CDC20 (includes EG:107995),NDC80 | 2 |
| Formation of nuclear envelope | 2,03E-03 | CDK1,EGF (includes EG:13645) | 2 |
| Alignment of sister chromatids | 3,99E-03 | NDC80,TOP2A | 2 |
| Elongation of mitotic spindle | 3,99E-03 | PRC1 (includes EG:233406),SPC25 (includes EG:100144563) | 2 |
| Arrest in spindle checkpoint of cervical cancer cell lines | 2,63E-02 | DLGAP5 | 1 |
| Cleavage of pbr322 plasmid | 2,63E-02 | TOP2A | 1 |
| Deamination of deoxycytidine | 2,63E-02 | APOBEC3G | 1 |
| Deamination of deoxyuridine | 2,63E-02 | APOBEC3G | 1 |
| Delay in alignment of chromosomes | 2,63E-02 | CCNA2 | 1 |
| Delay in segregation of sister chromatids | 2,63E-02 | CCNA2 | 1 |
| Hydroxylation of dna | 2,63E-02 | EGF (includes EG:13645) | 1 |
| Hypercondensation of chromosomes | 2,63E-02 | KIF4A | 1 |
| Ligation of pbr322 plasmid | 2,63E-02 | TOP2A | 1 |
